# Supplementary material for: Mind the ramp: Association between early life ramp use and spatial cognition in laying hen pullets
Source: PLoS One. 2024 Apr 26;19(4):e0302454. doi: 10.1371/journal.pone.0302454 (PMC11051627; doi:10.1371/journal.pone.0302454)
Supplement: Attachment — Submitted filename: PONE-D-23-28746_R1_reviewer.pdf [file pone.0302454.s003.pdf]

PLOS ONE

Mind the ramp: Association between early life ramp use and spatial cognition in laying hen pullets.

--Manuscript Draft--

**Manuscript Number:**	PONE-D-23-28746R1
**Article Type:**	Research Article
**Full Title:**	Mind the ramp: Association between early life ramp use and spatial cognition in laying hen pullets.
**Short Title:**	Association between early life ramp use and spatial cognition in laying hen pullets.
**Corresponding Author:**	Alex Johny<br>University of Bern Vetsuisse Faculty: Universitat Bern Vetsuisse Fakultat<br>Bern, SWITZERLAND
**Keywords:**	chicken, rearing environment, habitat complexity, behaviour, holeboard test
**Abstract:**	Ramps facilitate earlier access to complex environments and increase early life voluntary exercise, which may positively affect the cognitive development of chickens. This study focused on quantifying individual differences in ramp use and its impact on spatial cognition of laying hen pullets. Sixteen identical pens were housed with Lohmann Selected Leghorn (LSL) chicks of which eight chicks from each pen were colour marked from one day of age (DoA) to serve as focal birds. We quantified overall ramp use (walk/run, wing-assisted incline running, and jump/fly to and from ramps) by scan sampling recorded videos for 6, 10, 12, 20, 27, 41, and 55 DoA for all focal birds. From 56 to 95 DoA, long and short-term spatial memory of three focal birds per pen were assessed in a holeboard test in three consecutive phases: cued, uncued and reversal. Mixed model analysis showed no consistency in average ramp use per day for individual birds. However, we found that the spatial cognitive abilities of the birds were linked to differences in ramp use frequency averaged across all observation days. Birds with higher ramp use made fewer reference (Estimate ± Confidence Interval = 0.94 [0.88, 0.99], p = 0.08) and working memory errors (Est ± CI = 0.77 [0.59, 1.00], p = 0.06) in the cued phase than birds with lower ramp use. In contrast, birds with higher ramp use made more reference memory errors (Est ± CI = 1.10 [1.01, 1.20], p = 0.05) in the reversal phase. Birds with higher ramp use also made more reference memory errors compared to birds with lower ramp use as the phases changed from cued to uncued (p = 0.001). Our results indicate that there might be a relationship between early life ramp use and spatial cognition of laying hens.
**Order of Authors:**	Alex Johny
	Andrew Michael Janczak
	Janicke Nordgreen
	Michael Jeffery Toscano
	Stratmann Ariane
**Response to Reviewers:**	Editor:<br>Discussion of the results requires more depth in interpreting the results.<br>We have added new portions (L 628 - 640), removed certain sections (L 611 – 627, 642- 666) and rewritten (L 567 - 610) to bring more depth in interpreting results as well as make the discussion succinct.<br>The statistical model needs clarification.<br>We used a combination of estimates, confidence intervals and p values to describe relationship with response and explanatory variables as described in L341 - 342., rather than use p < 0.05 as cut-off. We also wanted to try the approach of providing as many results as possible without the use of terms such as statistically significant and non-significant as advocated by Wasserstein et al., 2019, Hulbert et al., 2019, Berner and Amrhein (2022) etc. We followed a similar method as Rosenberger et al. (2022) to report our results.<br>The entire manuscript needs a brief, more in-depth and clearer writing, with recent references cited.

Powered by Editorial Manager® and ProduXion Manager® from Aries Systems Corporation

We have removed and rephrased parts in all the sections to improve the clarity as well reduce the length of the manuscript. We have also added more references to support out claims.
Reviewer #1:
This study tested the association between spatial memory and learning (holeboard test) and voluntary ramp use in laying hen pullets. They found no within-bird consistency for ramp use behaviour. They did find relationships between ramp use frequency and the performance in the holeboard test, some of them supporting their predictions and some to the contrary. The experimental design is sound, though the statistical analyses are perhaps unnecessarily complicated. If no statistician was involved, I would recommend that the authors consult a statistician. If there was a statistician involved, then the section on statistics needs a bit of clarification. The manuscript is well written though it is lengthy and unnecessarily wordy and repetitious at times. My biggest concern is the discussion. There appears to be a lack of references and the structure can be improved by reducing redundant elaborations. Once cleaned up, this manuscript can be a valuable contribution to the laying hen literature.
Thanks for the detailed review and valuable feedback. After talking to a researcher with statistical expertise we have decided to remove the repeatability analysist because the sample size is on the lower end to calculate repeatability of ramp use. We have hence removed parts pertaining to repeatability analysis in all section of the manuscript. We also reanalysed the phase change analysis to include mean ramp use frequency (MRF) as a continuous variable. This analysis included all the birds unlike the previous analysis which used ramp use a categorical variable with high (ten birds with highest MRF) and low ramp users (ten birds with lowest MRF).
Major Comments
Stay consistent with the numbering (spelling out numbers under 10), please check the whole manuscript for inconsistencies.
Thanks. We have changed the numbering accordingly.
Abstract
You need to introduce terminology somewhere: chicks vs hens vs pullets. This journal is not poultry-specific.
We don’t think it is necessary. They are universally used terms and not specific to the paper and can be easily obtained if the reader does a quick web search.
L27: what analyses? Give a model or analysis type to help understand the results.
We have changed it to Mixed-model analysis.
L30-33: These results are a bit confusing. First, they are trends/tendencies and not significant results, so you should state that. Second, it is unclear what the unit of those estimates are.
The estimates are back transformed, so it is integer scale (number of errors were positive integers including zero). The estimate here is a slope. For example, a 0.94 estimate means for one unit of increase in ramp use there is a 6% decrease in errors made. The change in errors between x and x+n ramp use would be n*0.6.
For interpretation of statistics, we did not use $p < 0.05$ as cut-off. Rather we used a combination of estimates, confidence intervals and p values to describe relationship with variables as described in L341 - 342. We also wanted to try the approach of providing as many results as possible without the use of terms such as statistically significant and non-significant as advocated by Wasserstein et al., 2019, Hulbert et al., 2019 etc.,
L37: Please state why this would be relevant/ interesting.
With the given word limit, it is unfortunately not possible to have another sentence in the abstract, without losing information that we think are critical. Moreover, the relevance of the topic is covered in the introduction part.
Introduction
L63: I would suggest exchanging the Campbell reference with Widowski and Torrey’s book chapter on rearing young birds for adaptability:
https://linkinghub.elsevier.com/retrieve/pii/B9780081009154000038. This statement refers to a shift in research interest based on the shift in management. Both the Ferreira and Widowski and Torrey references are revisions of the literature and the importance of research in this area. The Campbell paper is an experiment that can be mentioned later when discussing specific findings.
Thank you for the suggestion. We have changed it accordingly.
L108-116: This has to go before the research question/objectives (L106-107). Then, add lines 106-107 to the beginning of the next paragraph that contains your

predictions.
Thanks for the feedback. We have rearranged the sentences and rephrased the research objective (L125 - 128)
Methods
L130-131: It's a bit confusing to point to your other paper for the pen schematics. I immediately looked at your Figure 1 and expected to see a pen. I would suggest you provide schematics or a photo here. Alternatively, add the reference after your description and say that more information and schematics can be found in Johny et al. (28). But I would prefer a photo or schematics in this paper.
We have added the sentence you suggested in L 153. Thank you.
L157: How did you choose your random sample? How did you make sure there was no bias (e.g., first catches could be bold, last in the box could be most fearful).
Its not a random sample. Chicks were arbitrarily assigned to the pens. At the time of population, they came in crates. We picked the birds from the crates arbitrarily, applied colour marks and then introduced them to the pens. Since they were all constrained in small crates which did not allow any movement, we think there were no bias due to the catch order.
L169-173: Unclear what you mean here. What treatments? How were they managed? Why is this important?
The treatments were three different artificial cues that were aimed at improving ramp use and a control with no cue that were part of an additional experiment (Johny et al., 2023). The treatment groups influenced the variation in use of ramps, in addition to the individual-level differences. So, we added the treatment as control variable to account for the variation in ramp use due to the cues. We have added this information in L186 – 191.
L292-295: This section needs clarification. I do not understand it. Do you mean the slopes in your statistical model? I also don't understand why you group the birds into high and low ramp users. I though your sample was random. Did you have two clusters? Elaborate on this and on why you chose to create these categories instead of using their actual frequencies.
We changed the analysis and included MRF as a continuous variable like the rest of the analysis. This allowed us to use all the birds unlike the previous analysis which included ramp use as categorical variable with high (ten birds with highest MRF) and low ramp users (ten birds with lowest MRF).
L306-308: Why did you use bootstrapping to get the main effect p-values? To the best of my knowledge, this method is used to reduce a model and get p-values for reduced factors only. The main effects can be assessed as part of the model. Was a statistician involved in this analysis? If so, I will retract my comment. Otherwise, I would like you to consult a statistician.
We used the method suggested by PD Dr. Lorenz Gygax, HU Berlin. Although we did not consult him specifically for this experiment, one of the authors took a statistical course from him. We performed the analysis according to the framework provided in the course. Some major additions to the statistics from the course were:
1.Setting sum contrasts for the categorical variables and standardising continuous variables which provide interpretable main effects, even in the presence of interaction effects. This approach provides parameters such as p-values, estimates and confidence intervals of all main effects without the need to dropping interaction terms or reducing the global model that was initially chosen because of their biological relevance.
2.Using bootstrapping to obtain p-values, which are more conservative than likelihood ratio test, due to its robustness in dealing with influential points (Luke, 2016).
L326: If you only include non-zero observations, are you not biasing the analysis? What if a bird uses it occasionally? As I understand it, you would overestimate the repeatability by only including the observations where the bird happened to use the ramps and excluding observations where it didn't.
We removed the repeatability analysis after consulting a researcher with statistical expertise because our sample size is on the lower limits required for repeatability analysis.
However, to answer your question, we did two different analyses to account for the huge number of zeroes. The first analysis is a binary model and had zeroes included in it. As it was binary, ramp use was coded as if a focal bird used ramps in a particular bout (1) or not (0). In the second analysis, we excluded the observations where an individual birds did not use the ramps. In that case, your argument is right. Taking out zeroes increases the variation in data and since repeatability is the ratio of individual

Powered by Editorial Manager® and ProduXion Manager® from Aries Systems Corporation

level variance to total variance, one would expect an increase in repeatability. But as was mentioned in the discussion section that is now removed, the total variability of data is less, because of the 3 min observation window we used. That is likely a reason why we still didn't see repeatability in ramp use, even after excluding zeroes.

Results

I recommend making the sentence about the effects of $p < 0.01$ less vague. If you decide to report tendencies (which you need to clarify in the stats section), report them without embellishments. E.g., 'errors tended to decrease with trial number' instead of 'with very limited statistical support, Y made slightly fewer errors'.

As we wrote in response to your comment in the abstract part, we are using the effect size, uncertainty intervals and p-values to describe our results, rather than using the p-value cut-off of <0.05. So, we also opt to change the use of terms such as significant ($p<0.05$) and trend ($0.05<p< \sim0.08$). We do understand that this is not the norm in the field. We used a similar method as in Rosenberger et. al, 2022, to describe our results.

L385-386: I am still confused about where these high and low classifications come from… and now there is a medium group. Was I supposed to know that? Maybe I missed it.

We wanted to provide the estimated marginal means (EMM) for the number of errors made. We selected birds with low, median, and high MRF as a few examples to provide the EMMs, instead of providing EMMs of all MRF observations. Additionally, we removed the low and high ramp user category from the phase change analysis as mentioned earlier.

L448: I am not sure if removing these data points is appropriate. What if the group you claim learned faster actually had trials in there that were not completed? I recommend analysing it with the complete data set or declaring which ramp use groups the omitted data points came from if they were exclusively from low ramp users.

9, 1 and 7 out of 480, 960 and 370 observations from cued, uncued and reversal phases, respectively were omitted, which we think will have a negligible effect on the analysis.

Discussion

There is a general lack of references supporting sweeping statements in this discussion. Please put your results into the context of existing literature and how your study adds to the knowledge base. There is also a lot of repetition that could be cut down (e.g., repeating of your own results in every paragraph).

Thanks for the feedback. We have restructured the discussion based on your comments.

L497-505: But your birds' ramp use behaviour was inconsistent over time. How would that translate to personality, which is what you are implying but not spelling out here.

Our analysis did not reveal consistency in ramp use. But given results from Montalcini et al., 2023a, Montalcini et al., 2022, we would expect this behaviour to be repeatable. Also the movement patterns in aviaries across individuals have been shown to be consistent (Rufener et al., 2023, Montalcini et al., 2023a, Montalcini et al.,2023b). We think it's a limitation of the method (three-minute observation period) as well the low sample size (which led us to remove the analysis on repeatability of ramp use) which rendered the ramp use behaviour non-repeatable in our analysis.

The argument we make is that differences in ramp use (a movement pattern) and spatial cognition could be driven by individual differences in activity, exploration, and fearfulness. The differences in movement behaviours have been shown to correlate to differences in behaviours such as activity, exploration as mentioned in L533-538. On that note, it is possible that ramp use might also be driven by individual differences in activity (Rufener et al., 2023) and exploration (de Haas et al., 2017; Rodenburg et al., 2003) which has been shown to be repeatable in hens.

L568-572: Why would this only affect the uncued phase? If HR pullets do better in the cued but not the uncued phase, would this mean there is no difference in spatial learning (location) but only in association learning (colour)?

Yes, this is one possible explanation. We have added this in L627- 640. Thanks for the suggestion.

L580-583: WoA 19 is not the start of peak of lay, and WoA25 is not sexual maturity… it would be the reverse. Are you sure these ages and the terminology have the correct references? I also think that these are two different aspects. Sexual maturity would end the ontogenesis and be accompanied by hormonal changes and, in the case of laying hens, also with a change in housing. The peak of lay is well after that and is not initiated by hormonal or housing changes. I would be hesitant to group those two results and make one statement about behavioural consistency during ontogeny.

Powered by Editorial Manager® and ProduXion Manager® from Aries Systems Corporation

As we removed the ramp use repeatability analysis, this part of discussion is no longer in the manuscript.
L590-593: As ramp use was the behaviour in focus, why choose an observation period that you think is too short? If you had good reason to assume it was appropriate before you started, you should not backtrack now but rather accept your results. If you have reason to believe it was inappropriate based on new information after the fact, please add a section with limitations and explain your initial rationale and what changed.
This part is removed from the manuscript as it part of the repeatability analysis.
L593-596: No. You tested a hypothesis and did not reject the null hypothesis. If you have reason to assume a type 2 error, you must provide it. Otherwise, you cannot state your initial prediction after not confirming it. Also, what is 'short-term' consistency even? If you introduce a vague term like that, you have to define it and back it up with literature.
This part is removed from the manuscript as it part of the repeatability analysis.
Minor Comments
Do not be alarmed by the number of comments, most are very small and should be easily addressed.
Abstract
L19: 'affect the brain' is very vague. Be more specific or don't mention the brain.
Removed the brain part.
L25: 'observation period' what ages are those? Instead of observation period. Give an age range.
Added the observation days.
L25-26: 'spatial cognition' be more specific on what aspect of spatial cognition this test is assessing.
Changed it to long and short-term spatial memories.
L26: The verb refers to spatial cognition not to the birds. Change 'were' to 'was'.
Now it is 'long and short-term spatial memories' instead of spatial cognition.
L29: 'averaged across all observation days' is redundant as you already stated that you analyzed the means.
Removed 'mean'.
L30: spell out Est and CI when you first use it.
Done.
Introduction
L67-68: I don't mean to increase my own citation but there is newer literature on this which also happened to be mine… I'm not telling you to include it, but you might want to consider it. See https://doi.org/10.1016/j.applanim.2023.105997
Added the citation.
L69-70: Same comment as above. Consider including this reference as it is more recent: https://doi.org/10.1038/s41598-023-35956-1
Added the citation.
L88-91: This sentence is too long and complex and I am not sure what the content is. Not only do they use ramps to transition between tiers but also to… what?
Rephrased the sentence for clarity.
L96: The 'inter' part of inter-individual differences is redundant as there can be no intra-individual difference in cognition as far as I'm aware (unless you do a before/after thing). Adding the 'inter' suffix draws attention and seems unnecessarily confusing.
There are intra-individual differences in cognition. And inter- individual differences is a commonly used term in field of behavioural sciences. So, we don't think it is unnecessarily confusing.
L96-97: This sentence is a bit awkward. I would suggest rewording it. Here are two suggestions, but it's obviously up to you. 'There has been a recent peak in the interest in individual cognitive differences and their importance in the study of cognition.' OR 'Individual cognitive differences and their importance in the study of cognition have received a recent increase in interest/attention.'
Rephrased using your second suggestion.
L97-98: Add a comma after surfaces
We have rephrased the sentence now.
L99-100: Personally, I would say locomotion instead of movement behaviour, but that's just a suggestion. Or are you talking about types of locomotion?
We are not talking about types/modes of locomotion, such walking or running, but rather about patterns of movement such as range use and space use within the aviary. We think movement behaviour captures this more than locomotion, hence we prefer to use movement behaviour.

Powered by Editorial Manager® and ProduXion Manager® from Aries Systems Corporation

L99-101: ‘individual differences’ three times in one sentence. I would change the last one to say ‘can lead to differences in developmental trajectories’. The whole sentence is a bit vague. I would say ‘results in’ instead of ‘can result in’ as the start of the sentence already states that this is a possibility and not a fact.
Rephrased for clarity.
L103: ‘Hence, it is important…’, but that might be a personal preference.
Changed accordingly.
L104: Again, just say individual differences, not individual-level differences (redundant). Or rephrase to say ‘differences at the individual level’.
Rephrased accordingly.
L106: The ‘inter’ part of ‘inter-individual variation’ is redundant.
We disagree for the reason explained earlier.
L107: Here too, I find that spatial cognition is too broad a term for a holeboard test. Say what aspect of spatial cognition you were testing.
Rephrased as spatial memories.
L117-120: this sentence is too long. Break this into two or even three sentences.
The sentence was removed.
Methods
L129-130: Either say ‘All pens housed 22 birds’ or ‘Pens housed 22 birds each’. Using ‘all’ and ‘each’ is too unnecessary.
Thanks. Rephrased according to your suggestion.
L136-137: half of this sentence is redundant. The pen floors were covered in wood shavings (dimensions). OR The pen floors were littered with wood shavings (dimensions).
Done.
L138: plastic barriers made of metal sheets? Generally, that sentence is a bit wordy. Simplify
Rephrased the sentence and split into two for clarity.
L141: why were they confined for the first 4 days? I know why, but you need to spell it out (briefly!) for non-poultry people.
Yes, thank you. Added one sentence as explanation in L155 - 156.
L142: What do you mean by feeder plate? Like a flat dish? Can you give a description?
Added dimensions.
L147: It would be nice to have the LUX. I’m assuming it changed with age, so you could add it to the sentence where you give the change in light period length.
Added the information in L163.
L151: Wordy -> five minutes at dawn and 30 minutes at dusk.
Thanks. Changed according to suggestion.
L151: Why say that daylight was blocked? It would suffice to say there was none. Or even better, add ‘there was no natural light’ to your lighting sentence.
Deleted the line ‘daylight was blocked’ and added ‘there was no natural light’ in L 164.
L167: How do you know there was no aggression? Anecdotal observations? Injuries?
We did not observe any aggression towards colour marked birds during the video analysis and animal checks during management.
L178-179: The software ‘recorded the behaviour’? Did it code it? Did it record locations? How? Did it record birds, and you recorded the behaviour?
The software recorded the videos, and we analysed the videos. Changed wording to clarify.
L180-186: There are many repetitions here and it’s wordier than necessary. I suggest rewriting this passage, making it more concise. E.g., “…: the inter-tier transitions (up and down combined) and active behaviours on the ramp (walking, running, wing-assisted incline running, jumping to or from the ramp without changing tier; see ethogram in Johny et al (28)).
Reworded to make it concise.
L207-208: Hence, birds were visually but not auditorily isolated.
Thanks, changed as per suggestion.
L209-211: Don’t repeat the information that is in the figure. The arena held eight circles containing small cups to provide the reward (grape pieces).
We removed most of the measurements, but retained the previous text as we think it is a good description of the arena.
Figure 1: It might help to visualize that 1B represents half of 1A. E.g., you could draw lines from the corners of one pen to the corners of the arena.
We added more information in the figure description. We think drawing the lines from corners of pen might render the figure more cluttered.

Powered by Editorial Manager® and ProduXion Manager® from Aries Systems Corporation

L220 ff: This should go into the paragraph 'Cue phase trial'. Continuing to habituation from here seems like a step back. I suggest you rename this paragraph to 'Arenas' and only include the arena description. It would make more sense to go from 'Arenas'-> 'Habituation' -> testing procedure.
The information given is important to make sense of the habituation and test phase. For ex., "the birds were habituated and tested in the same arena".
The paragraph contains certain procedures that were common for the habituation and the test phases. So, we prefer to keep it as 'Arena and procedure'.
L234: Did you ensure that your focal birds had eaten the grapes during the habituation period?
Yes, the information is given L 257 – 258.
L234: How did you choose the focal birds? Convenience sampling?
Changed the wording from 'randomly chosen' to 'arbitrarily chosen'. So, it is convenience sampling.
L254: Did you use a learning criterion, or did all birds eat all grapes in all trials?
There was no learning criterion in the cued phase. All birds did not eat all grapes in all trials. All birds ate from all rewarded cups in almost all trials. However, calculation of the memory errors doesn't require birds to visit all the rewarded cups.
L259: I suggest you move the information on the learning criterion of the uncued phase into the section about the uncued phase. Also, if birds reached the criterion early, did they still do all 20 trials?
The learning criterion was used only for the selecting birds for the reversal phase and we did not use the learning criterion for any analysis including the uncued phase. So, this information is only relevant for the reversal phase and thus we prefer to keep it in the 'reversal' section.
All the birds had 20 trials, no matter when they reached the learning criterion.
L283: what is the unit here?
Seconds. Added the information.
L288 ff: To delete the very long and complicated sentence on L288-292, you could include the number of trials like so: "… , we compared errors of each bird from the three trials before and three trials after a phase change …" This is only a suggestion.
Thanks for the suggestion. Changed accordingly.
L302-305: Out of curiosity, what was wrong with your main effects before? I have not seen this to be necessary before, does it not over-specify your models?
Centring the data to zero or using sum-contrasts does not affect fit of the model. The aim of performing this step was to calculate effect sizes and p-values while retaining the interaction term in the model (Schad et al., 2019). We did not drop the interaction term if it was insignificant, as we wanted to stick with our global model.
L332: what treatments? You cannot expect people to read other papers to understand this one.
We have added the information about treatments in L 187 – 191.
Results
Furthermore, many repetitions can be avoided by adding units (count/3min). The reader can be trusted to remember that there were 8 bouts per 7 observation days after you mentioned it once.
L365-368: Repetitions and inconsistencies. You are giving the mean, min, and max count/3min for the individual observations but the mean you call a frequency and the min and max you don't specify. This could be more concise and clarified. Also, you repeat '8 bouts per 7 observations days' which is unnecessary. Furthermore, you are inconsistent with the numbering. It makes the passage more complicated and harder to understand than need be.
Rephrased the sentences. Moreover, the paragraph is simpler now because we removed the repeatability analysis.
L388: I suggest you delete the word 'seem'. It either influenced it or not. You can even say you didn't detect an effect of… but be less vague.
Thanks, we deleted 'seem'.
Figures 2-3: I recommend only showing the statistically significant results (Figure 3); the others are sufficiently described in the text (also for Figure 4). If you choose to show all plots, I recommend merging 2 and 3 into one plot with two panels, as the caption is the same.
This is an exploratory study where we are describing the results. Moreover, as mentioned in the methods section, we have refrained from the use of just p-value based inference of results.
Figures 2-4: I am confused about the colour changes. The grading is according to the

Powered by Editorial Manager® and ProduXion Manager® from Aries Systems Corporation

	Y-axis and does not add information. It made me think there were different treatments.<br>There is a description of the colour gradient in the figure caption. So, we don't think it's confusing.<br>L459-460: You have been using HR and LR for a long time at this point; no sense in spelling it out in this bracket. The bracket might be superfluous anyway, especially if you added a section explaining how you defined these groups, as I recommended earlier.<br>We have removed the HR and LR groups in the analysis.<br>Table 1: Please add indicators for significant differences (e.g., superscript letters).<br>We did not perform post-hoc tests as mentioned in L345-346.<br>Discussion<br>L478: specify the aspect of spatial cognition you assessed.<br>Yes, we have changed it to long and short-term spatial memories.<br>L479: specify the early life period. If you refer to the first eight weeks, move it up from the end of the sentence.<br>Early life period is now specified.<br>L481-488: Add which results supported your hypotheses and which didn't.<br>As this was an exploratory study, we did not have any hypotheses. We removed the expectation based on current literature that we had mentioned in L 128- 132, to avoid any confusion.<br>L491: Add reference supporting your statements.<br>This sentence has been removed.<br>L518-530: Most of this is not new information. This whole paragraph seems redundant. Add the main points to the last paragraph where needed and delete this paragraph. There is also a lack of references backing your statements.<br>Thanks for your suggestion. We have combined both paragraphs in L566 - 580.<br>L587-590: The cattle example seems out of place. I recommend you remove it.<br>Removed.<br>L597-614: This paragraph seems out of place and irrelevant. It adds no new information and does not support your suggested interpretations. I recommend deleting it as it dilutes your discussion.<br>As holeboard is not yet widely used to measure spatial cognition we wanted to compare the scores from few other studies that have used holeboard test in hens for external validation as mentioned in L667-669. Hence we prefer to keep this paragraph.<br>L615-620: This would be very interesting sections on limitations. However, I am missing a discussion on the consequences of these limitations. How could these limitations affect your results and interpretation?<br>L627-629: Add references to all of these statements.<br>We have added the references wherever applicable.<br>L634-636: That was done here: https://doi.org/10.1016/j.applanim.2023.105997 and to a degree here: https://doi.org/10.1038/s41598-023-35956-1.<br>Agree. But it is still critical to have more studies on the early rearing complexity and its impact on spatial skills. Also, it might also be interesting to test the effect of conditions that are more complex than the existing aviary setups, to understand if the current rearing aviaries are complex enough to trigger major developmental changes.<br>Reviewer #2:<br>Consideration of welfare in laying hens is good area of research, specially when battery cages are not much in use in Europe. Overall, this is a well written paper, however, it needs some revisions. Some of these are mentioned in the attached file.<br>Thank you for your suggestions.<br>In methodolgy, no. Of birds is 351; suggesting one pen had 21 birds.<br>Thank you. One pen only had 21 pens from the beginning. Since this information is not very relevant, we have removed it from abstract. The animal numbers are explained in detail in the methods section.<br>Abstract should be self explanaitry. There should be some detail of these phases.<br>For the given word limit, adding details on the phases is not possible without dropping other relevant information. So, we choose not to add more details on the phases.<br>If there was a mortality, then please elaborate it as well.<br>There was no mortality. The reason for 21 chicks is now added in L 146- 147.
**Additional Information:**	
**Question**	**Response**
**Financial Disclosure**	AJ: The work was supported by the European Union's Horizon 2020 research and

Enter a financial disclosure statement that describes the sources of funding for the work included in this submission. Review the submission guidelines for detailed requirements. View published research articles from *PLOS ONE* for specific examples.<br><br>This statement is required for submission and **will appear in the published article** if the submission is accepted. Please make sure it is accurate.<br><br>**Unfunded studies**<br>Enter: *The author(s) received no specific funding for this work.*<br><br>**Funded studies**<br>Enter a statement with the following details:<br>• Initials of the authors who received each award<br>• Grant numbers awarded to each author<br>• The full name of each funder<br>• URL of each funder website<br>• Did the sponsors or funders play any role in the study design, data collection and analysis, decision to publish, or preparation of the manuscript?<br>• **NO** - Include this sentence at the end of your statement: *The funders had no role in study design, data collection and analysis, decision to publish, or preparation of the manuscript.*<br>• **YES** - Specify the role(s) played.<br><br>* typeset	innovation programme under the Marie Skłodowska-Curie grant agreement No 812777. The funders had no role in study design, data collection and analysis, decision to publish, or preparation of the manuscript
**Competing Interests**<br><br>Use the instructions below to enter a competing interest statement for this submission. On behalf of all authors, disclose any competing interests that could be perceived to bias this work—acknowledging all financial support and any other relevant financial or non-financial competing interests.<br><br>This statement is required for submission and **will appear in the published article** if	The authors have declared that no competing interests exist.

the submission is accepted. Please make sure it is accurate and that any funding sources listed in your Funding Information later in the submission form are also declared in your Financial Disclosure statement.<br><br>View published research articles from *PLOS ONE* for specific examples.<br><br>**NO authors have competing interests**<br><br>Enter: *The authors have declared that no competing interests exist.*<br><br>**Authors with competing interests**<br><br>Enter competing interest details beginning with this statement:<br><br>*I have read the journal's policy and the authors of this manuscript have the following competing interests: [insert competing interests here]*<br><br>* typeset	
**Ethics Statement**<br><br>Enter an ethics statement for this submission. This statement is required if the study involved:<br><br>• Human participants<br>• Human specimens or tissue<br>• Vertebrate animals or cephalopods<br>• Vertebrate embryos or tissues<br>• Field research<br><br>Write "N/A" if the submission does not require an ethics statement.<br><br>General guidance is provided below. Consult the submission guidelines for detailed instructions. **Make sure that all information entered here is included in the Methods section of the manuscript.**	The experiment met the federal and cantonal regulations for the ethical treatment of animals involved in research and was approved by the Veterinary Office of the Canton of Bern, Switzerland (BE 106/19).

**Format for specific study types**<br><br>**Human Subject Research (involving human participants and/or tissue)**<br>• Give the name of the institutional review board or ethics committee that approved the study<br>• Include the approval number and/or a statement indicating approval of this research<br>• Indicate the form of consent obtained (written/oral) or the reason that consent was not obtained (e.g. the data were analyzed anonymously)<br><br>**Animal Research (involving vertebrate animals, embryos or tissues)**<br>• Provide the name of the Institutional Animal Care and Use Committee (IACUC) or other relevant ethics board that reviewed the study protocol, and indicate whether they approved this research or granted a formal waiver of ethical approval<br>• Include an approval number if one was obtained<br>• If the study involved *non-human primates*, add *additional details* about animal welfare and steps taken to ameliorate suffering<br>• If anesthesia, euthanasia, or any kind of animal sacrifice is part of the study, include briefly which substances and/or methods were applied<br><br>**Field Research**<br><br>Include the following details if this study involves the collection of plant, animal, or other materials from a natural setting:<br>• Field permit number<br>• Name of the institution or relevant body that granted permission	
**Data Availability**<br><br>Authors are required to make all data underlying the findings described fully available, without restriction, and from the time of publication. PLOS allows rare exceptions to address legal and ethical concerns. See the PLOS Data Policy and FAQ for detailed information.	Yes - all data are fully available without restriction

A Data Availability Statement describing where the data can be found is required at submission. Your answers to this question constitute the Data Availability Statement and **will be published in the article**, if accepted.<br><br>**Important:** Stating 'data available on request from the author' is not sufficient. If your data are only available upon request, select 'No' for the first question and explain your exceptional situation in the text box.<br><br>Do the authors confirm that all data underlying the findings described in their manuscript are fully available without restriction?	
**Describe where the data may be found in full sentences. If you are copying our sample text, replace any instances of XXX with the appropriate details.**<br><br>• If the data are **held or will be held in a public repository**, include URLs, accession numbers or DOIs. If this information will only be available after acceptance, indicate this by ticking the box below. For example: *All XXX files are available from the XXX database (accession number(s) XXX, XXX.).*<br>• If the data are all contained **within the manuscript and/or Supporting Information files**, enter the following: *All relevant data are within the manuscript and its Supporting Information files.*<br>• If neither of these applies but you are able to provide **details of access elsewhere**, with or without limitations, please do so. For example:<br><br>*Data cannot be shared publicly because of [XXX]. Data are available from the XXX Institutional Data Access / Ethics Committee (contact via XXX) for researchers who meet the criteria for access to confidential data.*<br><br>*The data underlying the results presented in the study are available from (include the name of the third party*	The data and code for all analysis can be found at https://osf.io/9j2g8

and contact information or URL).<br>• This text is appropriate if the data are owned by a third party and authors do not have permission to share the data.<br><br>* typeset	
Additional data availability information:	

Powered by Editorial Manager® and ProduXion Manager® from Aries Systems Corporation

Manuscript Click here to access/download;Manuscript;Manuscript.docx

Mind the ramp: Association between early life ramp use and spatial cognition in laying hen pullets.

Alex Johny[1, 2*], Andrew M. Janczak[3], Janicke Nordgreen[4], Michael J. Toscano[1], Ariane Stratmann[1, #]

[1]Centre for Proper Housing of Poultry and Rabbits, VPHI Institute, University of Bern, Zollikofen, Switzerland

[2]Graduate school of Cellular and Biomedical Sciences, University of Bern, Bern, 3012 Switzerland

[3] Department of Production Animal Clinical Science, Faculty of Veterinary Medicine, Norwegian University of Life Sciences (NMBU), Ås, Norway

[4] Department of Paraclinical Sciences, Faculty of Veterinary Medicine, Norwegian University of Life Sciences, Ås, Norway

[#]Current Address: Centre for Proper Housing of Ruminants and Pigs, Federal Food Safety and Veterinary Office, Agroscope, Posieux, Switzerland.

*Corresponding author

Email: alex.johny@unibe.ch

Abstract

Ramps facilitate earlier access to complex environments and increase early life voluntary exercise, which may positively affect the cognitive development of chickens. This study focused on quantifying individual differences in ramp use and its impact on spatial cognition of laying hen pullets. Sixteen identical pens were housed with Lohmann Selected Leghorn (LSL) chicks of which eight chicks from each pen were colour marked from one day of age (DoA) to serve as focal birds. We quantified overall ramp use (walk/run, wing-assisted incline running, and jump/fly to and from ramps) by scan sampling recorded videos for 6, 10, 12, 20, 27, 41, and 55 DoA for all focal birds. From 56 to 95 DoA, long and short-term spatial memory of three focal birds per pen were assessed in a holeboard test in three consecutive phases: cued, uncued and reversal. Mixed model analysis showed no consistency in average ramp use per day for individual birds. However, we found that the spatial cognitive abilities of the birds were linked to differences in ramp use frequency averaged across all observation days. Birds with higher ramp use made fewer reference (Estimate ± Confidence Interval = 0.94 [0.88, 0.99], $p = 0.08$) and working memory errors (Est ± CI = 0.77 [0.59, 1.00], $p = 0.06$) in the cued phase than birds with lower ramp use. In contrast, birds with higher ramp use made more reference memory errors (Est ± CI = 1.10 [1.01, 1.20], $p = 0.05$) in the reversal phase. Birds with higher ramp use also made more reference memory errors compared to birds with lower ramp use as the phases changed from cued to uncued ($p = 0.001$). Our results indicate that there might be a relationship between early life ramp use and spatial cognition of laying hens.

Key words: chicken, rearing environment, habitat complexity, behaviour, holeboard test

Introduction

Spatial cognition is the ability of animals to acquire, use, recall and revise information concerning the spatial layout of their environment (1). It is critical for an animal's survival, as it enables navigation through the environment, locating resources such as food, water, shelter, conspecifics, and avoiding predators. Early life experiences play a considerable role in the development of spatial cognitive abilities of animals (2,3) and the complexity of the habitat in this period is a defining feature. Rearing animals in complex environments has been shown to improve spatial learning and memory (4,5), as well as having positive developmental effects on the brain regions involved in spatial cognition in a range of taxa (rat: (6), atlantic salmon: (7), mice: (8)).

The domestic hen is an excellent model species to study the development of spatial cognitive abilities due to their precocial nature (9). Precocial birds can be tested for cognitive functions from a very early age as they hatch with a fully developed sensory-motor system and rapid learning mechanisms (10). Hens are able to navigate complex environments using landmarks (11), egocentric information (12) as well as topographical features of the environment (13). They are also able to use multiple landmarks for orientation, possibly by forming cognitive maps (14). As in several other species, the development of spatial cognition in domestic chicks has also been shown to be influenced by early life environment. Chicks reared in complex environments by either equipping the housing system with elevated perches (15) or ground level visual barriers (16) have been shown to improve their spatial skills in navigating vertical spaces later in life (15) and their egocentric orientation (16). The visual barriers also induced changes in morphology of the hippocampus (17), the brain region involved in regulation of spatial navigation and memory (9).

Spatial cognition of domestic hens has been of scientific interest in applied research recently (18,19), owing to the changes in housing conditions over the last three decades (20). An increasing number of laying hens are being housed in cage-free systems such as aviaries (21),

which are structurally complex compared to cage systems (both battery and enriched) due to the vertically stacked tiers, within which essential resources such as perches, litter, food and water are located. Rearing birds in complex aviaries has been shown to benefit the spatial cognition (22) and working memory (23) of laying hens. However, the access to the three-dimensional environment is compromised during the early life period of the laying hen chicks, as they lack the physical and motor skills to access the elevated surfaces of the aviary (24–26). Producers also generally confine the chicks to the lowest aviary tier for the first two to four weeks of age (WoA), as they want the chicks to stay near food, water, and heat sources, thereby restricting the access to complex environments in their early life (27). To facilitate access to elevated surfaces in the early life of domestic chicks, studies have investigated the provision of ramps, which provide a continuous path connecting the different tiers of the aviary (28,29). Providing ramps has been shown to facilitate the chicks' access to the elevated surfaces from the first WoA in multi-tiered aviaries without any negative consequences on mortality or welfare indicators (28,29).

Facilitating early access to elevated surfaces using ramps might also have positive consequences on the development of spatial cognition of laying hens. Provision of ramps in aviaries might influence the spatial cognition of the birds mainly in two ways. First, ramps provide earlier exposure to vertical complexity. Laying hen chicks reared with ramps have been shown to use elevated surfaces earlier compared to birds reared without ramps (28,29). Early exposure to vertical complexity aided by ramps might provide earlier opportunities for spatial learning, which in turn can influence cognition and neural development. Second, ramps promote early life voluntary exercise in domestic chicks. Chicks perform more vertical movements between the tiers of aviaries when provided with ramps, with most of these movements happening through ramps (28). Ramp use is not limited to tier change; chicks also walk, run, jump to and from, and perform wing-assisted inclined running on ramps from at least three days of age (DoA) (30). The increase in early life voluntary exercise has been

shown to improve adult hippocampal neurogenesis and spatial memory in rodents (31–33), humans (34,35) and fish (36–38). Although not directly investigated in hens, it is reasonable to assume that early life voluntary exercise facilitated by ramps would positively influence the development of spatial cognition of laying hens.

Individual differences in laying hens and their importance in cognition have received a recent increase in interest (39,40). Individual hens differ in the vertical movements and use of resources including the elevated surfaces within the aviary (41,42). Consistent differences in behaviours such as exploration (43,44) and activity (41), have been established in domestic hens, which has been shown to influence the movement behaviour in various species (45,46). It is possible that these might drive differences in ramp use among individuals, potentially leading to individual developmental trajectories for spatial cognition. For example, individual hens differ in the time spent on the free range which has been shown to positively correlate with cell proliferation in the rostral hippocampal sub-region (47). Hence, it is important to account for individual differences in ramp use behaviour to better understand the impact of early life ramp use on the development of spatial cognition in laying hens.

The holeboard test has been used to assess the spatial discrimination learning abilities in various species (48), including domestic hens (23,49). For the test, a subset of all the potential sites is food-rewarded and a bird can visit any of these sites in whatever order it chooses. The holeboard test provides measures for both short and long-term spatial memories. The reference memory is an indicator of long-term memory and holds trial-independent information such as the locations of food rewards and how to access these food rewards. The working memory and general working memory are measures of short-term memory and hold information that is relevant only for a specific trial such as the sites a bird has already visited in that trial.

To the best of our knowledge, there are no studies that have looked at the relationship between individual differences in ramp use and spatial cognition of laying hens. In the current study, we planned a first exploration of the potential relationship between the early life ramp use and spatial memories of laying hen pullets using a holeboard test.

Methods

Ethical approval

The experiment met the federal and cantonal regulations for the ethical treatment of animals involved in research and was approved by the Veterinary Office of the Canton of Bern, Switzerland (BE 106/19).

Animals and Housing

Sixteen custom-built, identical pens (2.00 m × 2.00 m × 2.50 m (L × W × H)) situated at the Aviforum research facility, Zollikofen, Switzerland were used to house a total of 351 Lohmann Selected Leghorn (LSL) chicks from one day of age (DoA) until 17 weeks of age (WoA). All pens housed 22 birds, except for one pen which only had 21 chicks, due to a mistake in chick delivery Each pen was fitted with two vertically stacked tiers at heights of 0.25 m and 1.20 m from the floor, and two round metal perches (2.00 m × 0.34 cm (L × diameter)) at 0.28 m and 0.55 m above the second tier. The tiers in each pen were connected by two ramps (1.30 m × 0.24 m (L × W), at an angle of 35˚) made of metal grids (0.65 m × 0.25 m (L × W)). Both tiers were made of plastic grids with the first tier (2.00 m × 1.15 m (L × W)) being wider than the second (2.00 m × 0.60 m (L × W)). The pen floors were covered with wood shavings (2.00 m × 0.75 m (L × W)). The pens were visually isolated from each other using opaque metal sheets that covered up to 1.50 m of the pen walls from the floor. The rest was covered with plastic sheets. The schematics of the pens and more description can be

found in Johny et al. (2023). Olfactory and auditory contact was still possible between pens. Birds were populated on the first tier and confined there using wire mesh until four DoA. This was done to ensure that the birds learn the location of the drinkers and feeder. Feed was provided *ad libitum* on a feeder plate located on the first tier until two WoA, after which the feed was provided from a dispenser placed in the litter area. An additional feeder plate (40 cm diameter, 5 cm height) on the second tier was provided until five WoA. Water was provided *ad libitum* using nipple drinkers on the first tier. The birds were fed with starter feed (Egli Mühlen AG, Nebikon, Switzerland) from one until nine WoA and pullet feed (Egli Mühlen AG, Nebikon, Switzerland) from nine until 17 WoA. A light bulb (Silox basic, 120 – 3000K) attached to each pen ceiling ensured uniform illumination of 40 lux throughout the observation period. There was no provision of natural light. The light schedule followed standard rearing management for LSL (27) pullets with 24 hours of light for the first two days of life, which was gradually reduced to nine hours in the fifth WoA and stayed the same until the conclusion of the project. The dimming phase of the light lasted for five minutes at dawn and 30 minutes at dusk. Birds were vaccinated according to the standard rearing management protocol for LSL birds (Infectious bronchitis-primer, Marek's disease, Gumboro at one DoA at the hatchery, Paracox at five DoA, Infectious bronchitis 4/91 at 16 DoA, Infectious bronchitis Ma5 at 65 DoA, Avian encephalomyelitis at 93 DoA, Poulvac E.coli at 103 DoA and inactivated Infectious bronchitis vaccine at 114 DoA).

During population at one DoA, eight birds per pen were arbitrarily chosen as focal individuals. Each focal individual was marked with animal marking spray (RAIDEX, Hauptner, Switzerland) and tagged with numbered leg rings that were 5 mm in diameter (Flexi-Ringe, Fieger AG, Switzerland). We used green, black, and brown colours to mark the birds. Green and brown colours were used to mark two focal birds per colour per pen by applying a dot either on the back or neck region. Black colour was most recognizable on the chicks especially at younger ages and was used to mark four focal birds per pen. Four patterns

were used with black colour – one dot on neck or back region, one dot on both neck and back, and a continuous line that ran from the neck to back. The colour marks were reapplied at 3, 8, 15, 29 and 43 DoA. The leg rings were changed to 8, 12 and 14 mm in diameter at 8, 29 and 43 DoA, respectively. No aggression towards focal birds was observed and no difference in body mass was found between focal and non-focal birds at 14 WoA.

Birds used in the current experiment were also part of another experiment that investigated artificial cues to encourage early life ramp use in laying hen chicks (30). This experiment had four treatment groups, that investigated the use of artificial cues in which three were exposed to artificial cues while one served as a control group devoid of any cues. Because this other experiment's outcome indicated an impact of the treatment on ramp use, we mitigated the influence of treatment group-related variance by incorporating it as a control variable in the mixed-models used for statistical analyses.

Data Collection

Ramp use

Each pen was fitted with a video camera (Samsung SCO-2080R, IR, Samsung Techwin CO., Korea) connected to customized recording software (Multieye Hybrid Recorder Version 2.3.1.8, Artec Technologies AG, Diepholz, Germany) that recorded the videos at regular intervals until nine WoA. We assessed behaviours of focal birds (n = 8/pen) by counting inter-tier transitions and active behaviours on ramps, as described in Table 1 by Johny et al. (30) Inter-tier transitions were when birds moved between tiers using ramps. Active behaviours on ramps included walking, running, wing-assisted incline running (WAIR), or jumping, not resulting in a transition. We didn't differentiate between upward and downward transitions, and counted active behaviours as separate events if they were at least five seconds apart.

Both behaviours were counted from recorded videos at 6, 10, 12, 20, 27, 41, and 55 DoA for eight observation bouts of three minutes dispersed evenly across the light period (total observation time per individual per day = 24 minutes).

Holeboard test

General principles: We used the holeboard test to measure the reference, working and general working memory of the birds in three different phases following initial habituations to a food reward and the test arena. The first phase was cued and served to test the birds' ability to learn the location of three rewarded cups out of a total of eight cups with colour cues added to the rewarded cups. The second phase was uncued and involved learning the same rewarded locations as in the cued phase, but without the provision of colour cues. In the third, reversal phase, the locations of the rewarded cups were changed, and the birds had to replace the information of location of the rewarded cups they had acquired during the cued and uncued phase with a new one. There were no colour cues associated with rewarded cups in the reversal phase. The birds received 10, 20, and 10 trials each to learn the spatial configuration of the food reward for the cued, uncued, and reversal phases, respectively.

Arena and procedure: We tested three focal birds per pen (n = 48) from 56 – 95 DoA in the holeboard test. The arena design and testing methods for the holeboard test were adapted from Dumontier et al. (50). Two arenas were used to test two birds simultaneously which were situated in a separate experimental room adjacent to the barn. The arenas were enclosed by 2 m high walls made of tarpaulin on all sides and had a door on the side (Fig. 1A). Hence, birds were visually but not auditorily isolated during the testing procedure.. On the floor of each arena, eight circles distributed equally spaced in a 2 × 4 matrix were drawn with black permanent markers (Fig. 1B). In the centre of each circle, a small cup attached to a plywood platform was placed and was used to provide grape pieces as the food reward during the test. Each arena had a start box that was connected to a pulley system allowing the observer to lift

both start boxes simultaneously. Both arenas were equipped with a camera (Samsung SCO-2080R, IR, Samsung Techwin CO., Korea) connected to computer screens located outside the arena but within the experimental room allowing simultaneous viewing, recording, and coding of birds' behaviour by two researchers while birds were tested in parallel using both arenas. The researchers were trained on holeboard test videos obtained from Dumontier et al. (50) to establish inter-observer reliability before the experiment began. For the whole procedure, home pens were randomly assigned to an arena and observing researcher. Once assigned, the birds were always habituated and tested in the same arena and handled and scored by the same researcher (i.e., 8 pens or 24 birds assigned per arena and researcher). The number of grape pieces eaten and each visit of the hen to a cup were scored. If at least half the body of the hen was inside the circle and the head was oriented towards the cup, it was considered a visit.

Fig 1. Schematic representation of the holeboard arena: (1A) Two birds were tested simultaneously. The black arrows represent the direction of movement of rope and pulley system used to lift the start boxes. (1B, adapted from Dumontier et al (50). The dimensions of the arena and the general spatial configuration of the cups as viewed from above. The cups were numbered from 1 to 8. The rectangles represent the platforms of the cups.

Habituation: The focal birds were habituated to grape pieces in their home pens using the same cups as in the test. The habituation was performed over two consecutive days, with three sessions of 20 minutes per day. After habituation to the food reward, six focal birds per pen were arbitrarily chosen and habituated to the test arena in groups of three over three sessions. The first session lasted for 10 minutes and the other two was five minutes each. Each cup contained three grape pieces during the group habituation. Following the group habituation, the birds were individually habituated to the arena. Each cup contained one grape during the individual habituation. After three individual habituation sessions of five minutes each, three birds per pen that ate the most grape pieces over the three habituation sessions were selected

for the test. The selected birds were further habituated to the arena until they ate from all the cups. The holeboard test was conducted in three separate phases as follows:

Cued phase (trials 1 - 10): Three cups out of the eight cups were baited with a piece of grape. To control for the odour cues, a piece of grape was placed in a hidden compartment below the bottom of each unbaited cup. To provide cues associated with the food reward, the plywood platforms were red in colour for the baited cups, while the unbaited cups had no colour cues associated with them. Three configurations of the baited cups (i.e., the spatial position of the baited cups in the arena) were randomly chosen and assigned to the three focal birds selected for the tests from each pen (i.e., one configuration per bird). The birds were tested in a random order on each test day. At the start of each test, the birds were placed under the start boxes in both arenas by the researchers and doors of the arenas were closed. The test began when the start boxes were lifted by one of the researchers and ended when both birds had consumed the food reward from all three cups, or a maximum of five minutes had elapsed. The birds were returned to the home pen after each trial and were given 10 trials over five days to learn the configuration of the rewarded cups.

Uncued phase (trial 11 – 30): The same reward configuration and test procedure were used for both the cued and uncued phases, but the baited cups no longer had any visual cues in the latter as the plywood bases of all cups looked the same. The birds were given 20 trials over 10 days to learn the reward configuration without visual cues.

Reversal phase (trial 31 – 40): To get to the reversal phase, birds had to reach a success criterion calculated as the ratio of number of visits to baited cups to total number of visits to all cups. The ratio indicates the ability of the bird to distinguish between rewarded and unrewarded cups and can be used as a measure of learning performance (48). Birds that had reached a score of 0.6 or more in three out of four consecutive trials in the uncued phase were subjected to the reversal phase. The reversal phase lasted for 10 trials over five days and the

birds had to find the food reward in a new configuration of uncued, baited cups thus measuring the ability of the birds to unlearn the old and learn the new reward configuration. Three new reward configurations were randomly chosen and assigned to the birds that met the success criterion from each pen (i.e., one configuration per bird). Some pens had less than three birds as not all birds met the success criterion.

Data processing: For each trial, we performed the following calculations:

1. Reference memory errors: the sum of all visits and revisits to unrewarded cups. The reference memory errors indicate the ability of the birds to discriminate between rewarded and unrewarded cups.
2. Working memory errors: the revisits to rewarded cups (calculated as the difference between the number of rewarded visits and total number of visits to rewarded cups). It reflects the ability of the chickens to avoid re-visits to rewarded cups during a trial.
3. General working memory errors: total number of revisits to all the cups (calculated as the difference between the number of unique visits to rewarded and unrewarded cups and total number of visits to all cups). This indicates the ability of the chickens to avoid re-visits to already visited cups during a trial.

For all memory errors, greater values indicate a poorer performance, with zero being the best possible performance.

4. Trial duration: total time taken in seconds to consume the food rewards from all three cups. Duration was measured starting from the lift of the start box with a maximum of five minutes in case the birds failed to consume the rewards from all three rewarded cups.
5. Errors during phase change: to investigate the response of the birds to the change in phases (i.e., cued to uncued, uncued to reversal), we compared the number of memory errors of each bird from three trials before and after a phase change for all the three memory indices.

Statistical analysis

All analyses were performed with R (version 4.1.1, R Core Team, 2021) and R studio (RStudio Team, 2021) as the graphical interface. We used 'lme4' (51) package to fit linear mixed models (LMM) and package 'glmmTMB' (52) to fit generalized linear mixed effects models (GLMM). The model assumptions for homogeneity of variance and normal distribution of errors were checked using the ‘Dharma’ (53) package. When the model assumptions were not met, the data were transformed. Continuous explanatory variables were centred to zero and categorical variables were sum-contrast coded to set the reference level of contrast calculation as the mean of all groups within a variable. This was done to obtain interpretable main effects, even in the presence of interactions. The p-values of each explanatory variable were calculated by reducing each model by the particular explanatory variable and comparing it to the full model using parametric bootstrap tests (package ‘pbkrtest’ (54)). The model estimates and confidence intervals were calculated using 'broom:mixed' package (55) and the package 'emmeans' was used to calculate estimated marginal means from the full models (56). We used the framework provided by Berner and Amrhein (2022), which uses a combination of effect sizes, confidence intervals and p-values to interpret results. To reduce the numbers of tests, post-hoc comparisons were done qualitatively using numeric and visualizations of the estimated marginal means while also considering the raw data. The 'tidyverse' package (58) was used for data cleaning and the package 'ggplot2' (59) for data visualization.

Frequency of ramp use: We summed the number of transitions and active behaviours on ramps for individuals to obtain total ramp use per bird. We analysed the general ramp use behaviour of all focal birds as well as only the focal birds that underwent the holeboard test descriptively.

The association between holeboard measures and ramp use: For the cued and uncued phase, data from all focal birds that underwent the holeboard task (n = 48) and for reversal phase the

data from focal birds that met the success criteria (n = 37) were included in the analysis. The reference, working and general working memory errors, and trial duration for each bird and trial were used as response variables and were analysed separately for each phase. To relate ramp use frequency to holeboard parameters, the mean ramp use frequency (MRF) was calculated by taking mean ramp use over all observation days per focal bird and used as a continuous explanatory variable. Each model contained MRF per individual and trial number (cued: 1 - 10, uncued: 11 - 30, reversal: 31 – 40) as continuous variables as well as their interaction as explanatory variables. Configuration was added as a control variable in each model, as preliminary visualizations revealed differences in holeboard measures between different configurations. Trial number nested in bird ID nested in pen was used as random factor in all models. The models were fit with Poisson distribution for cued and reversal phase for reference memory errors, cued phase for working memory errors, and reversal phase for general working memory errors. Uncued phase of reference memory errors, cued and reversal phase of working memory errors, and cued and uncued phase of general working memory errors were fit with a negative binomial distribution. All phases of trial duration were fit with a Poisson distribution.

The effect of phase change on reference, working, and general working memory errors were analysed separately for both phase changes. The trials and birds for the phase change analysis were chosen as per point five under the 'data processing' section. All models included an interaction of MRF and respective phases (for ex., cued and uncued for cued to uncued phase change analysis) as categorical explanatory variables. Reward configuration was added as a control variable and trial number nested in bird ID nested in pen was included as a random factor in all models.

The data and code for all analysis can be found at https://osf.io/9j2g8

Results

Ramp use

The mean individual ramp use over 56 observations (eight bouts per seven observation days) was 0.39 uses of ramps per observation bout (i.e., per three minutes) for all focal birds (n = 128). The mean individual ramp use per bout ranged from 0.09 to 1.00 with a standard deviation of 0.16. Of the 7,186 observations, 79% of the observations were zeroes (5,698).

The mean individual ramp use of focal birds (n = 48) that underwent the holeboard test was 0.38 uses of ramps (range 0.09 – 0.67) per observation bout with a standard deviation of 0.14. Eighty percent (2,153 observations) of the total 2,688 observations were zeros.

Holeboard test

Reference memory errors: Birds with higher MRF demonstrated a decrease in errors compared to birds with lower MRF during the cued phase, although statistical evidence was limited (model estimates (Est) ± CI = 0.94 [0.88, 0.99], p = 0.08, Fig. 2). For example, the bird with the lowest MRF made slightly more errors [Estimated marginal means (EMM) ± CI = 3.36 [2.52, 4.48]) than the ones with median (2.95 [2.61, 3.35]) and highest (2.55 [1.91, 3.41]) MRF. The number of errors also decreased with trial number (Est ± CI = 0.75 [0.76, 0.85], p = 0.001) in the cued phase. The interaction of MRF and trial number did not influence the number of errors made in the cued phase (Est ± CI = 0.97 [0.32, 0.92], p = 0.31). In the uncued phase, the number of errors reduced with trial number (Est ± CI = 0.93 [0.89, 1.07], p = 0.001), but there were no influences of MRF (Est ± CI = 1.01 [0.96, 1.07], p = 0.74) or the interaction between MRF and trial number (Est ± CI = 1.00 [0.97, 1.04], p = 0.98). In the reversal phase, the birds with higher MRF made more errors than the birds with lower MRF (Est ± CI = 1.10 [1.01, 1.20], p = 0.05, Fig. 3). The bird with the lowest MRF made slightly fewer errors (EMM ± CI = 2.72 [1.82, 4.07]) than the ones with median (3.30 [2.80, 3.90] and high (4.01 [2.78, 5.77]) MRF. With limited statistical support, the number of errors

slightly decreased with trial number (Est ± CI = 0.95 [0.9, 1.01], p = 0.08) in the reversal phase. The interaction of MRF and trial number did not have any effect on the number of errors made in the reversal phases (Est ± CI = 0.99 [0.94, 1.04], p = 0.82).

Fig 2: The relationship between reference memory errors (sum of all visits and revisits to unrewarded cups) and mean individual ramp use frequency in the cued phase (model estimates ± CI = 0.94 [0.88, 0.99], p = 0.08). The black points connected by the black line represent the estimated marginal means and the shaded ribbon represents 95 % confidence intervals. The coloured points represent reference memory errors (all visits – visits to rewarded cups) of focal birds' colour-graded according to mean ramp use frequency.

Fig 3: The relationship between reference memory errors (sum of all visits and revisits to unrewarded cups) and mean individual ramp use frequency in the reversal phase (model estimates ± CI = 1.10 [1.01, 1.20], p = 0.05). The black points connected by the black line represent the estimated marginal means and the shaded ribbon represents 95 % confidence intervals. The coloured points represent reference memory errors (all visits – visits to rewarded cups) of focal birds, colour-graded according to mean ramp use frequency.

Working memory: In general, all birds made very few working memory errors throughout all phases (Mean ± SD: cued – 0.33 ± 0.69, uncued – 0.23 ± 0.64, reversal – 0.34 ± 0.74). With weak statistical support, the birds with higher MRF made marginally fewer errors than birds with a lower MRF (Est ± CI = 0.77 [0.59, 1.00], p = 0.06, Fig. 4) in the cued phase. The bird with the lowest MRF (EMM ± CI = 0.47 [0.15, 1.45]) made slightly more errors than the ones with median (EMM ± CI = 0.27 [0.15, 0.47]) and the one with the highest MRF (EMM ± CI = 0.15 [0.05, 0.52]). There was a slight reduction in the number of errors as the trial number increased, although the statistical support for this find was weak (Est ± CI = 0.84 [0.69, 1.03], p = 0.08). The interaction between trial number and MRF did not a have an effect number of working memory errors in the cued phase (Est ± CI = 0.94 [0.78, 1.15], p = 0.80). In the

uncued phase, the number of errors decreased with trial number (Est ± CI = 0.87 [0.76, 0.99], p = 0.05). No influence of MRF (Est ± CI = 1.07 [0.86, 1.39], p = 0.12) or the interaction between MRF and trial number (Est ± CI = 0.95 [0.84, 1.08], p = 0.45) was found on the number of errors made in the uncued phase. In the reversal phase, the number of errors made by the birds was not influenced by MRF (Est ± CI = 1.03 [0.78, 1.36], p = 0.87), trial number (Est ± CI = 0.83 [0.67, 1.06], p = 0.14) or their interaction (Est ± CI = 0.84 [0.66, 1.05], p = 0.14).

Fig 4: The relationship between working memory errors (sum of all revisits to rewarded cups) and mean individual ramp use frequency in the cued phase (model estimates ± CI = 0.77 [0.59, 1.00], p = 0.06). The black points connected by the black line represent the estimated marginal means and the shaded ribbon represents 95 % confidence intervals. The coloured points represent working memory errors (all visits to rewarded cups – rewarded visits) of focal birds, colour-graded according to mean ramp use frequency.

General working memory: Birds made very few general working memory errors in all three phases (Mean ± SD: cued – 0.73 ± 1.56, uncued – 0.62 ± 1.55, reversal – 0.86 ± 1.61). The number of errors made by birds decreased with trial number with varying statistical support for each phase: cued (Est ± CI = 0.69 [0.56, 0.84], p = 0.003), uncued (Est ± CI = 0.85 [0.72, 1.04], p= 0.08) and reversal (Est ± CI = 0.87 [0.78, 0.97], p = 0.02). No influences of MRF (Est ± CI, cued = 0.80 [0.61, 1.04], p = 0.12, uncued = 1.10 [0.86, 1.39], p = 0.46, reversal = 1.17 [0.90, 1.53], p = 0.30) and the interaction between MRF and trial number (Est ± CI, cued = 0.89 [0.70, 1.10], p = 0.28, uncued = 0.99 [0.83, 1.19], p = 0.93, reversal = 0.94 [0.84, 1.06], p = 0.30) were found for any of the phases.

Trial duration: The trials in which the birds failed to consume from all baited cups were removed from the analysis (9, 4 and 1 out of 480, 960 and 370 observations from cued, uncued and reversal phases, respectively). Trial duration was influenced by an interaction

between MRF and trial number (Est ± CI = 0.88 [0.84, 0.93], p = 0.001) in the cued phase, with birds with a high MRF showing a faster decline in trial duration with increasing trial number (Estimated slopes ± CI for selected MRF: lowest = - 0.19 [- 0.24, - 0.13], median = - 0.29[- 0.32, -0.27], highest = - 0.40 [- 0.45, −0.34]). Trial duration decreased with increasing trial number in the uncued (Est ± CI = 0.99 [0.98, 1.00], p = 0.001) and reversal (Est ± CI = 0.93 [0.91, 0.95], p = 0.001) phases. No effect of MRF (uncued = 1.05 [0.53, 2.02], p = 0.33, reversal = 0.18 [0.02, 2.03], p = 0.20) nor the interaction of MRF and trial number (uncued = 1.00 [0.99, 1.02], p = 0.78, reversal = 1.07 [1.01, 2.03], p = 0.79) were found for trial duration for the uncued and reversal phases.

Phase change: The interaction between phase change and MRF) had an effect on the reference memory errors (p = 0.001) for the cued to uncued phase change with birds with a higher MRF making more errors than the birds with a lower MRF as the phase changed from cued to uncued (Fig 5A). For the uncued to reversal phase change, there was no effect of the interaction between phase change and MRF on the reference errors (p = 0.17). The interaction between phase change and ramp use group had no effect on the working memory scores for both phase changes (cued to uncued, p = 0.21, uncued to reversal, p = 0.32). For the general working memory, the interaction between phase change and ramp use group showed an effect for the cued to uncued (p = 0.02), but not for the uncued to reversal phase change (p = 0.16). The birds with the lower ramp use made marginally more errors than the ones with higher ramp use as the phase changed from cued to uncued for the general working memory (Fig 5B).

Fig 5: The relationship between ramp use frequency and spatial memory errors (A) Reference memory, B) General working memory) as the phase changed from cued to uncued phase. The point ranges represent the estimated marginal means of reference memory errors and the points are the raw data, colour graded according to mean ramp use frequency.

Discussion

The aim of the study was to perform a first exploration into the relationship between early life ramp use (1-8 WoA) and short and long-term spatial memory in laying hen pullets. We found that the birds that used ramps more made fewer reference and working memory errors in the cued phase than birds with lower ramp use. However, birds with higher ramp use made more reference memory errors in the reversal phase. The birds that used ramps more often also showed a faster decline in time taken to solve the holeboard test with increasing trial numbers in the cued phase. For the phase change analysis, we found that birds with a higher ramp use made more reference and general working memory errors in the cued to uncued phase change than the birds that used ramps less often. Our results indicate a relationship between early life ramp use and spatial memories in laying hens, which can have several explanations.

The relationship between early life ramp use and spatial memories

One possible explanation for the association between holeboard measures and ramp use might be that the individual differences in both ramp use and cognition are driven by consistent inter-individual differences in behaviour. Consistent differences in behaviours such as boldness, exploration and activity have been shown to correlate to movement in animals (46,60). For example, bolder bank voles (61) and Trinidad killifish (62) move longer distances, fast exploring female great tits disperse to a greater distance (63), and high exploratory laying hens use the outdoor range more than the ones that are less exploratory (64,65). It is possible that individual differences in ramp use might also be driven by individual differences in activity, exploration, and fearfulness, with more active, explorative, and less fearful birds showing more use of ramps.

The speed-accuracy trade-off, proposed by Sih and Del Giudice (66), is a key framework for understanding consistent inter-individual differences in behaviour and cognition. It suggests that fast behavioural types (with high exploration and activity levels, neophilia, etc.) prioritize speed over accuracy, acquiring simple rules quickly but making more mistakes in challenging situations like reversal learning. Conversely, slow behavioural types (with low exploration and activity levels, neophobia, etc.) prioritize accuracy, and while they are slower at simple learning tasks, they are more sensitive to changes in their environment and make fewer errors during reversal tests. While not studied in domestic hens, our results seem to align with this model. Birds with higher ramp use made fewer spatial memory errors and learned tasks faster in the cued phase, suggesting better associative learning (67). In contrast, birds with lower ramp use made fewer errors during the reversal phase, indicating higher behavioural flexibility (68). They also showed fewer memory errors during phase change from cued to uncued, suggesting an ability to update learning strategies with environmental shifts. Additionally, the marginally higher working memory errors of birds with low ramp use in the cued phase may indicate neophobia, as cues were absent during habituation.

Empirical research however indicates that the relationship between consistent inter-individual differences in behaviour and cognition does not always follow the speed-accuracy trade-off framework and that the relationship is dependent on factors such as species, sex, and age (69). For example, Zidar et al. (44) showed a complex and age-dependent relationship between cognition and behavioural type in red jungle fowl. As red jungle fowls and domestic chicks are closely related, we may need further studies across multiple ages to elucidate the complex relationship between consistent inter-individual differences in behaviour and cognition in laying hens.

Another possible explanation of our results might be that early life ramp use did not have a meaningful influence on the spatial memories of the hens, a position supported by the low

effect sizes (or influence) of MRF on spatial memories (Reference memory, cued phase: 0.94, reversal phase: 1.10). Moreover, we also didn't see any differences in the spatial memories in the uncued phase, which would be expected if the birds with different ramp use varied in their spatial cognition as well. As described within the context of the speed-accuracy trade off above, the differences in spatial memories observed in the cued and reversal phase can be explained by a difference in associative learning and behavioural flexibility, respectively. In comparison to the cued and reversal phases, the performance in the uncued phase is directly related to spatial memories and has been shown to be controlled by the hippocampus (48), the region that plays a central role in processing spatial information (9). Hence the lack of differences in spatial memories in the uncued phase, combined with low effect sizes of reference memories in the cued and reversal phase suggests that early life ramp use might only have a minor impact on the spatial cognition of the pullets.

Evaluation of holeboard task

Unlike in rodents and pigs, the holeboard task is not widely used to measure the spatial cognition of hens and hence require a through consideration of the variations and limitations of the methods. Our holeboard test setup was similar to Dumontier et al. (50), except for the lack of two start box positions in the arena and the order of the phases as it was unuced, cued, overtraining and reversal phase for Dumontier et al. (50). Also, instead of including an overtraining phase as used by Dumontier et al. (50), we used a success criteria to make sure the birds had learnt the spatial location of the rewards before continuing with the reversal phase. We calculated memory errors rather than memory ratios like Dumontier et al. (50), because of the low variation in the working memory and general working memory ratios in our study. Our birds showed relatively high working (mean ± SD = 0.94 ± 0.13) and general working memory scores (0.93 ± 13) which were similar to Dumontier et al. (50) (working memory = 0.8 – 0.9, general working memory = 0.7 – 0.9), but slightly better than in more

complex holeboard tests with a 3 × 3 matrix of cups (working memory = 0.7 – 0.8 (49) and 0.6 – 0.9 in (23)). The reference memory in our study was 0.55 ± 0.13, which again was similar to other studies (0.4 – 0.7; (23,44,45)). The similarity in scores with other studies, coupled with the decrease in reference memory errors throughout all phases suggest that the birds did learn the task.

As noted by Dumontier et al. (50) and Tahamtani et al. (23), one general criticism of the holeboard test as a method might be the low cost to check the unrewarded cups. There was only a marginal decrease in reference memory errors in the uncued phase from trial 11 to 30. The reference memory errors also showed a large variation across consecutive trials, which might be due to birds checking the all the cups in certain trials, as it incurs only a negligible energy expenditure. If indeed the birds were checking all the cups in certain trials, the lack of large differences in references memory errors observed in our study might not be indicative of lack differences in spatial memory. Future studies employing the holeboard test might benefit from adding additional costs, such as bigger arenas with higher proportion of unrewarded holes or birds required to manipulate the hole, to obtain rewards. For instance, holeboard tests for pigs employ a ball to cover the food bowl, which the pigs have to manipulate using their snout to gain access to rewards underneath (70).

Another limitation specific to our study is the use of one start location throughout the test, which can result in egocentric approaches such as fixed food search patterns (48). In addition, the rectangular arena with a 2×4 matrix distribution of rewards, can lead to fixed simple food search patterns such as visiting the closest cup straight ahead.

Outlook

The current study contributes to the existing body of research exploring the relationship between early-life habitat complexity and spatial cognition in laying hens. Many studies have

covered multiple levels of complexity (cage vs aviary (23,50), floor vs three-dimensional rearing (15,71)) and ages for a set of spatial cognitive indices (spatial long and short-term memories (23,50), depth perception (71), neuro-motor coordination (15)). Nonetheless, the long and short-term impacts of early life habitat complexity on the spatial cognition of laying hens remains ambiguous. Future studies should account for effect of age, type of test used and include additional measures of brain development such as neural cell counts to better understand the impact early life habitat complexity has on the development of spatial cognition in laying hens. Furthermore, it is important to investigate the specific level of habitat complexity required to induce developmental changes in the spatial cognitive abilities of the birds. This aspect deserves further investigation to determine the optimal conditions for promoting spatial cognition in laying hens.

Acknowledgements

We thank Abdulsatar Abdel Rahman for helping with the holeboard task. Many thanks to Thomas Heinzel and Jan Büchler, for building the pens and arena, Masha Marincek and Josie Siegel for their help with the video analysis. We thank Lucille Dumontier for the discussions on the statistical analysis. We thank the Ana Rentsch for her valuable feedback that improved the quality of the manuscript.

References

1. Thinus-Blanc C. Animal Spatial Cognition. Anim Spat Cogn. 1996 Nov;

2. De Pasquale C, Neuberger T, Hirrlinger AM, Braithwaite VA. The influence of complex and threatening environments in early life on brain size and behaviour. Proc R Soc B Biol Sci. 2016 Jan 27;283(1823).

3. Buchanan KL, Grindstaff JL, Pravosudov V V. Condition dependence, developmental

plasticity, and cognition: implications for ecology and evolution. Trends Ecol Evol. 2013 May 1;28(5):290–6.

4. Salvanes AGV, Moberg O, Ebbesson LOE, Nilsen TO, Jensen KH, Braithwaite VA. Environmental enrichment promotes neural plasticity and cognitive ability in fish. Proc R Soc B Biol Sci. 2013 Sep 22;280(1767).

5. Whiteside MA, Sage R, Madden JR. Multiple behavioural, morphological and cognitive developmental changes arise from a single alteration to early life spatial environment, resulting in fitness consequences for released pheasants. R Soc Open Sci. 2016 Mar 23;3(3).

6. Falkenberg T, Mohammed AK, Henriksson B, Persson H, Winblad B, Lindefors N. Increased expression of brain-derived neurotrophic factor mRNA in rat hippocampus is associated with improved spatial memory and enriched environment. Neurosci Lett. 1992 Apr 13;138(1):153–6.

7. Mes D, Van Os R, Gorissen M, Ebbesson LOE, Finstad B, Mayer I, et al. Effects of environmental enrichment on forebrain neural plasticity and survival success of stocked Atlantic salmon. J Exp Biol. 2019 Dec 1;22(23).

8. van Praag H, Kempermann G, Gage FH. Neural consequences of enviromental enrichment. Nat Rev Neurosci 2000 13. 2000;1(3):191–8.

9. Morandi-Raikova A, Mayer U. Spatial cognition and the avian hippocampus: Research in domestic chicks. Front Psychol. 2022 Sep 23;13:5791.

10. Rosa-Salva O, Mayer U, Versace E, Hébert M, Lemaire BS, Vallortigara G. Sensitive periods for social development: Interactions between predisposed and learned mechanisms. Cognition. 2021 Aug 1;213:104552.

11. Della Chiesa A, Pecchia T, Tommasi L, Vallortigara G. Multiple landmarks, the encoding of environmental geometry and the spatial logics of a dual brain. Anim Cogn. 2006 Oct 29;9(4):281–93.

12. Vallortigara G. Learning of colour and position cues in domestic chicks: Males are better at position, females at colour. Behav Processes. 1996 Jun 1;36(3):289–96.

13. Rashid N, Andrew RJ. Right hemisphere advantage for topographical orientation in the domestic chick. Neuropsychologia. 1989 Jan 1;27(7):937–48.

14. Morandi-Raikova A, Vallortigara G, Mayer U. The use of spatial and local cues for orientation in domestic chicks (Gallus gallus). Anim Cogn 2020 232. 2020 Jan 1;23(2):367–87.

15. Gunnarsson S, Yngvesson J, Keeling LJ, Forkman B. Rearing without early access to perches impairs the spatial skills of laying hens. Appl Anim Behav Sci. 2000 Apr 3;67(3):217–28.

16. Freire R, Cheng HW, Nicol CJ. Development of spatial memory in occlusion-experienced domestic chicks. Anim Behav. 2004 Jan 1;67(1):141–50.

17. Freire R, Cheng HW. Experience-dependent changes in the hippocampus of domestic chicks: a model for spatial memory. Eur J Neurosci. 2004 Aug 1;20(4):1065–8.

18. Ferreira VHB, Guesdon V, Calandreau L. How can the research on chicken cognition improve chicken welfare: a perspective review. https://doi.org/101080/0043933920211924920. 2021;77(3):679–98.

19. Widowski T, Torrey S. Rearing young birds for adaptability. Adv Poult Welf. 2018 Jan 1;49–76.

20. Leenstra F, Ten Napel J, Visscher J, Van Sambeek F. Layer breeding programmes in

changing production environments: a historic perspective. Worlds Poult Sci J. 2016 Mar 1;72(1):21–36.

21. Schuck-Paim C, Negro-Calduch E, Alonso WJ. Laying hen mortality in different indoor housing systems: a meta-analysis of data from commercial farms in 16 countries. Sci Reports 2021 111. 2021 Feb 4;11(1):1–13.

22. Rentsch AK, Harlander A, Niel L, Siegford JM, Widowski TM. Rearing laying hens: Environmental complexity and genetic strain affect pullet but not chick performance in a T-maze learning task. Appl Anim Behav Sci. 2023;265:105997.

23. Tahamtani FM, Nordgreen J, Nordquist RE, Janczak AM. Early life in a barren environment adversely affects spatial cognition in laying hens (Gallus gallus domesticus). Front Vet Sci. 2015 Mar 18;2(MAR).

24. Muir GD, Gosline JM, Steeves JD. Ontogeny of bipedal locomotion: walking and running in the chick. J Physiol. 1996 Jun 1;493(2):589–601.

25. Muir GD. Early ontogeny of locomotor behaviour: a comparison between altricial and precocial animals. Brain Res Bull. 2000 Nov 15;53(5):719–26.

26. Rentsch AK, Ross E, Harlander A, Niel L, Siegford JM, Widowski TM. The development of laying hen locomotion in 3D space is affected by early environmental complexity and genetic strain. Sci Rep. 2023;13(1):10084.

27. Thiele H-H. Management recommendations for rearing pullets for alternative housing systems. Lohmann Inf. 2007;42:14–24.

28. Stratmann A, Guggisberg D, Benavides-Reyes C, Siegford J, Toscano MJ. Providing ramps in rearing aviaries affects laying pullet distribution, behavior and bone properties. J Appl Poult Res. 2022 Dec 1;31(4):100283.

29. Norman KI, Weeks CA, Tarlton JF, Nicol CJ. Rearing experience with ramps improves specific learning and behaviour and welfare on a commercial laying farm. Sci Rep. 2021 Apr 23;11(1):1–14.

30. Johny A, Guggisberg D, Toscano MJ, Stratmann A. This is the way: The effect of artificial cues on early life ramp use behaviour of laying hen chicks. Appl Anim Behav Sci. 2023 Mar 1;260:105873.

31. Van Praag H, Christie BR, Sejnowski TJ, Gage FH. Running enhances neurogenesis, learning, and long-term potentiation in mice. Proc Natl Acad Sci U S A. 1999 Nov 9;96(23):13427–31.

32. Van Praag H. Neurogenesis and exercise: Past and future directions. NeuroMolecular Med. 2008 Jun 20;10(2):128–40.

33. Merkley CM, Jian C, Mosa A, Tan YF, Wojtowicz MM. Homeostatic regulation of adult hippocampal neurogenesis in aging rats: long-term effects of early exercise. Front Neurosci. 2014;8(8 JUL).

34. Hillman CH, Erickson KI, Kramer AF. Be smart, exercise your heart: exercise effects on brain and cognition. Nat Rev Neurosci 2008 91. 2008 Jan;9(1):58–65.

35. Sibley BA, Etnier JL. The Relationship between Physical Activity and Cognition in Children: A Meta-Analysis. Pediatr Exerc Sci. 2003 Aug 1;15(3):243–56.

36. Rossi GS, Wright PA. Does leaving water make fish smarter? Terrestrial exposure and exercise improve spatial learning in an amphibious fish. Proc R Soc B. 2021 Jun 30;288(1953).

37. Mes D, Palstra AP, Henkel C V., Mayer I, Vindas MA. Swimming exercise enhances brain plasticity in fish. R Soc Open Sci. 2020 Jan 1;7(1).

38. Fiaz AW, Léon-Kloosterziel KM, Leeuwen JL van, Kranenbarg S. Exploring the effect of exercise on the transcriptome of zebrafish larvae (Danio rerio). J Appl Ichthyol. 2014 Aug 1;30(4):728–39.

39. Boogert NJ, Madden JR, Morand-Ferron J, Thornton A. Measuring and understanding individual differences in cognition. Philos Trans R Soc B Biol Sci. 2018 Sep 26;373(1756).

40. Shaw RC, Schmelz M. Cognitive test batteries in animal cognition research: evaluating the past, present and future of comparative psychometrics. Anim Cogn. 2017 Nov 1;20(6):1003–18.

41. Rufener C, Berezowski J, Maximiano Sousa F, Abreu Y, Asher L, Toscano MJ. Finding hens in a haystack: Consistency of movement patterns within and across individual laying hens maintained in large groups. Sci Rep. 2018 Dec 1;8(1).

42. Gómez Y, Berezowski J, Jorge YA, Gebhardt-Henrich SG, Vögeli S, Stratmann A, et al. Similarity in Temporal Movement Patterns in Laying Hens Increases with Time and Social Association. Animals. 2022 Mar 1;12(5):555.

43. de Haas EN, Lee C, Hernandez CE, Naguib M, Rodenburg TB. Individual differences in personality in laying hens are related to learning a colour cue association. Behav Processes. 2017 Jan 1;134:37–42.

44. Zidar J, Balogh A, Favati A, Jensen P, Leimar O, Sorato E, et al. The relationship between learning speed and personality is age- and task-dependent in red junglefowl. Behav Ecol Sociobiol. 2018 Oct 1;72(10):1–13.

45. Nilsson J-Å, Brönmark C, Hansson L-A, Chapman BB. Individuality in movement: the role of animal personality. Anim Mov across scales. 2014;1:90–109.

46. Spiegel O, Leu ST, Bull CM, Sih A. What's your move? Movement as a link between personality and spatial dynamics in animal populations. Ecol Lett. 2017 Jan 1;20(1):3–18.

47. Armstrong EA, Voelkl B, Voegeli S, Gebhardt-Henrich SG, Guy JH, Sandilands V, et al. Cell Proliferation in the Adult Chicken Hippocampus Correlates With Individual Differences in Time Spent in Outdoor Areas and Tonic Immobility. Front Vet Sci. 2020 Aug 26;7:587.

48. van der Staay FJ, Gieling ET, Pinzón NE, Nordquist RE, Ohl F. The appetitively motivated "cognitive" holeboard: A family of complex spatial discrimination tasks for assessing learning and memory. Neurosci Biobehav Rev. 2012 Jan 1;36(1):379–403.

49. Nordquist RE, Heerkens JLT, Rodenburg TB, Boks S, Ellen ED, van der Staay FJ. Laying hens selected for low mortality: Behaviour in tests of fearfulness, anxiety and cognition. Appl Anim Behav Sci. 2011 May 1;131(3–4):110–22.

50. Dumontier L, Janczak AM, Smulders T V., Nordgreen J. Effects of the rearing environment complexity on laying hens' spatial cognition: A holeboard test approach. Appl Anim Behav Sci. 2023 Mar 1;260:105878.

51. Bates D, Mächler M, Bolker BM, Walker SC. Fitting Linear Mixed-Effects Models Using lme4. J Stat Softw. 2015 Oct 7;67(1):1–48.

52. Brooks ME, Kristensen K, van Benthem KJ, Magnusson A, Berg CW, Nielsen A, et al. glmmTMB balances speed and flexibility among packages for zero-inflated generalized linear mixed modeling. R J. 2017 Dec 1;9(2):378–400.

53. Hartig F. DHARMa: Residual Diagnostics for Hierarchical (Multi-Level / Mixed) Regression Models. R package version 0.2.0. https://CRANR-

project.org/package=DHARMa. 2020;

54. Halekoh U, Højsgaard S. A Kenward-Roger Approximation and Parametric Bootstrap Methods for Tests in Linear Mixed Models – The R Package pbkrtest. J Stat Softw. 2014 Sep 12;59(9):1–32.

55. Bolker B, Robinson D. Broom. mixed: Tidying methods for mixed models. R Packag version 02. 2020;6.

56. Lenth R, Singmann H, Love J, Buerkner P, Herve M. Package 'emmeans'. 2019.

57. Berner D, Amrhein V. Why and how we should join the shift from significance testing to estimation. J Evol Biol. 2022;35(6):777–87.

58. Wickham H, Averick M, Bryan J, Chang W, D' L, Mcgowan A, et al. Welcome to the Tidyverse. J Open Source Softw. 2019 Nov 21;4(43):1686.

59. Wickham H, Chang W. Package ggplot2: An Implementation of the Grammar of Graphics. Creat elegant data Vis using Gramm Graph. 2016;v. 2(1):1–189.

60. Shaw AK. Causes and consequences of individual variation in animal movement. Mov Ecol 2020 81. 2020 Feb 17;8(1):1–12.

61. Schirmer A, Herde A, Eccard JA, Dammhahn M. Individuals in space: personality-dependent space use, movement and microhabitat use facilitate individual spatial niche specialization. Oecologia. 2019;189:647–60.

62. Fraser DF, Gilliam JF, Daley MJ, Le AN, Skalski GT. Explaining Leptokurtic Movement Distributions: Intrapopulation Variation in Boldness and Exploration. Am Nat. 2001;158(2):124–35.

63. Dingemanse NJ, Both C, Van Noordwijk AJ, Rutten AL, Drent PJ. Natal dispersal and

personalities in great tits (Parus major). Proc R Soc London Ser B Biol Sci. 2003;270(1516):741–7.

64. Kolakshyapati M, Taylor PS, Hamlin A, Sibanda TZ, Vilela J de S, Ruhnke I. Frequent Visits to an Outdoor Range and Lower Areas of an Aviary System Is Related to Curiosity in Commercial Free-Range Laying Hens. Anim 2020, Vol 10, Page 1706. 2020 Sep 21;10(9):1706.

65. Campbell DLM, Hinch GN, Downing JA, Lee C. Fear and coping styles of outdoor-preferring, moderate-outdoor and indoor-preferring free-range laying hens. Appl Anim Behav Sci. 2016 Dec 1;185:73–7.

66. Sih A, Del Giudice M. Linking behavioural syndromes and cognition: a behavioural ecology perspective. Philos Trans R Soc B Biol Sci. 2012;367(1603):2762–72.

67. Pearce JM, Bouton ME. Theories of Associative Learning in Animals. https://doi.org/101146/annurev.psych521111. 2003 Nov 28;52:111–39.

68. Rajalakshmi R, Jeeves MA. The relative difficulty of reversal learning (reversal index) as a basis of behavioural comparisons. Anim Behav. 1965 Apr 1;13(2–3):203–11.

69. Dougherty LR, Guillette LM. Linking personality and cognition: a meta-analysis. Philos Trans R Soc B Biol Sci. 2018 Sep 26;373(1756).

70. Roelofs S, Murphy E, Ni H, Gieling E, Nordquist RE, van der Staay FJ. Judgement bias in pigs is independent of performance in a spatial holeboard task and conditional discrimination learning. Anim Cogn. 2017;20:739–53.

71. Jones CT, Pullin AN, Blatchford RA, Makagon MM, Horback K. Effects of rearing with vertical structures on the ontogeny of depth perception in laying hens. Appl Anim Behav Sci. 2023 Feb 1;259:105837.

Figure_1

Click here to access/download;Figure;fig_1.tif

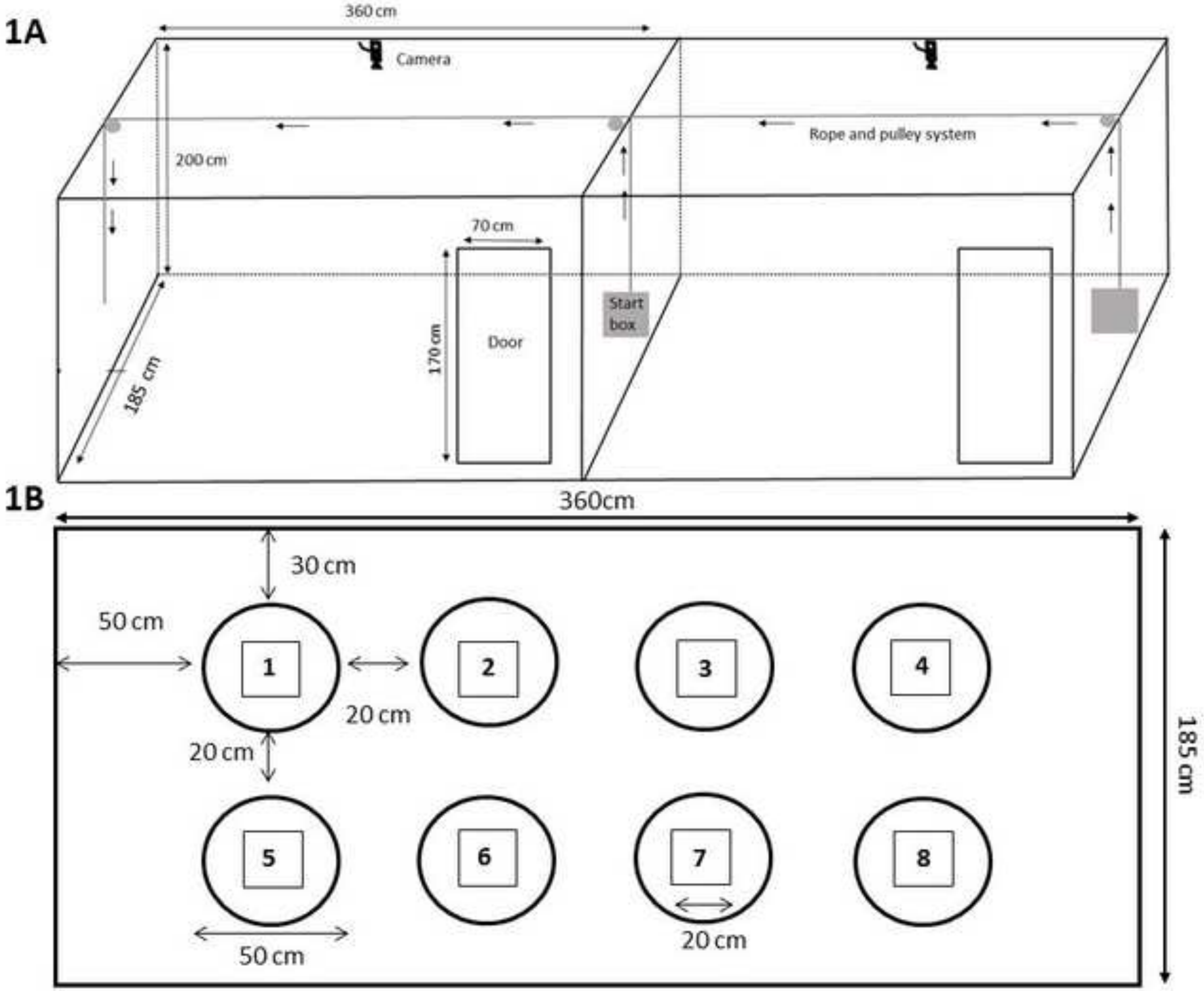

Figure 2

Click here to access/download;Figure;fig_2.tif

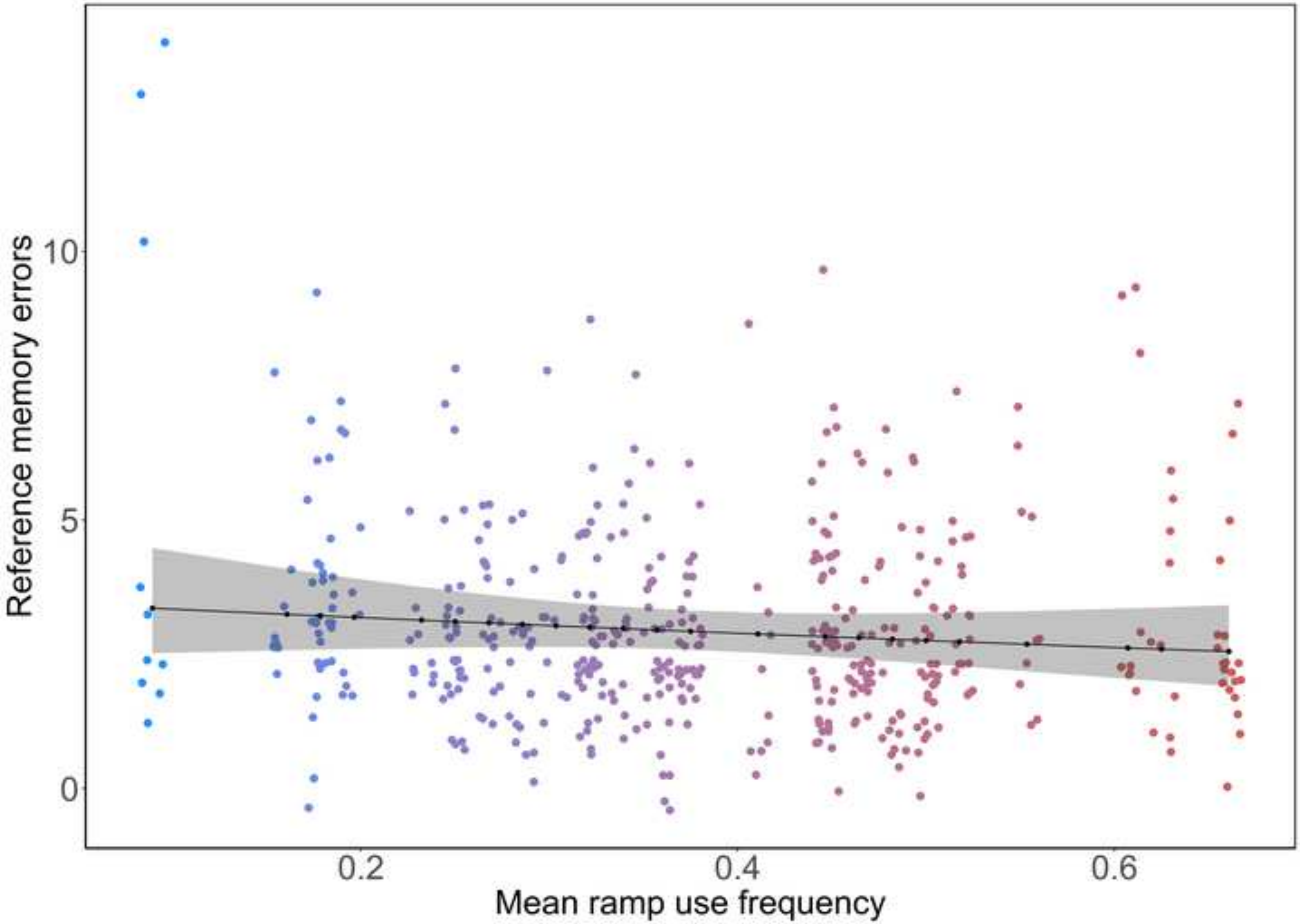

Figure 3

Click here to access/download;Figure;fig_3.tif

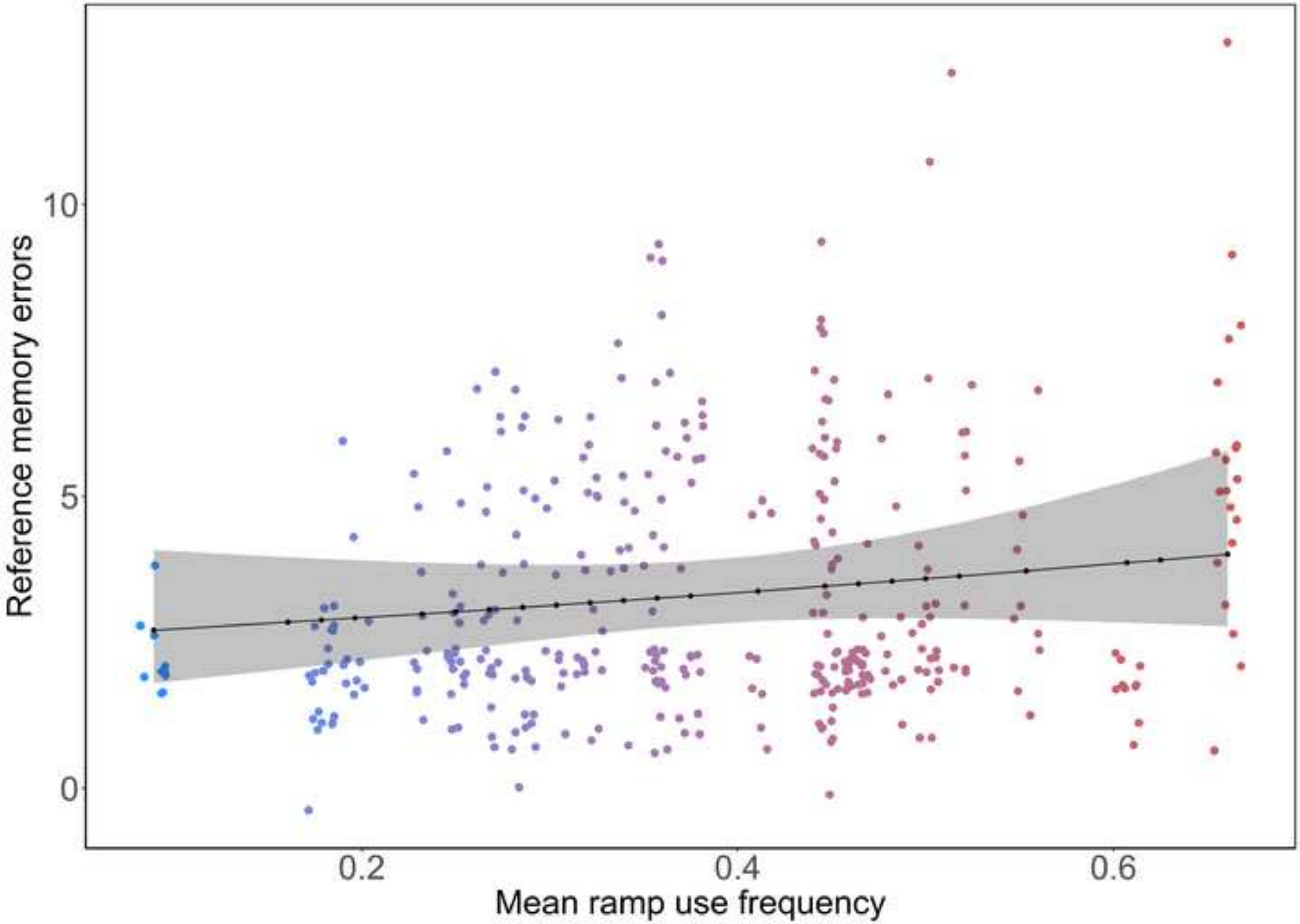

Figure 4

Click here to access/download;Figure;fig_4.tif

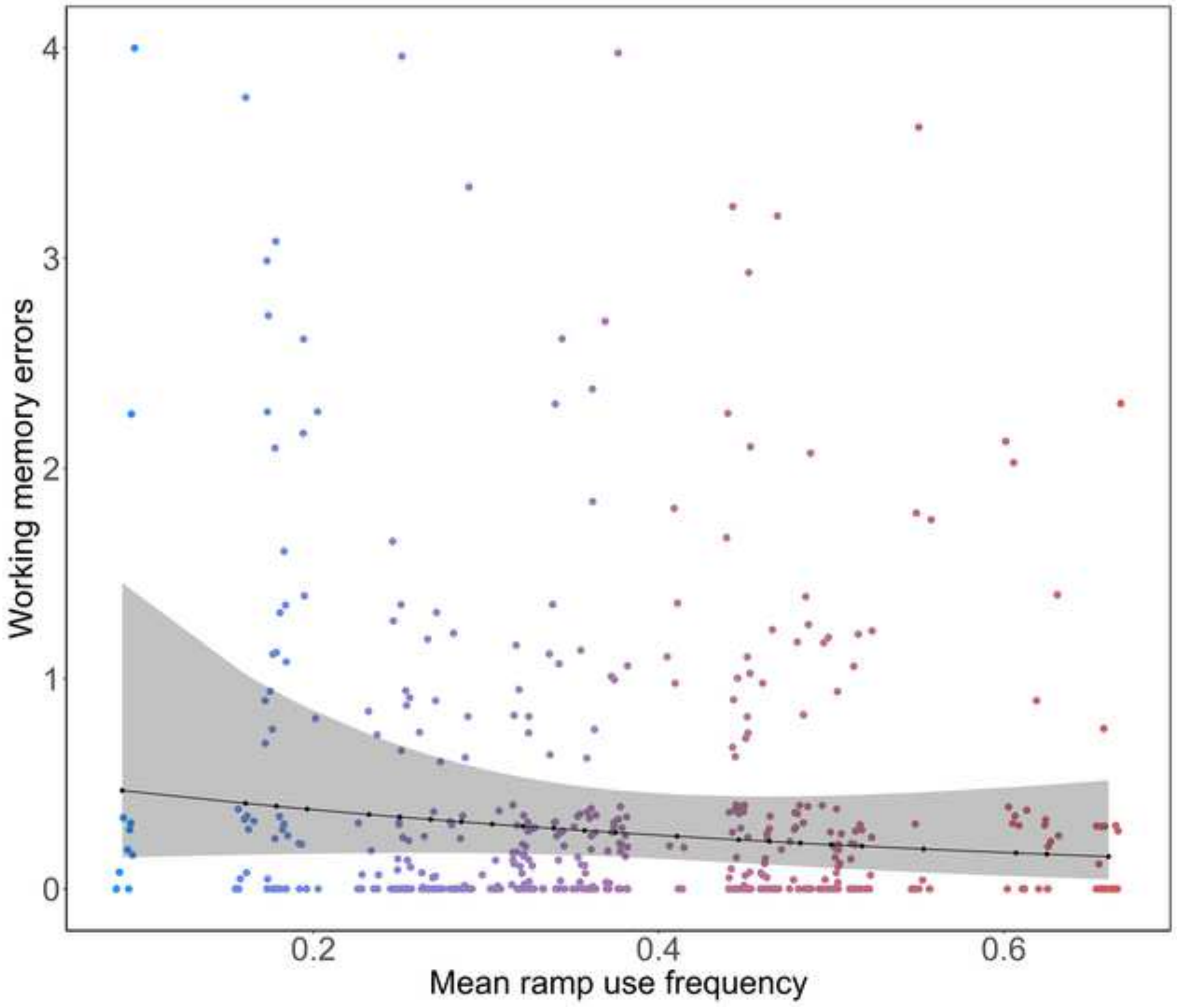

Figure 5

Click here to access/download;Figure;fig_5.tif

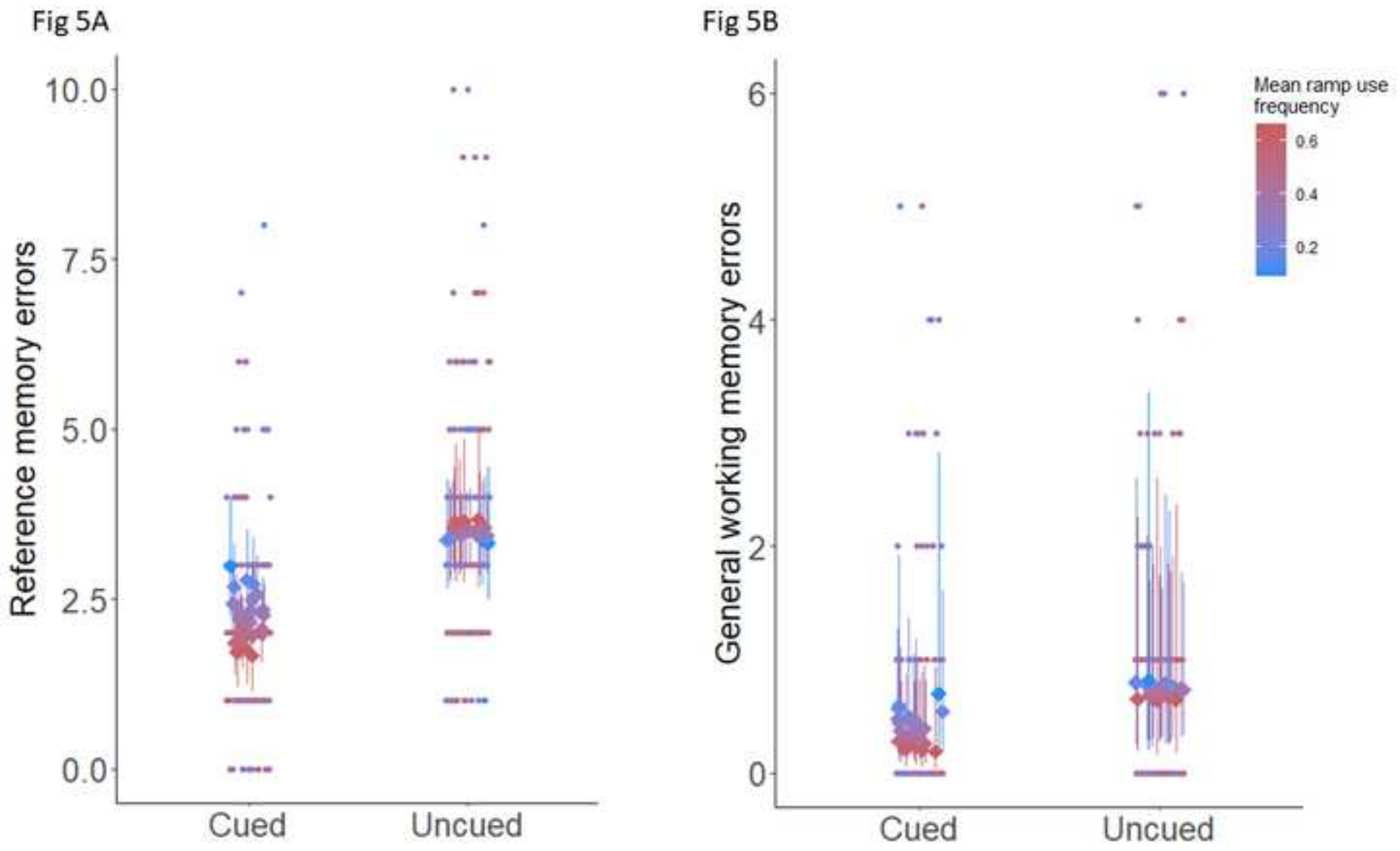

Mind the ramp: Association between early life ramp use and spatial cognition in laying hen pullets.

Alex Johny[1, 2*], Andrew M. Janczak[3], Janicke Nordgreen[4], Michael J. Toscano[1], Ariane Stratmann[1, #]

[1]Centre for Proper Housing of Poultry and Rabbits, VPHI Institute, University of Bern, Zollikofen, Switzerland

[2]Graduate school of Cellular and Biomedical Sciences, University of Bern, Bern, 3012 Switzerland

[3] Department of Production Animal Clinical Science, Faculty of Veterinary Medicine, Norwegian University of Life Sciences (NMBU), Ås, Norway

[4] Department of Paraclinical Sciences, Faculty of Veterinary Medicine, Norwegian University of Life Sciences, Ås, Norway

[#]Current Address: Centre for Proper Housing of Ruminants and Pigs, Federal Food Safety and Veterinary Office, Agroscope, Posieux, Switzerland.

*Corresponding author

Email: alex.johny@unibe.ch

Abstract

Ramps facilitate earlier access to complex environments and increase early life voluntary exercise, which may positively affect the ~~brain and~~ cognitive development of chickens. This ~~exploratory~~ study focused on quantifying individual differences in ramp use and its impact on spatial cognition of laying hen pullets. Sixteen identical pens were housed with~~We used 16 identical pens holding 22~~ Lohmann Selected Leghorn (LSL) chicks of which eight chicks from each pen were colour marked from one day of age (DoA) to serve as focal birds. We quantified overall ramp use (walk/run, wing-assisted incline running, and jump/fly to and from ramps) by scan sampling recorded videos for 6, 10, 12, 20, 27, 41, and 55 DoA for all focal birds. From 56 to 95 DoA, long and short-term spatial memory~~ies~~ ~~spatial cognition~~ of three focal birds per pen were assessed in a holeboard test in three consecutive phases: cued, uncued and reversal. Mixed model ~~Our~~ analysis showed no consistency in average ramp use per day for individual birds. However, we found that the spatial cognitive abilities of the birds were linked to differences in ~~mean~~ ramp use frequency averaged across all observation days. Birds with higher ramp use made fewer reference (Estimate ± Confidence Interval = 0.94 [0.88, 0.99], p = 0.08) and working memory errors (Est ± CI = 0.77 [0.59, 1.00], p = 0.06) in the cued phase than birds with lower ramp use. In contrast, birds with higher ramp use made more reference memory errors (Est ± CI = 1.10 [1.01, 1.20], p = 0.05) in the reversal phase. Birds with higher ramp use also made more reference memory errors compared to birds with lower ramp use as the phases changed from ~~both~~ cued to uncued (p = 0.001~~7~~)~~ and uncued to reversal (p = 0.02)~~. Our results indicate that there might be a relationship between early life ramp use and spatial cognition of laying hens.

Key words: chicken, rearing environment, habitat complexity, behaviour, holeboard test

~~1.~~ Introduction

Spatial cognition is the ability of animals to acquire, use, recall and revise information concerning the spatial layout of their environment (1). It is critical for an animal's survival, as it enables navigation through the environment, locating resources such as food, water, shelter, conspecifics, and avoiding predators. Early life experiences play a considerable role in the development of spatial cognitive abilities of animals (2,3) and the complexity of the habitat in this period is a defining feature. Rearing animals in complex environments has been shown to improve spatial learning and memory (4,5), as well as having positive developmental effects on the brain regions involved in spatial cognition in a range of taxa (rat: (6), atlantic salmon: (7), mice: (8)).

The domestic hen is an excellent model species to study the development of spatial cognitive abilities due to their precocial nature (9). Precocial birds can be tested for cognitive functions from a very early age as they hatch with a fully developed sensory-motor system and rapid learning mechanisms (10). Hens are able to navigate complex environments using landmarks (11), egocentric information (12) as well as topographical features of the environment (13). They are also able to use multiple landmarks for orientation, possibly by forming cognitive maps (14). As in several other species, the development of spatial cognition in domestic chicks has also been shown to be influenced by early life environment. Chicks reared in complex environments by either equipping the housing system with elevated perches (15) or ground level visual barriers (16) have been shown to improve their spatial skills in navigating vertical spaces later in life (15) and their egocentric orientation (16). The visual barriers also induced changes in morphology of the hippocampus (17), the brain region involved in regulation of spatial navigation and memory (9).

Spatial cognition of domestic hens has been of scientific interest in applied research recently (18,19), owing to the changes in housing conditions over the last three decades (20). An increasing number of laying hens are being housed in cage-free systems such as aviaries (21),

which are structurally complex compared to cage systems (both battery and enriched) due to the vertically stacked tiers, within which essential resources such as perches, litter, food and water are located. Rearing birds in complex aviaries has been shown to benefit the spatial cognition (22) and working memory (23) ~~of laying hens~~ of laying hens. However, the access to the three-dimensional environment is compromised during the early life period of the laying hen chicks, as they lack the physical and motor skills to access the elevated surfaces of the aviary (24–26). Producers also generally confine the chicks to the lowest aviary tier for the first two to four weeks of age (WoA), as they want the chicks to stay near food, water, and heat sources, thereby restricting the access to complex environments in their early life (27). To facilitate access to elevated surfaces in the early life of domestic chicks, studies have investigated the provision of ramps, which provide a continuous path connecting the different tiers of the aviary (28,29). Providing ramps has been shown to facilitate the chicks' access to the elevated surfaces from the first WoA in multi-tiered aviaries without any negative consequences on mortality or welfare indicators (28,29).

Facilitating early access to elevated surfaces using ramps might also have positive consequences on the development of spatial cognition of laying hens. Provision of ramps in aviaries might influence the spatial cognition of the birds mainly in two ways. First, ramps provide earlier exposure to vertical complexity. Laying hen chicks reared with ramps have been shown to use elevated surfaces earlier compared to birds reared without ramps (28,29). Early exposure to vertical complexity aided by ramps might provide earlier opportunities for spatial learning, which in turn can influence cognition and neural development. Second, ramps promote early life voluntary exercise in domestic chicks. Chicks perform more vertical movements between the tiers of aviaries when provided with ramps, with most of these movements happening through ramps (28). Ramp use is not limited to tier change; chicks also walk, run, jump to and from, ~~Ramp use is not limited to tier change; chicks also In addition to ramps being used for inter-tier transitions, chicks also use the ramps actively by walking,~~

~~running, jumping to and from ramps,~~ and perform~~ing~~ wing-assisted inclined running on ramps ~~which does not result in a~~ a ~~tier change~~ from at least ~~from~~ three days of age (DoA) (30). The increase in early life voluntary exercise has been shown to improve adult hippocampal neurogenesis and spatial memory in rodents (31–33), humans (34,35) and fish (36–38). Although not directly investigated in hens, it is reasonable to assume that early life voluntary exercise facilitated by ramps ~~might~~ would positively influence the development of spatial cognition of laying hens~~ as well~~.

Individual differences in laying hens and their importance in cognition ~~and their importance of accounting for these differences in the cognition~~ have received a recent increase in interest~~ve studies has piqued interest recently~~ (39,40). Individual hens differ in the vertical movements and use of resources including the elevated surfaces~~ and the vertical movements they perform within~~ within the aviary (41,42). Consistent differences in behaviours such as exploration (43,44) and activity (41), have been established in domestic hens, which has been shown to influence the movement behaviour in various species (45,46). It is possible that the~~se individual differences in movement behaviour and activity can result in individual differences in ramp use, which~~ might drive differences in ramp use among individuals, potentially leading to ~~different~~ individual developmental trajectories for spatial cognition. For example, individual hens differ in the time spent on the free range which has been shown to positively correlate with cell proliferation in the rostral hippocampal sub-region (47). Hence, it is ~~hence~~ important to account for individual~~ level~~ differences in ramp use behaviour to better understand the impact of early life ramp use on the development of spatial cognition ~~of~~ in laying hens.

~~In the current exploratory study, we describe the relationship between the inter-individual variation in ramp use and the spatial cognition of laying hen pullets using a holeboard test.~~

The holeboard test has been used to assess the spatial discrimination learning abilities in

various species (48), including domestic hens (23,49). For the test, a subset of all the potential sites is food-rewarded and a bird can visit any of these sites in whatever order it chooses. The holeboard test provides measures for both short and long-term spatial memories. The reference memory is an indicator of long-term memory and holds trial-independent information such as the locations of food rewards and how to access these food rewards. The working memory and general working memory are measures of short-term memory and hold information that is relevant only for a specific trial such as the sites a bird has already visited in that trial.

To the best of our knowledge, there are no studies that have looked at the relationship between individual differences in ramp use and spatial cognition of laying hens. In the current study, we planned a first exploration of the potential relationship between the early life ramp use and spatial memories~~y~~ of laying hen pullets using a holeboard test. ~~As early life ramp use might lead to an earlier exposure to a complex habitat and promote early life voluntary exercise, which can positively benefit the development of spatial cognition, we expected that the individuals with higher frequency of ramp use might have better short and long-term memories than individuals with lower ramp use frequency.~~

~~2.~~ Methods

~~2.1.~~ Ethical approval

The experiment met the federal and cantonal regulations for the ethical treatment of animals involved in research and was approved by the Veterinary Office of the Canton of Bern, Switzerland (BE 106/19).

~~2.2.~~ Animals and Housing

Sixteen custom-built, identical pens (2.00 m × 2.00 m × 2.50 m (L × W × H)) situated at the Aviforum research facility, Zollikofen, Switzerland were used to house a total of 351 Lohmann Selected Leghorn (LSL) chicks from one day of age (DoA) until 17 weeks of age (WoA). All pens housed 22 birds ~~each~~, except for one pen which only had 21 chicks, due to a mistake in chick delivery~~.~~. Each pen ~~(schematics of pen provided in Figure 1, Johny et al. (Johny et al., 2023))~~ was fitted with two vertically stacked tiers at heights of 0.25 m and 1.20 m from the floor, and two round metal perches (2.00 m × 0.34 cm (L × diameter)) at 0.28 m and 0.55 m above the second tier. The tiers in each pen were connected by two ramps (1.30 m × 0.24 m (L × W), at an angle of 35˚) made of metal grids (0.65 m × 0.25 m (L × W)). Both tiers were made of plastic grids with the first tier (2.00 m × 1.15 m (L × W)) being wider than the second (2.00 m × 0.60 m (L × W)). The pen floors were covered with wood shavings, ~~which served as a litter area~~ (2.00 m × 0.75 m (L × W)). The pens were visually isolated from each other using opaque ~~plastic~~ ~~barriers made of~~ metal sheets that covered up to 1.50 m of the pen walls from the floor. The rest was covered with ~~and~~ plastic sheets ~~that covered the rest~~. The schematics of the pens and more description can be found in Johny et al. (2023). Olfactory and auditory contact was still possible between pens. Birds were populated on the first tier and confined there using wire mesh until four DoA. This was done to ensure that the birds learn the location of the drinkers and feeder. Feed was provided *ad libitum* on a feeder plate located on the first tier until two WoA, after which the feed was provided from a dispenser placed in the litter area. An additional feeder plate (40 cm diameter, 5 cm height) on the second tier was provided until five WoA. Water was provided *ad libitum* using nipple drinkers on the first tier. The birds were fed with starter feed (Egli Mühlen AG, Nebikon, Switzerland) from one until nine WoA and pullet feed (Egli Mühlen AG, Nebikon, Switzerland) from nine until 17 WoA. A light bulb (Silox basic, 120 – 3000K) attached to each pen ceiling ensured uniform illumination of 40 lux throughout the observation period. There was no provision of natural light. The light schedule followed standard rearing

management for LSL (27) pullets with 24 hours of light for the first two days of life, which was gradually reduced to nine hours in the fifth WoA and stayed the same until the conclusion of the project. The dimming phase of the light lasted for five minutes at~~in the ~~dawn and 30 minutes at~~in the ~~dusk~~ phase~~. Birds were vaccinated according to the standard rearing management protocol for LSL birds (Infectious bronchitis-primer, Marek's disease, Gumboro at one DoA at the hatchery, Paracox at five DoA, Infectious bronchitis 4/91 at 16 DoA, Infectious bronchitis Ma5 at 65 DoA, Avian encephalomyelitis at 93 DoA, Poulvac E.coli at 103 DoA and inactivated Infectious bronchitis vaccine at 114 DoA).

During population at one DoA, eight birds per pen were arbitrarily chosen as focal individuals. Each focal individual was marked with animal marking spray (RAIDEX, Hauptner, Switzerland) and tagged with numbered leg rings that were 5 mm in diameter (Flexi-Ringe, Fieger AG, Switzerland). We used green, black, and brown colours to mark the birds. Green and brown colours were used to mark two focal birds per colour per pen by applying a dot either on the back or neck region. Black colour was most recognizable on the chicks especially at younger ages and was used to mark four focal birds per pen. Four patterns were used with black colour – one dot on neck or back region, one dot on both neck and back, and a continuous line that ran from the neck to back. The colour marks were reapplied at 3, 8, 15, 29 and 43 DoA. The leg rings were changed to 8, 12 and 14 mm in diameter at 8, 29 and 43 DoA, respectively. No aggression towards focal birds was observed and no difference in body mass was found between focal and non-focal birds at 14 WoA.

Birds used in the current experiment were also part of another experiment that investigated artificial cues to encourage early life ramp use in laying hen chicks (30). This experiment had four treatment groups, that investigated the use of artificial cues in which three were exposed to artificial cues while one served as a control group devoid of any cues. Because this other experiment's outcome~~s~~ indicated an impact of the treatment on ramp use, we mitigated the

influence of treatment group-related variance by incorporating it as a control variable in the mixed-models used for statistical analyses.

2.3. Data Collection

2.3.1. Ramp use

Each pen was fitted with a video camera (Samsung SCO-2080R, IR, Samsung Techwin CO., Korea) connected to customized recording software (Multieye Hybrid Recorder Version 2.3.1.8, Artec Technologies AG, Diepholz, Germany) that recorded the videosbehaviour of birds at regular intervals until nine WoA. We assessed behaviours of focal birds (n = 8/pen) by counting inter-tier transitions and active behaviours on ramps, as described in Table 1 by Johny et al. (30) Inter-tier transitions were when birds moved between tiers using ramps. Active behaviours on ramps included walking, running, wing-assisted incline running (WAIR), or jumping, not resulting in a transition. We didn't differentiate between upward and downward transitions, and counted active behaviours as separate events if they were at least five seconds apart.

Both behaviours were counted from recorded videos at 6, 10, 12, 20, 27, 41, and 55 DoA for eight observation bouts of three minutes each dispersed evenly across the light period per day (total observation time per individual per day = 24 minutes).

2.3.2. Holeboard test

General principles: We used the holeboard test to measure the reference, working and general working memory of the birds in three different phases following initial habituations to a food reward and the test arena. The first phase was cued and served to test the birds' ability to learn the location of three rewarded cups out of a total of eight cups with colour cues added to the rewarded cups. The second phase was uncued and involved learning the same rewarded

locations as in the cued phase, but without the provision of colour cues. In the third, reversal phase, the locations of the rewarded cups were changed, and the birds had to replace the information of location of the rewarded cups they had acquired during the cued and uncued phase with a new one. There were no colour cues associated with rewarded cups in the reversal phase. The birds received 10, 20, and 10 trials each to learn the spatial configuration of the food reward for the cued, uncued, and reversal phases, respectively.

Arena and procedure: We tested three focal birds per pen (n = 48) from 56 – 95 DoA in the holeboard test. The arena design and testing methods for the holeboard test were adapted from Dumontier et al. (50). Two arenas were used to test two birds simultaneously which were situated in a separate experimental room adjacent to the barn. The arenas were enclosed by 2 m high walls made of tarpaulin on all sides and had a door on the side (Fig. 1A).Hence, birds were visually but not auditorily isolated during the testing procedure.~~The arenas were hence visually isolated, but the birds could still hear each other~~. On the floor of each arena ~~(350 × 170 cm (L × W))~~, eight circles ~~(50 cm in diameter)~~ distributed equally spaced in a 2 × 4 matrix~~, separated by 20 cm from each other~~ were drawn with black permanent markers (Fig. 1B). In the centre of each circle, a small cup attached to a plywood platform ~~(20 cm × 20 cm)~~ was placed and was used to provide grape pieces as the food reward during the test. Each arena had a start box that was connected to a pulley system allowing the observer to lift both start boxes simultaneously. Both arenas were equipped with a camera (Samsung SCO-2080R, IR, Samsung Techwin CO., Korea) connected to computer screens located outside the arena but within the experimental room allowing simultaneous viewing, recording, and coding of birds' behaviour by two researchers while birds were tested in parallel using both arenas. The researchers were trained on holeboard test videos obtained from Dumontier et al. (50) to establish inter-observer reliability before the experiment began. For the whole procedure, home pens were randomly assigned to an arena and observing researcher. Once assigned, the birds were always habituated and tested in the same arena and handled and scored by the

same researcher (i.e., 8 pens or 24 birds assigned per arena and researcher). The number of grape pieces eaten and each visit of the hen to a cup were scored. If at least half the body of the hen was inside the circle and the head was oriented towards the cup, it was considered a visit.

Fig~~ure~~ 1. Schematic representation of the holeboard arena: (1A) Two birds were tested simultaneously. The black arrows represent the direction of movement of rope and pulley system used to lift the start boxes. (1B, adapted from Dumontier et al (50). The dimensions of the arena and the general spatial configuration of the cups as viewed from above. The cups were numbered from 1 to 8. The rectangles represent the platforms of the cups.

Formatted: Font: Not Bold

Habituation: The focal birds were habituated to grape pieces in their home pens using the same cups as in the test. The habituation was performed over two consecutive days, with three sessions of 20 minutes per day. After habituation to the food reward, six focal birds per pen were arbitrarily~~randomly~~ chosen and habituated to the test arena in groups of three over three sessions. The first session lasted for 10 minutes and the other two was five minutes each. Each cup contained three grape pieces during the group habituation. Following the group habituation, the birds were individually habituated to the arena. Each cup contained one grape during the individual habituation. After three individual habituation sessions of five minutes each, three birds per pen that ate the most grape pieces over the three habituation sessions were selected for the test. The selected birds were further habituated to the arena until they ate from all the cups. The holeboard test was conducted in three separate phases as follows:

Cued phase (trials 1 - 10): Three cups out of the eight cups were baited with a piece of grape. To control for the odour cues, a piece of grape was placed in a hidden compartment below the bottom of each unbaited cup. To provide cues associated with the food reward, the plywood platforms were red in colour for the baited cups, while the unbaited cups had no colour cues associated with them. Three configurations of the baited cups (i.e., the spatial position of the

baited cups in the arena) were randomly chosen and assigned to the three focal birds selected for the tests from each pen (i.e., one configuration per bird). The birds were tested in a random order on each test day. At the start of each test, the birds were placed under the start boxes in both arenas by the researchers and doors of the arenas were closed. The test began when the start boxes were lifted by one of the researchers and ended when both birds had consumed the food reward from all three cups, or a maximum of five minutes had elapsed. The birds were returned to the home pen after each trial and were given 10 trials over five days to learn the configuration of the rewarded cups.

Uncued phase (trial 11 – 30): The same reward configuration and test procedure were used for both the cued and uncued phases, but the baited cups no longer had any visual cues in the latter as the plywood bases of all cups looked the same. The birds were given 20 trials over 10 days to learn the reward configuration without visual cues.

Reversal phase (trial 31 – 40): To get to the reversal phase, birds had to reach a success criterion calculated as the ratio of number of visits to baited cups to total number of visits to all cups. The ratio indicates the ability of the bird to distinguish between rewarded and unrewarded cups and can be used as a measure of learning performance (48). Birds that had reached a score of 0.6 or more in three out of four consecutive trials in the uncued phase were subjected to the reversal phase. The reversal phase lasted for 10 trials over five days and the birds had to find the food reward in a new configuration of uncued, baited cups thus measuring the ability of the birds to unlearn the old and learn the new reward configuration. Three new reward configurations were randomly chosen and assigned to the birds that met the success criterion from each pen (i.e., one configuration per bird). Some pens had less than three birds as not all birds met the success criterion.

Data processing: For each trial, we performed the following calculations:

1. Reference memory errors: the sum of all visits and revisits to unrewarded cups. The reference memory errors indicate the ability of the birds to discriminate between rewarded and unrewarded cups.

2. Working memory errors: the revisits to rewarded cups (calculated as the difference between the number of rewarded visits and total number of visits to rewarded cups). It reflects the ability of the chickens to avoid re-visits to rewarded cups during a trial.

3. General working memory errors: total number of revisits to all the cups (calculated as the difference between the number of unique visits to rewarded and unrewarded cups and total number of visits to all cups). This indicates the ability of the chickens to avoid re-visits to already visited cups during a trial.

For all memory errors, greater values indicate a poorer performance, with zero being the best possible performance.

4. Trial duration: total time taken in seconds to consume the food rewards from all three cups. Duration was measured starting from the lift of the start box with a maximum of five minutes in case the birds failed to consume the rewards from all three rewarded cups.

5. Errors during phase change: to investigate the response of the birds to the change in phases (i.e., cued to uncued, uncued to reversal), we compared the number of memory errors of each bird from three trials before and after a phase change for all the three memory indices. ~~For the cued to uncued phase change, we used the last three trials of the cued phase (trial 8 – 10) and first three trials of the uncued phase (trial 11 – 13), and the last three trials (trial 28 -30) and first three trials (31 – 33) of the uncued and reversal phases respectively for the uncued to reversal phase change. As we expected the number of errors to have different directions in slopes in accordance with the phase, we grouped the two extremes of ramp users (i.e., the ten birds with the highest (HR) and the ten birds with the lowest (LR) mean ramp use frequency) to simplify the analysis and interpretation.~~

~~2.4.~~ Statistical analysis

All analyses were performed with R (version 4.1.1, R Core Team, 2021) and R studio (RStudio Team, 2021) as the graphical interface. We used 'lme4' (51) package to fit linear mixed models (LMM) and package 'glmmTMB' (52) to fit generalized linear mixed effects models (GLMM). The model assumptions for homogeneity of variance and normal distribution of errors were checked using the ‘Dharma’ (53) package. When the model assumptions were not met, the data were transformed. Continuous explanatory variables were centred to zero and categorical variables were sum-contrast coded to set the reference level of contrast calculation as the mean of all groups within a variable. This was done to obtain interpretable main effects, even in the presence of interactions. The p-values of each explanatory variable were calculated by reducing each model by the particular explanatory variable and comparing it to the full model using parametric bootstrap tests (package ‘pbkrtest’ (54)). The model estimates and confidence intervals were calculated using 'broom:mixed' package (55) and the package 'emmeans' was used to calculate estimated marginal means from the full models (56). We used the framework provided by Berner and Amrhein (2022), which uses a combination of effect sizes, confidence intervals and p-values to interpret ~~our~~ results. To reduce the numbers of tests, post-hoc comparisons were done qualitatively using numeric and visualizations of the estimated marginal means while also considering the raw data. ~~The repeatability of ramp use was calculated using 'rptR' package (Stoffel et al., 2017).~~ The 'tidyverse' package (58) was used for data cleaning and the package 'ggplot2' (59) for data visualization.

~~Repeatability~~Frequency of ramp use: We summed the number of transitions and active behaviours on ramps for individuals to obtain total ramp use per bird. We analysed the general ramp use behaviour of all focal birds as well as only the focal birds that underwent the holeboard test ~~individuals~~ descriptively. ~~To investigate whether individuals showed consistent~~

~~differences in ramp use behaviour, we calculated the repeatability of ramp use among individuals over all observation days (eight observation bouts per day with seven days for each individual). Repeatability was calculated as the ratio of variance explained by the focal birds to the total within-group (residual) variance (Hertel et al., 2020; Stoffel et al., 2017). To calculate repeatability, we split the analysis into two because of the high frequency of zeroes in the data. For the first analysis, we assigned a binary condition for each observation bout for all focal birds and fitted the model with a binary distribution (yes or no; depending on if the focal bird used ramps in that observation bout or not). The second analysis only included the non-zero observations of ramp use by the focal birds and was fit with a Poisson distribution. Additionally, we also calculated repeatability by using mean ramp use over each observation day for each focal bird as a response variable and fitted the model with a Gaussian distribution.~~

~~The analyses were performed including all focal birds as well as only including the focal birds that underwent the holeboard test. Each model included treatment as categorical (to account for differences from treatment groups as detailed in Johny et al. (2023) (Johny et al., 2023)) and DoA as continuous (6, 10, 12, 20, 27, 41, 55 DoA) explanatory variables. Bird ID crossed with date was included as a random factor for each model. Confidence intervals for repeatability estimates were calculated by parametric bootstrapping. We deemed repeatability estimates significant if the 95% confidence intervals excluded zero.~~

The association between holeboard measures and ramp use: For the cued and uncued phase, data from all focal birds that underwent the holeboard task (n = 48) and for reversal phase the data from focal birds that met the success criteria (n = 37) were included in the analysis. The reference, working and general working memory errors, and trial duration for each bird and trial were used as response variables and were analysed separately for each phase. To relate ramp use frequency to holeboard parameters, the mean ramp use frequency (MRF) was

calculated by taking mean ramp use over all observation days per focal bird and used as a continuous explanatory variable. Each model contained MRF per individual and trial number (cued: 1 - 10, uncued: 11 - 30, reversal: 31 – 40) as continuous variables as well as their interaction as explanatory variables. Configuration was added as a control variable in each model, as preliminary visualizations revealed differences in holeboard measures between different configurations. Trial number nested in bird ID nested in pen was used as random factor in all models. The models were fit with Poisson distribution for cued and reversal phase for reference memory errors, cued phase for working memory errors, and reversal phase for general working memory errors. Uncued phase of reference memory errors, cued and reversal phase of working memory errors, and cued and uncued phase of general working memory errors were fit with a negative binomial distribution. All phases of trial duration were fit with a Poisson distribution.

The effect of phase change on reference, working, and general working memory errors were analysed separately for both phase changes. The trials and birds for the phase change analysis were chosen as per point five under the 'data processing' section. All models included an interaction of MRF~~ramp use group (HR, LR)~~ and respective phases (for ex., cued and uncued for cued to uncued phase change analysis) as categorical explanatory variables. Reward configuration was added as a control variable and trial number nested in bird ID nested in pen was included as a random factor in all models.

The data and code for all analysis can be found at https://osf.io/9j2g8

Field Code Changed

~~3.~~ Results

~~3.1.~~ Ramp use

~~*All focal birds:*~~ The mean ~~frequency of~~ individual ramp use over 56 observations (eight~~8~~ bouts per seven~~7~~ observation days) was 0.39 uses of ramps per observation bout (i.e., per three~~3~~ minutes) for all ~~the~~ focal birds (n = 128). The mean individual ramp use per bout ranged from 0.09 to 1.00 with a standard deviation of 0.16. Of the 7,186 observations ~~(eight observation bouts from seven days for 128 focal birds)~~, 79% of the observations were zeroes (5,698). ~~There was no repeatability for whether focal birds used ramps in a given observation bout or not (binary outcome, repeatability (r = 0.005, CI [0.00, 0.01]). There was no repeatability in ramp use for focal birds for non-zero observations (r = 0.00, CI [0.00, 0.01]), as well for ramp use averaged over DoA per bird (r = 0.04, CI [0.00, 0.09]).~~

~~*Focal birds that underwent holeboard test:*~~ The mean ~~frequency of~~ individual ramp use of focal birds (n = 48) that underwent the holeboard test~~:~~ was 0.38 uses of ramps (range 0.09 – 0.67) per observation bout ~~(i.e., three minutes)~~ with a standard deviation of 0.14. Eighty percent (2,153 observations) of the total 2,688 observations ~~(eight observation bouts from seven days for 48 birds)~~ were zeros. ~~There was no repeatability for whether birds used ramps or not within an observation bout (r = 0.00, CI [0.00, 0.01]). No repeatability was found for all non-zero observations of ramp use (r = 0.00, CI [0.00, 0.04]) and ramp use averaged per DoA per bird (r = 0.00, CI [0.00, 0.01]).~~

~~3.2.~~ Holeboard test

Reference memory errors: Birds with higher MRF demonstrated a decrease in errors compared to birds with lower MRF during the cued phase, although statistical evidence was limited (model estimates (Est) ± CI = 0.94 [0.88, 0.99], p = 0.08, Fig. 2). For example, ~~T~~the bird with the lowest MRF made slightly more errors [Estimated marginal means (EMM) ± CI = 3.36 [2.52, 4.48]) than the ones with median (2.95 [2.61, 3.35]) and highest (2.55 [1.91, 3.41]) MRF. The number of errors also decreased with trial number (Est ± CI = 0.75 [0.76,

0.85], p = 0.001) in the cued phase. The interaction of MRF and trial number did not ~~seem to~~ influence the number of errors made in the cued phase (Est ± CI = 0.97 [0.32, 0.92], p = 0.31). In the uncued phase, the number of errors reduced with trial number (Est ± CI = 0.93 [0.89, 1.07], p = 0.001), but there were no influences of MRF (Est ± CI = 1.01 [0.96, 1.07], p = 0.74) or the interaction between MRF and trial number (Est ± CI = 1.00 [0.97, 1.04], p = 0.98). In the reversal phase, the birds with higher MRF made more errors than the birds with lower MRF (Est ± CI = 1.10 [1.01, 1.20], p = 0.05, Fig. 3). The bird with the lowest MRF made slightly fewer errors (EMM ± CI = 2.72 [1.82, 4.07]) than the ones with median (3.30 [2.80, 3.90] and high (4.01 [2.78, 5.77]) MRF. With limited statistical support, the number of errors slightly decreased with trial number (Est ± CI = 0.95 [0.9, 1.01], p = 0.08) in the reversal phase. The interaction of MRF and trial number did not have any effect on the number of errors made in the reversal phases (Est ± CI = 0.99 [0.94, 1.04], p = 0.82).

Fig~~ure~~ 2: The relationship between reference memory errors (sum of all visits and revisits to unrewarded cups) and mean individual ramp use frequency in the cued phase (model estimates ± CI = 0.94 [0.88, 0.99], p = 0.08). The black points connected by the black line represent the estimated marginal means and the shaded ribbon represents 95 % confidence intervals. The coloured points represent reference memory errors (all visits – visits to rewarded cups) of focal birds' colour-graded according to mean ramp use frequency.

Formatted: Font: Not Bold

Fig~~ure~~ 3: The relationship between reference memory errors (sum of all visits and revisits to unrewarded cups) and mean individual ramp use frequency in the reversal phase (model estimates ± CI = 1.10 [1.01, 1.20], p = 0.05). The black points connected by the black line represent the estimated marginal means and the shaded ribbon represents 95 % confidence intervals. The coloured points represent reference memory errors (all visits – visits to rewarded cups) of focal birds, colour-graded according to mean ramp use frequency.

Formatted: Font: Not Bold

Working memory: In general, all birds made very few working memory errors throughout all phases (Mean ± SD: cued – 0.33 ± 0.69, uncued – 0.23 ± 0.64, reversal – 0.34 ± 0.74). With weak statistical support, the birds with higher MRF made marginally fewer errors than birds with a lower MRF (Est ± CI = 0.77 [0.59, 1.00], p = 0.06, Fig. 4) in the cued phase. The bird with the lowest MRF (EMM ± CI = 0.47 [0.15, 1.45]) made slightly more errors than the ones with median (EMM ± CI = 0.27 [0.15, 0.47]) and the one with the highest MRF (EMM ± CI = 0.15 [0.05, 0.52]). There was a slight reduction in the number of errors as the trial number increased, although the statistical support for this find was weak (Est ± CI = 0.84 [0.69, 1.03], p = 0.08). The interaction between trial number and MRF did not a have an effect number of working memory errors in the cued phase (Est ± CI = 0.94 [0.78, 1.15], p = 0.80). In the uncued phase, the number of errors decreased with trial number (Est ± CI = 0.87 [0.76, 0.99], p = 0.05). No influence of MRF (Est ± CI = 1.07 [0.86, 1.39], p = 0.12) or the interaction between MRF and trial number (Est ± CI = 0.95 [0.84, 1.08], p = 0.45) was found on the number of errors made in the uncued phase. In the reversal phase, the number of errors made by the birds was not influenced by MRF (Est ± CI = 1.03 [0.78, 1.36], p = 0.87), trial number (Est ± CI = 0.83 [0.67, 1.06], p = 0.14) or their interaction (Est ± CI = 0.84 [0.66, 1.05], p = 0.14).

Fig~~ure~~ 4: The relationship between working memory errors (sum of all revisits to rewarded cups) and mean individual ramp use frequency in the cued phase (model estimates ± CI = 0.77 [0.59, 1.00], p = 0.06). The black points connected by the black line represent the estimated marginal means and the shaded ribbon represents 95 % confidence intervals. The coloured points represent working memory errors (all visits to rewarded cups – rewarded visits) of focal birds, colour-graded according to mean ramp use frequency.

General working memory: Birds made very few general working memory errors in all three phases (Mean ± SD: cued – 0.73 ± 1.56, uncued – 0.62 ± 1.55, reversal – 0.86 ± 1.61). The

Formatted: Font: Not Bold

number of errors made by birds decreased with trial number with varying statistical support for each phase: cued (Est ± CI = 0.69 [0.56, 0.84], p = 0.003), uncued (Est ± CI = 0.85 [0.72, 1.04], p= 0.08) and reversal (Est ± CI = 0.87 [0.78, 0.97], p = 0.02). No influences of MRF (Est ± CI, cued = 0.80 [0.61, 1.04], p = 0.12, uncued = 1.10 [0.86, 1.39], p = 0.46, reversal = 1.17 [0.90, 1.53], p = 0.30) and the interaction between MRF and trial number (Est ± CI, cued = 0.89 [0.70, 1.10], p = 0.28, uncued = 0.99 [0.83, 1.19], p = 0.93, reversal = 0.94 [0.84, 1.06], p = 0.30) were found for any of the phases.

Trial duration: The trials in which the birds failed to consume from all baited cups were removed from the analysis (9, 4 and 1 out of 480, 960 and 370 observations from cued, uncued and reversal phases, respectively). Trial duration was influenced by an interaction between MRF and trial number (Est ± CI = 0.88 [0.84, 0.93], p = 0.001) in the cued phase, with birds with a high MRF showing a faster decline in trial duration with increasing trial number (Estimated slopes ± CI for selected MRF: lowest = - 0.19 [- 0.24, - 0.13], median = - 0.29[- 0.32, -0.27], highest = - 0.40 [- 0.45, −0.34]). Trial duration decreased with increasing trial number in the uncued (Est ± CI = 0.99 [0.98, 1.00], p = 0.001) and reversal (Est ± CI = 0.93 [0.91, 0.95], p = 0.001) phases. No effect of MRF (uncued = 1.05 [0.53, 2.02], p = 0.33, reversal = 0.18 [0.02, 2.03], p = 0.20) nor the interaction of MRF and trial number (uncued = 1.00 [0.99, 1.02], p = 0.78, reversal = 1.07 [1.01, 2.03], p = 0.79) were found for trial duration for the uncued and reversal phases.

Phase change: The interaction between phase change and MRF~~ramp use group (i.e., high (HR) and low (LR) ramp user~~) had an effect on the reference memory errors (p = 0.001~~7~~) for the cued to uncued phase change with ~~HR~~ birds with a higher MRF making more errors than the ~~LR~~ birds with a lower MRF as the phase changed from cued to uncued~~for this phase change~~ (Fig 5A~~Estimated marginal means (EMM) presented in Table 1~~). For the uncued to reversal phase change, there was no~~an~~ effect of the interaction between phase change and

MRFramp use group on the reference errors (p = 0.1702) with HR birds making more errors than LR birds with phase change (EMM in Table 1). The interaction between phase change and ramp use group had no effect on the working memory scores for both phase changes (cued to uncued, p = 0.21, uncued to reversal, p = 0.32). For the general working memory, the interaction between phase change and ramp use group showed an effect for the cued to uncued (p = 0.0206), but not for the uncued to reversal phase change (p = 0.16). The HR birds with the lower ramp use made marginally more errors than the ones with higher LR ramp usebirds as the phase changed from cued to uncued for the general working memory (Fig 5BEMM in Table 1).

Fig 5: The relationship between of ramp use frequency and spatial memory errors (A) Reference memory, B) General working memory) as the phase changed from cued to uncued phase. The point ranges represent the estimated marginal means of reference memory errors and the points are the raw data, colour graded according to mean ramp use frequency.

Table 1: Estimated marginal means for the interaction between ramp use frequency group (high vs. low ramp users) and phase for the phase change analysis of reference and general working memories from the holeboard test. Results are presented for the first quartile (0.2819), median (0.378) and third quartilehighest (0.4753) of mean ramp frequency.

Holeboard measure	Phase change analysis	Phase	Ramp use frequencye group	Estimated marginal means (EMM) ± CI
Reference memory	Cued to uncued	Cued	First quartileHigh ramp use	2.455 [2.195, 2.733.02]

			MedianLow ramp use	2.2417 [2.011.83, 2.519]
			Third quartile	2.021.91 [1.75,51, 2.3340]
		Uncued	First quartileHigh ramp use	3.4300 [3.102.46, 3.8066]
			MedianLow ramp use	3.482.98 [3.182.54, 3.8248]
			Third quartile	3.542.91 [3.182.52, 3.963.35]
General working memory	Cued to uncued	Cued	First quartileHigh ramp use	0.45 [0.261, 0.7710]
			MedianLow ramp use	0.371[0.21, 0.6347]
			Third quartile	0.291 [0.1609, 0.540]
		Uncued	First quartileHigh ramp use	0.7550 [0.4423, 1.2907]
			MedianLow ramp use	0.7347 [0.4532, 1.160.67]
			Third quartile	0.69 [0.4021. 1.190.94]

4. Discussion

The aim of the study was to~~ to~~ perform a first exploration into ~~describe~~ the relationship between ~~inter-individual variation in~~ early life ramp use (1-8 WoA) and short and long-term spatial memory~~iescognition~~ in laying hen pullets~~. Even though ramp use behaviour was not consistent over the first eight WoA across the early life period for individual birds, we found that the spatial cognitive abilities of the birds were weakly associated to individual differences in ramp use over the first eight WoA~~. We found that the birds that used ramps more made fewer reference and working memory errors in the cued phase than birds with lower ramp use. However, birds with higher ramp use made more reference memory errors in the reversal phase. The birds that used ramps more often also showed a faster decline in time taken to solve the holeboard test with increasing trial numbers in the cued phase. For the phase change analysis, we found that birds with a higher ramp use made more reference~~ memory errors in both phase changes and, more~~ and general working memory errors in the cued to uncued phase change than the birds that used ramps less often. Our results indicate a relationship between early life ramp use and spatial memories~~cognition~~ in laying hens, which can have several explanations.

The relationship between ~~E~~early life ramp use and~~is associated with~~ spatial memories~~cognitive measures from the holeboard test~~

Formatted: Font: 16 pt

~~As exposure to a complex environment (Kihslinger and Nevitt, 2006) as well as voluntary exercise (fishes;34, rodents;37,human; 57–59) in early life have been shown to improve spatial cognition in a range of taxa, it could be expected that birds with higher ramp use might have better reference, working and general working memory scores than the ones with lower ramps use. Our findings indicate an association between early-life ramp utilization and cognition; however, they do not provide a definitive indication that using ramps in early life~~

enhances spatial cognition in laying hen pullets. Therefore, our results remain open to various interpretations, allowing for multiple explanations.

One possible explanation for the association between holeboard measures and ramp use might be that the individual differences in both ramp use and cognition are driven by consistent inter-individual differences in behaviour. Consistent differences in behaviours such as boldness, exploration and activity have been shown to correlate to movement in animals (46,60). For example, bolder bank voles (61) and Trinidad killifish (62) move longer distances, fast exploring female great tits disperse to a greater distance (63), and high exploratory laying hens use the outdoor range more than the ones that are less exploratory (64,65). It is possible that individual differences in ramp use might also be driven by individual differences in activity, exploration, and fearfulness, with more active, explorative, and less fearful birds showing more use of ramps.

The speed-accuracy trade-off proposed by Sih and Del Giudice (66) is one of the main frameworks for explaining the relationship between consistent inter-individual differences in behaviour and cognition. It predicts that fast behavioural types (with high exploration and activity levels, neophilia, etc.) tend to prioritize speed over accuracy and learn simple tasks such as associative learning faster. However, they are less cognitively flexible and make more mistakes during challenging tasks, such as reversal learning. In contrast, slow behavioural types (with low exploration and activity levels, neophobia, etc.) tend to prioritize accuracy over speed, and while they are slower at simple learning tasks, they are more sensitive to changes in their environment and make fewer errors during reversal tests compared to fast behavioural types. Although the speed-accuracy trade-off is not studied in domestic hens, our results seem to align with this model because the cognitive styles of individuals with high and low ramp use resembles the one of fast and slow behavioural types, respectively.

~~More specifically, birds with higher ramp use made slightly fewer reference and working memory errors, and learned the task faster compared to individuals with lower ramp use in the cued phase, which can be viewed as a simple associative learning task because the rewarded cups were associated with colour cues. In contrast, birds with lower ramp use made fewer reference memory errors during the reversal phase, which is a more cognitively challenging task. Birds with lower ramp use also made fewer reference and general working memory errors in the cued to uncued phase change than the birds that used ramps more often. The lower number of reference memory errors, both during the reversal phase as well as in the phase changes, might imply that the birds with lower ramp use frequency show higher behavioural flexibility and are able to update their learning strategy with changes in environment. Lastly, marginally higher working memory errors of the birds with low ramp use in the cued phase might be also indicative of neophobia as cues were not present during the habituation.~~

The speed-accuracy trade-off, proposed by Sih and Del Giudice (66), is a key framework for understanding consistent inter-individual differences in behaviour and cognition. It suggests that fast behavioural types (with high exploration and activity levels, neophilia, etc.) prioritize speed over accuracy, acquiring simple rules quickly but making more mistakes in challenging situations like reversal learning. Conversely, slow behavioural types (with low exploration and activity levels, neophobia, etc.) prioritize accuracy, and while they are slower at simple learning tasks, they are more sensitive to changes in their environment and make fewer errors during reversal tests. While not studied in domestic hens, our results seem to align with this model. Birds with higher ramp use made fewer spatial memory errors and learned tasks faster in the cued phase, suggesting better associative learning (67). In contrast, birds with lower ramp use made fewer errors during the reversal phase, indicating higher behavioural flexibility (68). They also showed fewer memory errors during phase change from cued to uncued, suggesting an ability to update learning strategies with environmental shifts.

Additionally, the marginally higher working memory errors of birds with low ramp use in the cued phase may indicate neophobia, as cues were absent during habituation.

Empirical research however indicates that the relationship between consistent inter-individual differences in behaviour and cognition does not always follow the speed-accuracy trade-off framework and that the relationship is dependent on factors such as species, sex, and age (69). For example, Zidar et al. (44) showed a complex and age-dependent relationship between cognition and behavioural type in red jungle fowl. ~~that While our results seem to align with the speed-accuracy trade-off framework, studies in red jungle fowl have revealed the complexity of this relationship. For example, Zidar et.al (44) investigated the link between cognition and consistent individual differences in behaviour in red jungle fowl by conducting various learning (discriminative, reversal, and spatial learning) and behavioural tests (novel arena, novel object) on both young and adult red jungle fowl. While there was no correlation between learning speed and personality traits in discrimination and spatial learning tasks, results showed a complex and age-dependent relationship between cognition and behavioural type. When tested at a young age, chicks with higher exploration levels showed better performance in the reversal task than those with lower levels. The direction changed when the birds grew older as less exploratory adult females performed better in the reversal test than highly exploratory females. Age-related changes in exploratory behaviour of red jungle fowl have also been shown by Favati et al (2016) (70), who reported that exploration changes across major developmental stages, such as independence from the mother at around eight weeks of age. In our study, ramp use behaviour was recorded from one to eight WoA and the birds were tested for spatial cognitive skills from nine to 13 WoA. As domestic hens and red jungle fowl are closely related, the consistency in behaviours such as exploration and activity in domestic hens might vary similarly depending on age and ontogenetic changes as demonstrated in the red jungle fowl (65). A potential shift in traits like exploration and activity levels at eight WoA could imply that the use of ramps, as well as its connection to~~

cognitive traits, may have undergone changes from the period of measuring ramp use (1-8 WoA) to the assessment of cognitive traits (9-13 WoA). As red jungle fowls and domestic chicks are closely related, we may needWe thus need further studies across multiple agesseveral different developmental stages to elucidate the complex relationshiplink between consistent inter-individual differences in behaviour and cognition in laying hens.

Another possible explanation for our results could be that early-life ramp use promotes better spatial cognitive abilities. Birds with higher ramp use might have learned the holeboard test better than those with lower ramp use, as indicated by fewer errors in reference and working memories and a faster decline in trial duration during the cued phase. Furthermore, it was observed that birds with higher ramp use made more reference memory errors during the transition from uncued to reversal phase, whereas no difference in errors was evident among birds with lower ramp use during this phase transition. This outcome suggests that birds with lower ramp use might not have acquired knowledge of the rewarded locations to the same degree as their counterparts with higher ramp use during preceding phases. However, the

A L lower number of reference memory errors in the reversal phase for the birds with low ramp use could similarly be attributed to the aforementioned rationale. However, overall, the differences in ramp use was not associated with any of the spatial memoriesno learning differences between high and low ramp users were observed in the uncued phase. The lack of difference may be due to both groups of birds checking the unrewarded cups on their way to the rewarded cups, resulting in similar scores since the cost incurred in the process is low (23,50). Therefore, the scores in the uncued phase may not accurately reflect the learning performance of the birds.

Another possible explanation of our results might be that early life ramp use mightdid not have a meaningful influence on the spatial memories of the hens, a position . This is supported by the low effect sizes (or influence) of MRF on spatial memories (Reference

memory, cued phase: 0.94, reversal phase: 1.10). Moreover, we also didn't see any differences in the spatial memories in the uncued phase, which would be expected if the birds with different ramp use varied in their spatial cognition as well. As described within the context of the speed-accuracy trade off above, Tthe differences in spatial memories observed in the cued and reversal phase can be explained by a difference in associative learning and behavioural flexibility, respectively. In comparison to the cued and reversal phases, the performance in the uncued phase is directly related to spatial memories and has been shown to be controlled by the hippocampus (48), the region that plays a central role in processing spatial information (9). Hence the lack of differences in spatial memories in the uncued phase, combined with low effect sizes of reference memories in the cued and reversal phase suggests that early life ramp use might only have a minor impact on the spatial cognition of the pullets.

Consistency in ramp use behaviour

We expected ramp use to be repeatable, as previous work has shown that adult laying hens show long-term consistency in the daily vertical distance travelled in a multi-tier aviary (Montalcini et al., 2023). In addition, ramp use might also be driven by differences in exploration and activity, which has been shown to have consistent individual differences in domestic hens (exploration: 68, 65, activity: 69). However, our findings revealed no consistent variations among individuals in terms of ramp use. It is worth noting that individual behaviours in many species tend to be unstable during ontogeny and only become consistent after reaching maturity (Cabrera et al., 2021). For instance, the consistency of daily vertical distance travelled in an aviary system was low before reaching peak egg production (r = 0.38, WoA 18 - 19, (Montalcini et al., n.d.)) and only stabilized after sexual maturation (r = 0.66 for WoA 25 – 60, (Montalcini et al., 2023)). Favati et al. (Favati et al., 2016) found short-term consistency for exploration and foraging behaviour in the early life (4–8 weeks) of red jungle fowl but behavioural traits such as exploration and response to predators were

inconsistent until sexual maturation and only stabilized during adulthood. Similarly, Neave et. al (Neave et al., 2020) showed that individual consistency was poor across the major developmental periods of puberty for cattle (pre weaning to lactation), but showed short-term consistency during the early rearing period (pre- to post-weaning) and late period of development (puberty to lactation). Furthermore, the video analysis conducted in this study utilized relatively short observation periods (specifically, 3 minutes), which might have failed to capture the behavioural variation in ramp use among the birds, considering that 80% of the observations were zeroes. Although our study did not demonstrate the repeatability of ramp use during the early life period of laying hens, it is reasonable to expect short-term consistency in ramp use during this phase. Nonetheless, the results highlight the need for longer observation bouts to effectively capture the variation between individuals.

Evaluation of holeboard task

Formatted: Font: 16 pt

Unlike in rodents and pigs, the holeboard task is not widely used to measure the spatial cognition of hens and hence require a through consideration of the variations and limitations of the methods. Our holeboard test setup was similar to Dumontier et al. (50), except for the lack of two start box positions in the arena and the order of the phases as it was unuced, cued, overtraining and reversal phase for Dumontier et al. (50). Also, instead of including an overtraining phase as used by Dumontier et al. (50), we used a success criteria to make sure the birds had learnt the spatial location of the rewards before continuing with the reversal phase. We calculated memory errors rather than memory ratios like Dumontier et al. (50), because of the low variation in the working memory and general working memory ratios in our study. Our birds showed relatively high working (mean ± SD = 0.94 ± 0.13) and general working memory scores (0.93 ± 13) which were similar to Dumontier et al. (50) (working memory = 0.8 – 0.9, general working memory = 0.7 – 0.9), but slightly better than in more complex holeboard tests with a 3 × 3 matrix of cups (working memory = 0.7 – 0.8 (49) and

0.6 – 0.9 in (23)). The reference memory in our study was 0.55 ± 0.13, which again was similar to other studies (0.4 – 0.7; (23,44,45)). The similarity in scores with other studies, coupled with the decrease in reference memory errors throughout all phases suggest that the birds did learn the task.

As noted by Dumontier et al. (50) and Tahamtani et al. (23), one general criticism of the holeboard test as a method might be the low cost to check the unrewarded cups. There was only a marginal decrease in reference memory errors in the uncued phase from trial 11 to 30. The reference memory errors also showed a large variation across consecutive trials, which might be due to birds checking the all the cups in certain trials, as it incurs only a negligible energy expenditure. If indeed the birds were checking all the cups in certain trials, the lack of large differences in references memory errors observed in our study might not be indicative of lack differences in spatial memory. Future studies employing the holeboard test might benefit from adding additional costs, such as bigger arenas with higher proportion of unrewarded holes or birds requiringed to manipulate the hole, to obtain rewards. For instance, holeboard tests for pigs employ a ball to cover the food bowl, which the pigs have to manipulate using their snout to gain access to rewards underneath (70).

Another limitation specific to our study is the use of one start location throughout the test, which can result in egocentric approaches such as fixed food search patterns (48). In addition, the rectangular arena with a 2×4 matrix distribution of rewards, can lead to fixed simple food search patterns such as visiting the closest cup straight ahead.

Outlook

Formatted: Font: 16 pt

The current study contributes to the existing body of research exploring the relationship between early-life habitat complexity and spatial cognition in laying hens. The existing body of knowledge has Many studies have covered multiple levels of complexity (cage vs aviary

(23,50), floor vs three-dimensional rearing (15,71)) and ages for a set of spatial cognitive indices (spatial long and short-term memories (23,50), depth perception (71), neuro-motor coordination (15)). Nonetheless, the long and short-term impacts of early life habitat complexity on the spatial cognition of laying hens remains ambiguous. Future studies should account for effect of age, type of test used and include additional measures of brain development such as neural cell counts to better understand the impact early life habitat complexity has on the development of spatial cognition in laying hens. Furthermore, it is important to investigate the specific level of habitat complexity required to induce developmental changes in the spatial cognitive abilities of the birds. This aspect deserves further investigation to determine the optimal conditions for promoting spatial cognition in laying hens.

Acknowledgements

We thank Abdulsatar Abdel Rahman for helping with the holeboard task. Many thanks to Thomas Heinzel and Jan Büchler, for building the pens and arena, Masha Marincek and Josie Siegel for their help with the video analysis. We thank Lucille Dumontier for the discussions on the statistical analysis. We thank the Ana Rentsch for her valuable feedback that improved the quality of the manuscript. The work was supported by the European Union’s Horizon 2020 research and innovation programme under the Marie Skłodowska-Curie grant agreement No 812777. The document reflects only the author's view and the European Union’s Horizon 2020 research, and innovation programme is not responsible for any use that may be made of the information it contains.

References

1. Thinus-Blanc C. Animal Spatial Cognition. Anim Spat Cogn. 1996 Nov;

2. De Pasquale C, Neuberger T, Hirrlinger AM, Braithwaite VA. The influence of complex and threatening environments in early life on brain size and behaviour. Proc R Soc B Biol Sci. 2016 Jan 27;283(1823).

3. Buchanan KL, Grindstaff JL, Pravosudov V V. Condition dependence, developmental plasticity, and cognition: implications for ecology and evolution. Trends Ecol Evol. 2013 May 1;28(5):290–6.

4. Salvanes AGV, Moberg O, Ebbesson LOE, Nilsen TO, Jensen KH, Braithwaite VA. Environmental enrichment promotes neural plasticity and cognitive ability in fish. Proc R Soc B Biol Sci. 2013 Sep 22;280(1767).

5. Whiteside MA, Sage R, Madden JR. Multiple behavioural, morphological and cognitive developmental changes arise from a single alteration to early life spatial environment, resulting in fitness consequences for released pheasants. R Soc Open Sci. 2016 Mar 23;3(3).

6. Falkenberg T, Mohammed AK, Henriksson B, Persson H, Winblad B, Lindefors N. Increased expression of brain-derived neurotrophic factor mRNA in rat hippocampus is associated with improved spatial memory and enriched environment. Neurosci Lett. 1992 Apr 13;138(1):153–6.

7. Mes D, Van Os R, Gorissen M, Ebbesson LOE, Finstad B, Mayer I, et al. Effects of environmental enrichment on forebrain neural plasticity and survival success of stocked Atlantic salmon. J Exp Biol. 2019 Dec 1;22(23).

8. van Praag H, Kempermann G, Gage FH. Neural consequences of enviromental enrichment. Nat Rev Neurosci 2000 13. 2000;1(3):191–8.

9. Morandi-Raikova A, Mayer U. Spatial cognition and the avian hippocampus: Research

in domestic chicks. Front Psychol. 2022 Sep 23;13:5791.

10. Rosa-Salva O, Mayer U, Versace E, Hébert M, Lemaire BS, Vallortigara G. Sensitive periods for social development: Interactions between predisposed and learned mechanisms. Cognition. 2021 Aug 1;213:104552.

11. Della Chiesa A, Pecchia T, Tommasi L, Vallortigara G. Multiple landmarks, the encoding of environmental geometry and the spatial logics of a dual brain. Anim Cogn. 2006 Oct 29;9(4):281–93.

12. Vallortigara G. Learning of colour and position cues in domestic chicks: Males are better at position, females at colour. Behav Processes. 1996 Jun 1;36(3):289–96.

13. Rashid N, Andrew RJ. Right hemisphere advantage for topographical orientation in the domestic chick. Neuropsychologia. 1989 Jan 1;27(7):937–48.

14. Morandi-Raikova A, Vallortigara G, Mayer U. The use of spatial and local cues for orientation in domestic chicks (Gallus gallus). Anim Cogn 2020 232. 2020 Jan 1;23(2):367–87.

15. Gunnarsson S, Yngvesson J, Keeling LJ, Forkman B. Rearing without early access to perches impairs the spatial skills of laying hens. Appl Anim Behav Sci. 2000 Apr 3;67(3):217–28.

16. Freire R, Cheng HW, Nicol CJ. Development of spatial memory in occlusion-experienced domestic chicks. Anim Behav. 2004 Jan 1;67(1):141–50.

17. Freire R, Cheng HW. Experience-dependent changes in the hippocampus of domestic chicks: a model for spatial memory. Eur J Neurosci. 2004 Aug 1;20(4):1065–8.

18. Ferreira VHB, Guesdon V, Calandreau L. How can the research on chicken cognition improve chicken welfare: a perspective review.

https://doi.org/101080/0043933920211924920. 2021;77(3):679–98.

19. Widowski T, Torrey S. Rearing young birds for adaptability. Adv Poult Welf. 2018 Jan 1;49–76.

20. Leenstra F, Ten Napel J, Visscher J, Van Sambeek F. Layer breeding programmes in changing production environments: a historic perspective. Worlds Poult Sci J. 2016 Mar 1;72(1):21–36.

21. Schuck-Paim C, Negro-Calduch E, Alonso WJ. Laying hen mortality in different indoor housing systems: a meta-analysis of data from commercial farms in 16 countries. Sci Reports 2021 111. 2021 Feb 4;11(1):1–13.

22. Rentsch AK, Harlander A, Niel L, Siegford JM, Widowski TM. Rearing laying hens: Environmental complexity and genetic strain affect pullet but not chick performance in a T-maze learning task. Appl Anim Behav Sci. 2023;265:105997.

23. Tahamtani FM, Nordgreen J, Nordquist RE, Janczak AM. Early life in a barren environment adversely affects spatial cognition in laying hens (Gallus gallus domesticus). Front Vet Sci. 2015 Mar 18;2(MAR).

24. Muir GD, Gosline JM, Steeves JD. Ontogeny of bipedal locomotion: walking and running in the chick. J Physiol. 1996 Jun 1;493(2):589–601.

25. Muir GD. Early ontogeny of locomotor behaviour: a comparison between altricial and precocial animals. Brain Res Bull. 2000 Nov 15;53(5):719–26.

26. Rentsch AK, Ross E, Harlander A, Niel L, Siegford JM, Widowski TM. The development of laying hen locomotion in 3D space is affected by early environmental complexity and genetic strain. Sci Rep. 2023;13(1):10084.

27. Thiele H-H. Management recommendations for rearing pullets for alternative housing

systems. Lohmann Inf. 2007;42:14–24.

28. Stratmann A, Guggisberg D, Benavides-Reyes C, Siegford J, Toscano MJ. Providing ramps in rearing aviaries affects laying pullet distribution, behavior and bone properties. J Appl Poult Res. 2022 Dec 1;31(4):100283.

29. Norman KI, Weeks CA, Tarlton JF, Nicol CJ. Rearing experience with ramps improves specific learning and behaviour and welfare on a commercial laying farm. Sci Rep. 2021 Apr 23;11(1):1–14.

30. Johny A, Guggisberg D, Toscano MJ, Stratmann A. This is the way: The effect of artificial cues on early life ramp use behaviour of laying hen chicks. Appl Anim Behav Sci. 2023 Mar 1;260:105873.

31. Van Praag H, Christie BR, Sejnowski TJ, Gage FH. Running enhances neurogenesis, learning, and long-term potentiation in mice. Proc Natl Acad Sci U S A. 1999 Nov 9;96(23):13427–31.

32. Van Praag H. Neurogenesis and exercise: Past and future directions. NeuroMolecular Med. 2008 Jun 20;10(2):128–40.

33. Merkley CM, Jian C, Mosa A, Tan YF, Wojtowicz MM. Homeostatic regulation of adult hippocampal neurogenesis in aging rats: long-term effects of early exercise. Front Neurosci. 2014;8(8 JUL).

34. Hillman CH, Erickson KI, Kramer AF. Be smart, exercise your heart: exercise effects on brain and cognition. Nat Rev Neurosci 2008 91. 2008 Jan;9(1):58–65.

35. Sibley BA, Etnier JL. The Relationship between Physical Activity and Cognition in Children: A Meta-Analysis. Pediatr Exerc Sci. 2003 Aug 1;15(3):243–56.

36. Rossi GS, Wright PA. Does leaving water make fish smarter? Terrestrial exposure and

exercise improve spatial learning in an amphibious fish. Proc R Soc B. 2021 Jun 30;288(1953).

37. Mes D, Palstra AP, Henkel C V., Mayer I, Vindas MA. Swimming exercise enhances brain plasticity in fish. R Soc Open Sci. 2020 Jan 1;7(1).

38. Fiaz AW, Léon-Kloosterziel KM, Leeuwen JL van, Kranenbarg S. Exploring the effect of exercise on the transcriptome of zebrafish larvae (Danio rerio). J Appl Ichthyol. 2014 Aug 1;30(4):728–39.

39. Boogert NJ, Madden JR, Morand-Ferron J, Thornton A. Measuring and understanding individual differences in cognition. Philos Trans R Soc B Biol Sci. 2018 Sep 26;373(1756).

40. Shaw RC, Schmelz M. Cognitive test batteries in animal cognition research: evaluating the past, present and future of comparative psychometrics. Anim Cogn. 2017 Nov 1;20(6):1003–18.

41. Rufener C, Berezowski J, Maximiano Sousa F, Abreu Y, Asher L, Toscano MJ. Finding hens in a haystack: Consistency of movement patterns within and across individual laying hens maintained in large groups. Sci Rep. 2018 Dec 1;8(1).

42. Gómez Y, Berezowski J, Jorge YA, Gebhardt-Henrich SG, Vögeli S, Stratmann A, et al. Similarity in Temporal Movement Patterns in Laying Hens Increases with Time and Social Association. Animals. 2022 Mar 1;12(5):555.

43. de Haas EN, Lee C, Hernandez CE, Naguib M, Rodenburg TB. Individual differences in personality in laying hens are related to learning a colour cue association. Behav Processes. 2017 Jan 1;134:37–42.

44. Zidar J, Balogh A, Favati A, Jensen P, Leimar O, Sorato E, et al. The relationship

between learning speed and personality is age- and task-dependent in red junglefowl. Behav Ecol Sociobiol. 2018 Oct 1;72(10):1–13.

45. Nilsson J-Å, Brönmark C, Hansson L-A, Chapman BB. Individuality in movement: the role of animal personality. Anim Mov across scales. 2014;1:90–109.

46. Spiegel O, Leu ST, Bull CM, Sih A. What's your move? Movement as a link between personality and spatial dynamics in animal populations. Ecol Lett. 2017 Jan 1;20(1):3–18.

47. Armstrong EA, Voelkl B, Voegeli S, Gebhardt-Henrich SG, Guy JH, Sandilands V, et al. Cell Proliferation in the Adult Chicken Hippocampus Correlates With Individual Differences in Time Spent in Outdoor Areas and Tonic Immobility. Front Vet Sci. 2020 Aug 26;7:587.

48. van der Staay FJ, Gieling ET, Pinzón NE, Nordquist RE, Ohl F. The appetitively motivated "cognitive" holeboard: A family of complex spatial discrimination tasks for assessing learning and memory. Neurosci Biobehav Rev. 2012 Jan 1;36(1):379–403.

49. Nordquist RE, Heerkens JLT, Rodenburg TB, Boks S, Ellen ED, van der Staay FJ. Laying hens selected for low mortality: Behaviour in tests of fearfulness, anxiety and cognition. Appl Anim Behav Sci. 2011 May 1;131(3–4):110–22.

50. Dumontier L, Janczak AM, Smulders T V., Nordgreen J. Effects of the rearing environment complexity on laying hens' spatial cognition: A holeboard test approach. Appl Anim Behav Sci. 2023 Mar 1;260:105878.

51. Bates D, Mächler M, Bolker BM, Walker SC. Fitting Linear Mixed-Effects Models Using lme4. J Stat Softw. 2015 Oct 7;67(1):1–48.

52. Brooks ME, Kristensen K, van Benthem KJ, Magnusson A, Berg CW, Nielsen A, et al.

glmmTMB balances speed and flexibility among packages for zero-inflated generalized linear mixed modeling. R J. 2017 Dec 1;9(2):378–400.

53. Hartig F. DHARMa: Residual Diagnostics for Hierarchical (Multi-Level / Mixed) Regression Models. R package version 0.2.0. https://CRANR-project.org/package=DHARMa. 2020;

54. Halekoh U, Højsgaard S. A Kenward-Roger Approximation and Parametric Bootstrap Methods for Tests in Linear Mixed Models – The R Package pbkrtest. J Stat Softw. 2014 Sep 12;59(9):1–32.

55. Bolker B, Robinson D. Broom. mixed: Tidying methods for mixed models. R Packag version 02. 2020;6.

56. Lenth R, Singmann H, Love J, Buerkner P, Herve M. Package 'emmeans'. 2019.

57. Berner D, Amrhein V. Why and how we should join the shift from significance testing to estimation. J Evol Biol. 2022;35(6):777–87.

58. Wickham H, Averick M, Bryan J, Chang W, D' L, Mcgowan A, et al. Welcome to the Tidyverse. J Open Source Softw. 2019 Nov 21;4(43):1686.

59. Wickham H, Chang W. Package ggplot2: An Implementation of the Grammar of Graphics. Creat elegant data Vis using Gramm Graph. 2016;v. 2(1):1–189.

60. Shaw AK. Causes and consequences of individual variation in animal movement. Mov Ecol 2020 81. 2020 Feb 17;8(1):1–12.

61. Schirmer A, Herde A, Eccard JA, Dammhahn M. Individuals in space: personality-dependent space use, movement and microhabitat use facilitate individual spatial niche specialization. Oecologia. 2019;189:647–60.

62. Fraser DF, Gilliam JF, Daley MJ, Le AN, Skalski GT. Explaining Leptokurtic Movement Distributions: Intrapopulation Variation in Boldness and Exploration. Am Nat. 2001;158(2):124–35.

63. Dingemanse NJ, Both C, Van Noordwijk AJ, Rutten AL, Drent PJ. Natal dispersal and personalities in great tits (Parus major). Proc R Soc London Ser B Biol Sci. 2003;270(1516):741–7.

64. Kolakshyapati M, Taylor PS, Hamlin A, Sibanda TZ, Vilela J de S, Ruhnke I. Frequent Visits to an Outdoor Range and Lower Areas of an Aviary System Is Related to Curiosity in Commercial Free-Range Laying Hens. Anim 2020, Vol 10, Page 1706. 2020 Sep 21;10(9):1706.

65. Campbell DLM, Hinch GN, Downing JA, Lee C. Fear and coping styles of outdoor-preferring, moderate-outdoor and indoor-preferring free-range laying hens. Appl Anim Behav Sci. 2016 Dec 1;185:73–7.

66. Sih A, Del Giudice M. Linking behavioural syndromes and cognition: a behavioural ecology perspective. Philos Trans R Soc B Biol Sci. 2012;367(1603):2762–72.

67. Pearce JM, Bouton ME. Theories of Associative Learning in Animals. https://doi.org/101146/annurev.psych521111. 2003 Nov 28;52:111–39.

68. Rajalakshmi R, Jeeves MA. The relative difficulty of reversal learning (reversal index) as a basis of behavioural comparisons. Anim Behav. 1965 Apr 1;13(2–3):203–11.

69. Dougherty LR, Guillette LM. Linking personality and cognition: a meta-analysis. Philos Trans R Soc B Biol Sci. 2018 Sep 26;373(1756).

70. Roelofs S, Murphy E, Ni H, Gieling E, Nordquist RE, van der Staay FJ. Judgement bias in pigs is independent of performance in a spatial holeboard task and conditional

discrimination learning. Anim Cogn. 2017;20:739–53.

71. Jones CT, Pullin AN, Blatchford RA, Makagon MM, Horback K. Effects of rearing with vertical structures on the ontogeny of depth perception in laying hens. Appl Anim Behav Sci. 2023 Feb 1;259:105837.

Response to the reviewers for PONE-D-23-28746 titled "Mind the ramp: Association between early life ramp use and spatial cognition in laying hen pullets.

Editor:

Discussion of the results requires more depth in interpreting the results.

We have added new portions (L 628 - 640), removed certain sections (L 611 – 627, 642- 666) and rewritten (L 567 - 610) to bring more depth in interpreting results as well as make the discussion succinct.

The statistical model needs clarification.

We used a combination of estimates, confidence intervals and p values to describe relationship with response and explanatory variables as described in L341 - 342., rather than use p < 0.05 as cut-off. We also wanted to try the approach of providing as many results as possible without the use of terms such as statistically significant and non-significant as advocated by Wasserstein et al., 2019, Hulbert et al., 2019, Berner and Amrhein (2022) etc. We followed a similar method as Rosenberger et al. (2022) to report our results.

The entire manuscript needs a brief, more in-depth and clearer writing, with recent references cited.

We have removed and rephrased parts in all the sections to improve the clarity as well reduce the length of the manuscript. We have also added more references to support out claims.

Reviewer #1:

This study tested the association between spatial memory and learning (holeboard test) and voluntary ramp use in laying hen pullets. They found no within-bird consistency for ramp use behaviour. They did find relationships between ramp use frequency and the performance in the holeboard test, some of them supporting their predictions and some to the contrary. The experimental design is sound, though the statistical analyses are perhaps unnecessarily complicated. If no statistician was involved, I would recommend that the authors consult a statistician. If there was a statistician involved, then the section on statistics needs a bit of clarification. The manuscript is well written though it is

lengthy and unnecessarily wordy and repetitious at times. My biggest concern is the discussion. There appears to be a lack of references and the structure can be improved by reducing redundant elaborations. Once cleaned up, this manuscript can be a valuable contribution to the laying hen literature.

Thanks for the detailed review and valuable feedback. After talking to a researcher with statistical expertise we have decided to remove the repeatability analysist because the sample size is on the lower end to calculate repeatability of ramp use. We have hence removed parts pertaining to repeatability analysis in all section of the manuscript. We also reanalysed the phase change analysis to include mean ramp use frequency (MRF) as a continuous variable. This analysis included all the birds unlike the previous analysis which used ramp use a categorical variable with high (ten birds with highest MRF) and low ramp users (ten birds with lowest MRF).

Major Comments

Stay consistent with the numbering (spelling out numbers under 10), please check the whole manuscript for inconsistencies.

Thanks. We have changed the numbering accordingly.

Abstract

You need to introduce terminology somewhere: chicks vs hens vs pullets. This journal is not poultry-specific.

We don't think it is necessary. They are universally used terms and not specific to the paper and can be easily obtained if the reader does a quick web search.

L27: what analyses? Give a model or analysis type to help understand the results.

We have changed it to Mixed-model analysis.

L30-33: These results are a bit confusing. First, they are trends/tendencies and not significant results, so you should state that. Second, it is unclear what the unit of those estimates are.

*The estimates are back transformed, so it is integer scale (number of errors were positive integers including zero). The estimate here is a slope. For example, a 0.94 estimate means for one unit of increase in ramp use there is a 6% decrease in errors made. The change in errors between x and x+n ramp use would be n*0.6.*

For interpretation of statistics, we did not use p < 0.05 as cut-off. Rather we used a combination of estimates, confidence intervals and p values to describe relationship with variables as described in L341 - 342. We also wanted to try the approach of providing as many results as possible without the use of terms such as statistically significant and non-significant as advocated by [Wasserstein et al., 2019](), [Hulbert et al., 2019]() etc.,

L37: Please state why this would be relevant/ interesting.

With the given word limit, it is unfortunately not possible to have another sentence in the abstract, without losing information that we think are critical. Moreover, the relevance of the topic is covered in the introduction part.

Introduction

L63: I would suggest exchanging the Campbell reference with Widowski and Torrey's book chapter on rearing young birds for adaptability: https://linkinghub.elsevier.com/retrieve/pii/B9780081009154000038. This statement refers to a shift in research interest based on the shift in management. Both the Ferreira and Widowski and Torrey references are revisions of the literature and the importance of research in this area. The Campbell paper is an experiment that can be mentioned later when discussing specific findings.

Thank you for the suggestion. We have changed it accordingly.

L108-116: This has to go before the research question/objectives (L106-107). Then, add lines 106-107 to the beginning of the next paragraph that contains your predictions.

Thanks for the feedback. We have rearranged the sentences and rephrased the research objective (L125 - 128)

Methods

L130-131: It's a bit confusing to point to your other paper for the pen schematics. I immediately looked at your Figure 1 and expected to see a pen. I would suggest you provide schematics or a photo here. Alternatively, add the reference after your description and say that more information and schematics can be found in Johny et al. (28). But I would prefer a photo or schematics in this paper.

We have added the sentence you suggested in L 153. Thank you.

L157: How did you choose your random sample? How did you make sure there was no bias (e.g., first catches could be bold, last in the box could be most fearful).

Its not a random sample. Chicks were arbitrarily assigned to the pens. At the time of population, they came in crates. We picked the birds from the crates arbitrarily, applied colour marks and then introduced them to the pens. Since they were all constrained in small crates which did not allow any movement, we think there were no bias due to the catch order.

L169-173: Unclear what you mean here. What treatments? How were they managed? Why is this important?

The treatments were three different artificial cues that were aimed at improving ramp use and a control with no cue that were part of an additional experiment (Johny et al., 2023). The treatment groups influenced the variation in use of ramps, in addition to the individual-level differences. So, we added the treatment as control variable to account for the variation in ramp use due to the cues. We have added this information in L186 – 191.

L292-295: This section needs clarification. I do not understand it. Do you mean the slopes in your statistical model? I also don't understand why you group the birds into high and low ramp users. I though your sample was random. Did you have two clusters? Elaborate on this and on why you chose to create these categories instead of using their actual frequencies.

We changed the analysis and included MRF as a continuous variable like the rest of the analysis. This allowed us to use all the birds unlike the previous analysis which included ramp use as categorical variable with high (ten birds with highest MRF) and low ramp users (ten birds with lowest MRF).

L306-308: Why did you use bootstrapping to get the main effect p-values? To the best of my knowledge, this method is used to reduce a model and get p-values for reduced factors only. The main effects can be assessed as part of the model. Was a statistician involved in this analysis? If so, I will retract my comment. Otherwise, I would like you to consult a statistician.

We used the method suggested by PD Dr. Lorenz Gygax, HU Berlin. Although we did not consult him specifically for this experiment, one of the authors took a statistical course from him. We performed the analysis according to the framework provided in the course. Some major additions to the statistics from the course were:

1. *Setting sum contrasts for the categorical variables and standardising continuous variables which provide interpretable main effects, even in the presence of interaction effects. This approach provides parameters such as p-values, estimates and confidence intervals of all main effects without the need to dropping interaction terms or reducing the global model that was initially chosen because of their biological relevance.*
2. *Using bootstrapping to obtain p-values, which are more conservative than likelihood ratio test, due to its robustness in dealing with influential points (Luke, 2016).*

L326: If you only include non-zero observations, are you not biasing the analysis? What if a bird uses it occasionally? As I understand it, you would overestimate the repeatability by only including the observations where the bird happened to use the ramps and excluding observations where it didn't.

We removed the repeatability analysis after consulting a researcher with statistical expertise because our sample size is on the lower limits required for repeatability analysis.

However, to answer your question, we did two different analyses to account for the huge number of zeroes. The first analysis is a binary model and had zeroes included in it. As it was binary, ramp use was coded as if a focal bird used ramps in a particular bout (1) or not (0). In the second analysis, we excluded the observations where an individual birds did not use the ramps. In that case, your argument is right. Taking out zeroes increases the variation in data and since repeatability is the ratio of individual level variance to total variance, one would expect an increase in repeatability. But as was mentioned in the discussion section that is now removed, the total variability of data is less, because of the 3 min observation window we used. That is likely a reason why we still didn't see repeatability in ramp use, even after excluding zeroes.

Results

I recommend making the sentence about the effects of $p < 0.01$ less vague. If you decide to report tendencies (which you need to clarify in the stats section), report them without embellishments. E.g., 'errors tended to decrease with trial number' instead of 'with very limited statistical support, Y made slightly fewer errors'.

As we wrote in response to your comment in the abstract part, we are using the effect size, uncertainty intervals and p-values to describe our results, rather than using the p-value cut-off of <0.05. So, we also opt to change the use of terms such as significant (p<0.05) and trend (0.05<p< ~0.08). We do understand that this is not the norm in the field. We used a similar method as in Rosenberger et. al, 2022, to describe our results.

L385-386: I am still confused about where these high and low classifications come from… and now there is a medium group. Was I supposed to know that? Maybe I missed it.

We wanted to provide the estimated marginal means (EMM) for the number of errors made. We selected birds with low, median, and high MRF as a few examples to provide the EMMs, instead of providing EMMs of all MRF observations. Additionally, we removed the low and high ramp user category from the phase change analysis as mentioned earlier.

L448: I am not sure if removing these data points is appropriate. What if the group you claim learned faster actually had trials in there that were not completed? I recommend analysing it with the complete data set or declaring which ramp use groups the omitted data points came from if they were exclusively from low ramp users.

9, 1 and 7 out of 480, 960 and 370 observations from cued, uncued and reversal phases, respectively were omitted, which we think will have a negligible effect on the analysis.

Discussion

There is a general lack of references supporting sweeping statements in this discussion. Please put your results into the context of existing literature and how your study adds to the knowledge base. There is also a lot of repetition that could be cut down (e.g., repeating of your own results in every paragraph).

Thanks for the feedback. We have restructured the discussion based on your comments.

L497-505: But your birds' ramp use behaviour was inconsistent over time. How would that translate to personality, which is what you are implying but not spelling out here.

Our analysis did not reveal consistency in ramp use. But given results from Montalcini et al., 2023a, Montalcini et al., 2022, we would expect this behaviour to be repeatable. Also the movement patterns in aviaries across individuals have been shown to be consistent (Rufener et al., 2023, Montalcini et al., 2023a, Montalcini et al.,2023b). We think it's a limitation of the method (three-minute observation period) as well the low sample size (which led us to remove the analysis on repeatability of ramp use) which rendered the ramp use behaviour non-repeatable in our analysis.

The argument we make is that differences in ramp use (a movement pattern) and spatial cognition could be driven by individual differences in activity, exploration, and fearfulness. The differences in movement behaviours have been shown to correlate to differences in behaviours such as activity, exploration as mentioned in L533-538. On that note, it is possible that ramp use might also be driven by individual differences in activity (Rufener et al., 2023) and exploration (de Haas et al., 2017; Rodenburg et al., 2003) which has been shown to be repeatable in hens.

L568-572: Why would this only affect the uncued phase? If HR pullets do better in the cued but not the uncued phase, would this mean there is no difference in spatial learning (location) but only in association learning (colour)?

Yes, this is one possible explanation. We have added this in L627- 640. Thanks for the suggestion.

L580-583: WoA 19 is not the start of peak of lay, and WoA25 is not sexual maturity… it would be the reverse. Are you sure these ages and the terminology have the correct references? I also think that these are two different aspects. Sexual maturity would end the ontogenesis and be accompanied by hormonal changes and, in the case of laying hens, also with a change in housing. The peak of lay is well after that and is not initiated by hormonal or housing changes. I would

be hesitant to group those two results and make one statement about behavioural consistency during ontogeny.

As we removed the ramp use repeatability analysis, this part of discussion is no longer in the manuscript.

L590-593: As ramp use was the behaviour in focus, why choose an observation period that you think is too short? If you had good reason to assume it was appropriate before you started, you should not backtrack now but rather accept your results. If you have reason to believe it was inappropriate based on new information after the fact, please add a section with limitations and explain your initial rationale and what changed.

This part is removed from the manuscript as it part of the repeatability analysis.

L593-596: No. You tested a hypothesis and did not reject the null hypothesis. If you have reason to assume a type 2 error, you must provide it. Otherwise, you cannot state your initial prediction after not confirming it. Also, what is 'short-term' consistency even? If you introduce a vague term like that, you have to define it and back it up with literature.

This part is removed from the manuscript as it part of the repeatability analysis.

Minor Comments

Do not be alarmed by the number of comments, most are very small and should be easily addressed.

Abstract

L19: 'affect the brain' is very vague. Be more specific or don't mention the brain.

Removed the brain part.

L25: 'observation period' what ages are those? Instead of observation period. Give an age range.

Added the observation days.

L25-26: 'spatial cognition' be more specific on what aspect of spatial cognition this test is assessing.

Changed it to long and short-term spatial memories.

L26: The verb refers to spatial cognition not to the birds. Change 'were' to 'was'.

Now it is '*long and short-term spatial memories' instead of spatial cognition.*

L29: 'averaged across all observation days' is redundant as you already stated that you analyzed the means.

Removed 'mean'.

L30: spell out Est and CI when you first use it.

Done.

Introduction

L67-68: I don't mean to increase my own citation but there is newer literature on this which also happened to be mine… I'm not telling you to include it, but you might want to consider it. See https://doi.org/10.1016/j.applanim.2023.105997

Added the citation.

L69-70: Same comment as above. Consider including this reference as it is more recent: https://doi.org/10.1038/s41598-023-35956-1

Added the citation.

L88-91: This sentence is too long and complex and I am not sure what the content is. Not only do they use ramps to transition between tiers but also to… what?

Rephrased the sentence for clarity.

L96: The 'inter' part of inter-individual differences is redundant as there can be no intra-individual difference in cognition as far as I'm aware (unless you do a before/after thing). Adding the 'inter' suffix draws attention and seems unnecessarily confusing.

There are intra-individual differences in cognition. And inter- individual differences is a commonly used term in field of behavioural sciences. So, we don't think it is unnecessarily confusing.

L96-97: This sentence is a bit awkward. I would suggest rewording it. Here are two suggestions, but it's obviously up to you. 'There has been a recent peak in the interest in individual cognitive differences and their importance in the study of cognition.' OR 'Individual cognitive differences and their importance in the study of cognition have received a recent increase in interest/attention.'

Rephrased using your second suggestion.

L97-98: Add a comma after surfaces

We have rephrased the sentence now.

L99-100: Personally, I would say locomotion instead of movement behaviour, but that's just a suggestion. Or are you talking about types of locomotion?

We are not talking about types/modes of locomotion, such walking or running, but rather about patterns of movement such as range use and space use within the aviary. We think movement behaviour captures this more than locomotion, hence we prefer to use movement behaviour.

L99-101: 'individual differences' three times in one sentence. I would change the last one to say 'can lead to differences in developmental trajectories'. The whole sentence is a bit vague. I would say 'results in' instead of 'can result in' as the start of the sentence already states that this is a possibility and not a fact.

Rephrased for clarity.

L103: 'Hence, it is important…', but that might be a personal preference.

Changed accordingly.

L104: Again, just say individual differences, not individual-level differences (redundant). Or rephrase to say 'differences at the individual level'.

Rephrased accordingly.

L106: The 'inter' part of 'inter-individual variation' is redundant.

We disagree for the reason explained earlier.

L107: Here too, I find that spatial cognition is too broad a term for a holeboard test. Say what aspect of spatial cognition you were testing.

Rephrased as spatial memories.

L117-120: this sentence is too long. Break this into two or even three sentences.

The sentence was removed.

Methods

L129-130: Either say ‘All pens housed 22 birds’ or ‘Pens housed 22 birds each’. Using ‘all’ and ‘each’ is too unnecessary.

Thanks. Rephrased according to your suggestion.

L136-137: half of this sentence is redundant. The pen floors were covered in wood shavings (dimensions). OR The pen floors were littered with wood shavings (dimensions).

Done.

L138: plastic barriers made of metal sheets? Generally, that sentence is a bit wordy. Simplify

Rephrased the sentence and split into two for clarity.

L141: why were they confined for the first 4 days? I know why, but you need to spell it out (briefly!) for non-poultry people.

Yes, thank you. Added one sentence as explanation in L155 - 156.

L142: What do you mean by feeder plate? Like a flat dish? Can you give a description?

Added dimensions.

L147: It would be nice to have the LUX. I’m assuming it changed with age, so you could add it to the sentence where you give the change in light period length.

Added the information in L163.

L151: Wordy -> five minutes at dawn and 30 minutes at dusk.

Thanks. Changed according to suggestion.

L151: Why say that daylight was blocked? It would suffice to say there was none. Or even better, add ‘there was no natural light’ to your lighting sentence.

Deleted the line ‘daylight was blocked’ and added ‘there was no natural light’ in L 164.

L167: How do you know there was no aggression? Anecdotal observations? Injuries?

We did not observe any aggression towards colour marked birds during the video analysis and animal checks during management.

L178-179: The software ‘recorded the behaviour’? Did it code it? Did it record locations? How? Did it record birds, and you recorded the behaviour?

The software recorded the videos, and we analysed the videos. Changed wording to clarify.

L180-186: There are many repetitions here and it’s wordier than necessary. I suggest rewriting this passage, making it more concise. E.g., “…: the inter-tier transitions (up and down combined) and active behaviours on the ramp (walking, running, wing-assisted incline running, jumping to or from the ramp without changing tier; see ethogram in Johny et al (28)).

Reworded to make it concise.

L207-208: Hence, birds were visually but not auditorily isolated.

Thanks, changed as per suggestion.

L209-211: Don’t repeat the information that is in the figure. The arena held eight circles containing small cups to provide the reward (grape pieces).

We removed most of the measurements, but retained the previous text as we think it is a good description of the arena.

Figure 1: It might help to visualize that 1B represents half of 1A. E.g., you could draw lines from the corners of one pen to the corners of the arena.

We added more information in the figure description. We think drawing the lines from corners of pen might render the figure more cluttered.

L220 ff: This should go into the paragraph ‘Cue phase trial’. Continuing to habituation from here seems like a step back. I suggest you rename this paragraph to ‘Arenas’ and only include the arena description. It would make more sense to go from ‘Arenas’-> ‘Habituation’ -> testing procedure.

The information given is important to make sense of the habituation and test phase. For ex., “the birds were habituated and tested in the same arena”.

The paragraph contains certain procedures that were common for the habituation and the test phases. So, we prefer to keep it as ‘Arena and procedure’.

L234: Did you ensure that your focal birds had eaten the grapes during the habituation period?

Yes, the information is given L 257 – 258.

L234: How did you choose the focal birds? Convenience sampling?

Changed the wording from ‘randomly chosen’ to ‘arbitrarily chosen’. So, it is convenience sampling.

L254: Did you use a learning criterion, or did all birds eat all grapes in all trials?

There was no learning criterion in the cued phase. All birds did not eat all grapes in all trials. All birds ate from all rewarded cups in almost all trials. However, calculation of the memory errors doesn’t require birds to visit all the rewarded cups.

L259: I suggest you move the information on the learning criterion of the uncued phase into the section about the uncued phase. Also, if birds reached the criterion early, did they still do all 20 trials?

The learning criterion was used only for the selecting birds for the reversal phase and we did not use the learning criterion for any analysis including the uncued phase. So, this information is only relevant for the reversal phase and thus we prefer to keep it in the ‘reversal’ section.

All the birds had 20 trials, no matter when they reached the learning criterion.

L283: what is the unit here?

Seconds. Added the information.

L288 ff: To delete the very long and complicated sentence on L288-292, you could include the number of trials like so: "… , we compared errors of each bird from the three trials before and three trials after a phase change …" This is only a suggestion.

Thanks for the suggestion. Changed accordingly.

L302-305: Out of curiosity, what was wrong with your main effects before? I have not seen this to be necessary before, does it not over-specify your models?

Centring the data to zero or using sum-contrasts does not affect fit of the model. The aim of performing this step was to calculate effect sizes and p-values while retaining the interaction term in the model (Schad et al., 2019). We did not drop the interaction term if it was insignificant, as we wanted to stick with our global model.

L332: what treatments? You cannot expect people to read other papers to understand this one.

We have added the information about treatments in L 187 – 191.

Results

Furthermore, many repetitions can be avoided by adding units (count/3min). The reader can be trusted to remember that there were 8 bouts per 7 observation days after you mentioned it once.

L365-368: Repetitions and inconsistencies. You are giving the mean, min, and max count/3min for the individual observations but the mean you call a frequency and the min and max you don't specify. This could be more concise and clarified. Also, you repeat '8 bouts per 7 observations days' which is unnecessary. Furthermore, you are inconsistent with the numbering. It makes the passage more complicated and harder to understand than need be.

Rephrased the sentences. Moreover, the paragraph is simpler now because we removed the repeatability analysis.

L388: I suggest you delete the word 'seem'. It either influenced it or not. You can even say you didn't detect an effect of… but be less vague.

Thanks, we deleted 'seem'.

Figures 2-3: I recommend only showing the statistically significant results (Figure 3); the others are sufficiently described in the text (also for Figure 4). If you choose to show all plots, I recommend merging 2 and 3 into one plot with two panels, as the caption is the same.

This is an exploratory study where we are describing the results. Moreover, as mentioned in the methods section, we have refrained from the use of just p-value based inference of results.

Figures 2-4: I am confused about the colour changes. The grading is according to the Y-axis and does not add information. It made me think there were different treatments.

There is a description of the colour gradient in the figure caption. So, we don't think it's confusing.

L459-460: You have been using HR and LR for a long time at this point; no sense in spelling it out in this bracket. The bracket might be superfluous anyway, especially if you added a section explaining how you defined these groups, as I recommended earlier.

We have removed the HR and LR groups in the analysis.

Table 1: Please add indicators for significant differences (e.g., superscript letters).

We did not perform post-hoc tests as mentioned in L345-346.

Discussion

L478: specify the aspect of spatial cognition you assessed.

Yes, we have changed it to long and short-term spatial memories.

L479: specify the early life period. If you refer to the first eight weeks, move it up from the end of the sentence.

Early life period is now specified.

L481-488: Add which results supported your hypotheses and which didn't.

As this was an exploratory study, we did not have any hypotheses. We removed the expectation based on current literature that we had mentioned in L 128- 132, to avoid any confusion.

L491: Add reference supporting your statements.

This sentence has been removed.

L518-530: Most of this is not new information. This whole paragraph seems redundant. Add the main points to the last paragraph where needed and delete this paragraph. There is also a lack of references backing your statements.

Thanks for your suggestion. We have combined both paragraphs in L566 - 580.

L587-590: The cattle example seems out of place. I recommend you remove it.

Removed.

L597-614: This paragraph seems out of place and irrelevant. It adds no new information and does not support your suggested interpretations. I recommend deleting it as it dilutes your discussion.

As holeboard is not yet widely used to measure spatial cognition we wanted to compare the scores from few other studies that have used holeboard test in hens for external validation as mentioned in L667-669. Hence we prefer to keep this paragraph.

L615-620: This would be very interesting sections on limitations. However, I am missing a discussion on the consequences of these limitations. How could these limitations affect your results and interpretation?

L627-629: Add references to all of these statements.

We have added the references wherever applicable.

L634-636: That was done here: https://doi.org/10.1016/j.applanim.2023.105997 and to a degree here: https://doi.org/10.1038/s41598-023-35956-1.

Agree. But it is still critical to have more studies on the early rearing complexity and its impact on spatial skills. Also, it might also be interesting to test the effect of conditions that are more complex than the existing aviary setups, to understand if

the current rearing aviaries are complex enough to trigger major developmental changes.

Reviewer #2:

Consideration of welfare in laying hens is good area of research, specially when battery cages are not much in use in Europe. Overall, this is a well written paper, however, it needs some revisions. Some of these are mentioned in the attached file.

Thank you for your suggestions.

In methodolgy, no. Of birds is 351; suggesting one pen had 21 birds.

Thank you. One pen only had 21 pens from the beginning. Since this information is not very relevant, we have removed it from abstract. The animal numbers are explained in detail in the methods section.

Abstract should be self explanaitry. There should be some detail of these phases.

For the given word limit, adding details on the phases is not possible without dropping other relevant information. So, we choose not to add more details on the phases.

If there was a mortality, then please elaborate it as well.

There was no mortality. The reason for 21 chicks is now added in L 146- 147.
