# Supplementary material for: Mind the ramp: Association between early life ramp use and spatial cognition in laying hen pullets
Source: PLoS One. 2024 Apr 26;19(4):e0302454. doi: 10.1371/journal.pone.0302454 (PMC11051627; doi:10.1371/journal.pone.0302454)
Supplement: Attachment — Submitted filename: Johny et.al.2023.docx [file pone.0302454.s001.docx]

**Mind the ramp: Association between early life ramp use and spatial cognition in laying hen pullets.**

Alex Johny^1, 2*^, Andrew M. Janczak^3^, Janicke Nordgreen^4^, Michael J. Toscano^1^, Ariane Stratmann^1, #^

^1^Centre for Proper Housing of Poultry and Rabbits, VPHI Institute, University of Bern, Zollikofen, Switzerland

^2^Graduate school of Cellular and Biomedical Sciences, University of Bern, Bern, 3012 Switzerland

^3^ Department of Production Animal Clinical Science, Faculty of Veterinary Medicine, Norwegian University of Life Sciences (NMBU), Ås, Norway

^4^ Department of Paraclinical Sciences, Faculty of Veterinary Medicine, Norwegian University of Life Sciences, Ås, Norway

^#^Current Address: Centre for Proper Housing of Ruminants and Pigs, Federal Food Safety and Veterinary Office, Agroscope, Posieux, Switzerland.

*Corresponding author

Email: alex.johny@unibe.ch

**Abstract**

Ramps facilitate earlier access to complex environments and increase early life voluntary exercise, which may positively affect the brain and cognitive development of chickens. This study focused on quantifying individual differences in ramp use and its impact on spatial cognition of laying hen pullets. We used 16 identical pens holding 22 Lohmann Selected Leghorn (LSL) chicks of which eight chicks from each pen were colour marked from one day of age (DoA) to serve as focal birds. We quantified overall ramp use (walk/run, wing-assisted incline running, and jump/fly to and from ramps) by scan sampling recorded videos for seven days distributed across the observation period for all focal birds. From 56 to 95 DoA, spatial cognition of three focal birds per pen were assessed in a holeboard test in three consecutive phases: cued, uncued and reversal. Our analysis showed no consistency in average ramp use per day for individual birds. However, we found that the spatial cognitive abilities of the birds were linked to differences in mean ramp use frequency averaged across all observation days. Birds with higher ramp use made fewer reference (Est ± CI = 0.94 [0.88, 0.99], p = 0.08) and working memory errors (Est ± CI = 0.77 [0.59, 1.00], p = 0.06) in the cued phase than birds with lower ramp use. In contrast, birds with higher ramp use made more reference memory errors (Est ± CI = 1.10 [1.01, 1.20], p = 0.05) in the reversal phase. Birds with higher ramp use also made more reference memory errors compared to birds with lower ramp use as the phases changed from both cued to uncued (p = 0.007) and uncued to reversal (p = 0.02). Our results indicate that there might be a relationship between early life ramp use and spatial cognition of laying hens.

**Key words: chicken, rearing environment, habitat complexity, behaviour, holeboard test**

**1. Introduction**

Spatial cognition is the ability of animals to acquire, use, recall and revise information concerning the spatial layout of their environment (1). It is critical for an animal's survival, as it enables navigation through the environment, locating resources such as food, water, shelter, conspecifics, and avoiding predators. Early life experiences play a huge role in the development of spatial cognitive abilities of animals (2,3) and the complexity of the habitat in this period is a defining feature. Rearing animals in complex environments has been shown to improve spatial learning and memory (4,5), as well as have positive developmental effects on the brain regions involved in spatial cognition in a range of taxa (rat: (6), atlantic salmon: (7), mice: (8)).

The domestic hen is an excellent model species to study the development of spatial cognitive abilities due to their precocial nature (9). Precocial birds can be tested for cognitive functions from a very early age as they hatch with a fully developed sensory-motor system and rapid learning mechanisms (10). Hens are able to navigate complex environments using landmarks (11), egocentric information (12) as well as topographical features of the environment (13). They are also able to use multiple landmarks for orientation, possibly by forming cognitive maps (14). As in several other species, the development of spatial cognition in domestic chicks has also been shown to be influenced by early life environment. Chicks reared in complex environments by either equipping the housing system with elevated perches (15) or ground level visual barriers (16) have been shown to improve their spatial skills in navigating vertical spaces later in life (15) and their egocentric orientation (16). The visual barriers also induced changes in morphology of the hippocampus (17), the brain region involved in regulation of spatial navigation and memory (9).

Spatial cognition of domestic hens has been of scientific interest in applied research recently (18,19), owing to the changes in housing conditions over the last three decades (20). An increasing number of laying hens are being housed in cage-free systems such as aviaries (21), which are structurally complex compared to cage systems (both battery and enriched) due to the vertically stacked tiers, within which essential resources such as perches, litter, food and water are located. Rearing birds in complex aviaries has been shown to benefit the spatial working memory of laying hens (22). However, the access to the three-dimensional environment is compromised during the early life period of the laying hen chicks, as they lack the physical and motor skills to access the elevated surfaces of the aviary (23,24). Producers also generally confine the chicks to the lowest aviary tier for the first two to four weeks of age (WoA), as they want the chicks to stay near food, water, and heat sources, thereby restricting the access to complex environments in their early life (25). To facilitate access to elevated surfaces in the early life of domestic chicks, studies have investigated the provision of ramps, which provide a continuous path connecting the different tiers of the aviary (26,27). Providing ramps has been shown to facilitate the chicks’ access to the elevated surfaces from the first WoA in multi-tiered aviaries without any negative consequences on mortality or welfare indicators (26,27).

Facilitating early access to elevated surfaces using ramps might also have positive consequences on the development of spatial cognition of laying hens. Provision of ramps in aviaries might influence the spatial cognition of the birds mainly in two ways. First, ramps provide earlier exposure to vertical complexity. Laying hen chicks reared with ramps have been shown to use elevated surfaces earlier compared to birds reared without ramps (26,27). Early exposure to vertical complexity aided by ramps might provide earlier opportunities for spatial learning, which in turn can influence cognition and neural development. Second, ramps promote early life voluntary exercise in domestic chicks. Chicks perform more vertical movements between the tiers of aviaries when provided with ramps, with most of these movements happening through ramps (26). In addition to ramps being used for inter-tier transitions, chicks also use the ramps actively by walking, running, jumping to and from ramps, and performing wing-assisted inclined running on ramps which does not result in a tier change at least from three days of age (DoA) (28). The increase in early life voluntary exercise has been shown to improve adult hippocampal neurogenesis and spatial memory in rodents (29–31), humans (32,33) and fish (34–36). Although not directly investigated in hens, it is reasonable to assume that early life voluntary exercise facilitated by ramps might positively influence the development of spatial cognition of laying hens as well.

Inter-individual differences in cognition and the importance of accounting for these differences in studies of cognition has piqued interest recently (37,38). Individual hens differ in the use of resources including the elevated surfaces and the vertical movements they perform within the aviary (39,40). It is possible that the individual differences in movement behaviour and activity can result in individual differences in ramp use, which might lead to different individual developmental trajectories for spatial cognition. For example, individual hens differ in the time spent on the free range which has been shown to positively correlate with cell proliferation in the rostral hippocampal sub-region (41). It is hence important to account for individual-level differences in ramp use behaviour to better understand the impact of early life ramp use on the development of spatial cognition of laying hens.

In the current exploratory study, we describe the relationship between the inter-individual variation in ramp use and the spatial cognition of laying hen pullets using a holeboard test. The holeboard test has been used to assess the spatial discrimination learning abilities in various species (42), including domestic hens (22,43). For the test, a subset of all the potential sites is food-rewarded and a bird can visit any of these sites in whatever order it chooses. The holeboard test provides measures for both short and long-term spatial memories. The reference memory is an indicator of long-term memory and holds trial-independent information such as the locations of food rewards and how to access these food rewards. The working memory and general working memory are measures of short-term memory and hold information that is relevant only for a specific trial such as the sites a bird has already visited in that trial.

As early life ramp use might lead to an earlier exposure to a complex habitat and promote early life voluntary exercise, which can positively benefit the development of spatial cognition, we expected that the individuals with higher frequency of ramp use might have better short and long-term memories than individuals with lower ramp use frequency.

**2. Methods**

**2.1. Ethical approval**

The experiment met the federal and cantonal regulations for the ethical treatment of animals involved in research and was approved by the Veterinary Office of the Canton of Bern, Switzerland (BE 106/19).

**2.2. Animals and Housing**

Sixteen custom-built, identical pens (2.00 m × 2.00 m × 2.50 m (L × W × H)) situated at the Aviforum research facility, Zollikofen, Switzerland were used to house a total of 351 Lohmann Selected Leghorn (LSL) chicks from one day of age (DoA) until 17 week of age (WoA). All pens housed 22 birds each, except for one pen which only had 21 chicks. Each pen (schematics of pen provided in Figure 1, Johny et al. (28)) was fitted with two vertically stacked tiers at heights of 0.25 m and 1.20 m from the floor, and two round metal perches (2.00 m × 0.34 cm (L × diameter)) at 0.28 m and 0.55 m above the second tier. The tiers in each pen were connected by two ramps (1.30 m × 0.24 m (L × W), at an angle of 35˚) made of metal grids (0.65 m × 0.25 m (L × W)). Both tiers were made of plastic grids with the first tier (2.00 m × 1.15 m (L × W)) being wider than the second (2.00 m × 0.60 m (L × W)). The pen floors were covered with wood shavings, which served as a litter area (2.00 m × 0.75 m (L × W)). The pens were visually isolated from each other using opaque plastic barriers made of metal sheets that covered up to 1.50 m of the pen walls from the floor and plastic sheets that covered the rest. Olfactory and auditory contact was still possible. Birds were populated on the first tier and confined there using wire mesh until four DoA. Feed was provided *ad libitum* on a feeder plate located on the first tier until two WoA, after which the feed was provided from a dispenser placed in the litter area. An additional feeder plate on the second tier was provided until five WoA. Water was provided *ad libitum* using nipple drinkers on the first tier. The birds were fed with starter feed (Egli Mühlen AG, Nebikon, Switzerland) from one until nine WoA and pullet feed (Egli Mühlen AG, Nebikon, Switzerland) from nine until 17 WoA. A light (Silox basic, 120 – 3000K) attached to each pen ceiling ensured uniform illumination. The light schedule followed standard rearing management for LSL (25) pullets with 24 hours of light for the first two days of life, which was gradually reduced to nine hours in the fifth WoA and stayed the same until the conclusion of the project. The dimming phase of the light lasted for five minutes in the dawn and 30 minutes in the dusk phase. Daylight was blocked to ensure uniform lighting across pens. Birds were vaccinated according to the standard rearing management protocol for LSL birds (Infectious bronchitis-primer, Marek’s disease, Gumboro at one DoA at the hatchery, Paracox at five DoA, Infectious bronchitis 4/91 at 16 DoA, Infectious bronchitis Ma5 at 65 DoA, Avian encephalomyelitis at 93 DoA, Poulvac E.coli at 103 DoA and inactivated Infectious bronchitis vaccine at 114 DoA).

During population at one DoA, eight birds per pen were arbitrarily chosen as focal individuals. Each focal individual was marked with animal marking spray (RAIDEX, Hauptner, Switzerland) and tagged with numbered leg rings that were 5 mm in diameter (Flexi-Ringe, Fieger AG, Switzerland). We used green, black, and brown colours to mark the birds. Green and brown colours were used to mark two focal birds per colour per pen by applying a dot either on the back or neck region. Black colour was most recognizable on the chicks especially at younger ages and was used to mark four focal birds per pen. Four patterns were used with black colour – one dot on neck or back region, one dot on both neck and back, and a continuous line that ran from the neck to back. The colour marks were reapplied at 3, 8, 15, 29 and 43 DoA. The leg rings were changed to 8, 12 and 14 mm in diameter at 8, 29 and 43 DoA, respectively. No aggression towards focal birds was observed and no difference in body mass was found between focal and non-focal birds at 14 WoA.

Birds used in the current experiment were also part of another experiment that investigated artificial cues to encourage early life ramp use in laying hen chicks (28). The experiment that investigated the use of artificial cues consisted of four distinct treatment groups, and the influence of these treatments was carefully managed during the data analysis for the present study.

**2.3. Data Collection**

**2.3.1. Ramp use**

Each pen was fitted with a video camera (Samsung SCO-2080R, IR, Samsung Techwin CO., Korea) connected to customized recording software (Multieye Hybrid Recorder Version 2.3.1.8, Artec Technologies AG, Diepholz, Germany) that recorded the behaviour of birds at regular intervals until nine WoA. We counted the following behaviours for each focal bird per pen (n = 8 birds/pen, total = 128 birds): the number of inter-tier transitions using ramps and the number of active behaviours performed on ramps that did not result in a transition (ethogram described in detail in Table 1, Johny et al., 2023 (28)). An inter-tier transition was defined as the bird moving from one tier to another using a ramp. We did not differentiate between upwards and downwards transitions. An active behaviour performed on ramps was defined as the bird walking, running, performing wing-assisted incline running (WAIR) or jumping to or from the ramps that did not result in a transition (i.e., a change in tiers). We counted active behaviours on ramps as separate events when they were separated by at least five seconds. Both behaviours were counted from recorded videos at 6, 10, 12, 20, 27, 41, and 55 DoA for eight observation bouts of three minutes each dispersed evenly across the light period per day (total observation time per individual per day = 24 minutes).

**2.3.2. Holeboard test**

*General principles*: We used the holeboard test to measure the reference, working and general working memory of the birds in three different phases following initial habituations to a food reward and the test arena. The first phase was cued and served to test the birds’ ability to learn the location of three rewarded cups out of a total of eight cups with colour cues added to the rewarded cups. The second phase was uncued and involved learning the same rewarded locations as in the cued phase, but without the provision of colour cues. In the third, reversal phase, the locations of the rewarded cups were changed, and the birds had to replace the information of location of the rewarded cups they had acquired during the cued and uncued phase with a new one. There were no colour cues associated with rewarded cups in the reversal phase. The birds received 10, 20, and 10 trials each to learn the spatial configuration of the food reward for the cued, uncued, and reversal phases, respectively.

*Arena and procedure*: We tested three focal birds per pen (n = 48) from 56 – 95 DoA in the holeboard test. The arena design and testing methods for the holeboard test were adapted from Dumontier et al. (44). Two arenas were used to test two birds simultaneously which were situated in a separate experimental room adjacent to the barn. The arenas were enclosed by 2 m high walls made of tarpaulin on all sides and had a door on the side (Fig. 1A). The arenas were hence visually isolated, but the birds could still hear each other. On the floor of each arena (350 × 170 cm (L × W)), eight circles (50 cm in diameter) distributed in a 2 × 4 matrix, separated by 20 cm from each other were drawn with black permanent markers (Fig. 1B). In the centre of each circle, a small cup attached to a plywood platform (20 cm × 20 cm) was placed and was used to provide grape pieces as the food reward during the test. Each arena had a start box that was connected to a pulley system allowing the observer to lift both start boxes simultaneously. Both arenas were equipped with a camera (Samsung SCO-2080R, IR, Samsung Techwin CO., Korea) connected to computer screens located outside the arena but within the experimental room allowing simultaneous viewing, recording, and coding of birds’ behaviour by two researchers while birds were tested in parallel using both arenas. The researchers were trained on holeboard test videos obtained from Dumontier et al. (44) to establish inter-observer reliability before the experiment began. For the whole procedure, home pens were randomly assigned to an arena and observing researcher. Once assigned, the birds were always habituated and tested in the same arena and handled and scored by the same researcher (i.e., 8 pens or 24 birds assigned per arena and researcher). The number of grape pieces eaten and each visit of the hen to a cup were scored. If at least half the body of the hen was inside the circle and the head was oriented towards the cup, it was considered a visit.

**Figure 1. Schematic representation of the holeboard arena: (1A) Two birds were tested simultaneously. The black arrows represent the direction of movement of rope and pulley system used to lift the start boxes. (1B, adapted from Dumontier et al.** (44)**) The dimensions of each arena and the general spatial configuration of the cups. The cups were numbered from 1 to 8. The rectangles represent the platforms of the cups.**

*Habituation:* The focal birds were habituated to grape pieces in their home pens using the same cups as in the test. The habituation was performed over two consecutive days, with three sessions of 20 minutes per day. After habituation to the food reward, six focal birds per pen were randomly chosen and habituated to the test arena in groups of three over three sessions. The first session lasted for 10 minutes and the other two was five minutes each. Each cup contained three grape pieces during the group habituation. Following the group habituation, the birds were individually habituated to the arena. Each cup contained one grape during the individual habituation. After three individual habituation sessions of five minutes each, three birds per pen that ate the most grape pieces over the three habituation sessions were selected for the test. The selected birds were further habituated to the arena until they ate from all the cups. The holeboard test was conducted in three separate phases as follows:

*Cued phase (trials 1 - 10):* Three cups out of the eight cups were baited with a piece of grape. To control for the odour cues, a piece of grape was placed in a hidden compartment below the bottom of each unbaited cup. To provide cues associated with the food reward, the plywood platforms were red in colour for the baited cups, while the unbaited cups had no colour cues associated with them. Three configurations of the baited cups (i.e., the spatial position of the baited cups in the arena) were randomly chosen and assigned to the three focal birds selected for the tests from each pen (i.e., one configuration per bird). The birds were tested in a random order on each test day. At the start of each test, the birds were placed under the start boxes in both arenas by the researchers and doors of the arenas were closed. The test began when the start boxes were lifted by one of the researchers and ended when both birds had consumed the food reward from all three cups, or a maximum of five minutes had elapsed. The birds were returned to the home pen after each trial and were given 10 trials over five days to learn the configuration of the rewarded cups.

*Uncued phase (trial 11 – 30):* The same reward configuration and test procedure were used for both the cued and uncued phases, but the baited cups no longer had any visual cues in the latter as the plywood bases of all cups looked the same. The birds were given 20 trials over 10 days to learn the reward configuration without visual cues.

*Reversal phase (trial 31 – 40):* To get to the reversal phase, birds had to reach a success criterion calculated as the ratio of number of visits to baited cups to total number of visits to all cups. The ratio indicates the ability of the bird to distinguish between rewarded and unrewarded cups and can be used as a measure of learning performance (42). Birds that had reached a score of 0.6 or more in three out of four consecutive trials in the uncued phase were subjected to the reversal phase. The reversal phase lasted for 10 trials over five days and the birds had to find the food reward in a new configuration of uncued, baited cups thus measuring the ability of the birds to unlearn the old and learn the new reward configuration. Three new reward configurations were randomly chosen and assigned to the birds that met the success criterion from each pen (i.e., one configuration per bird). Some pens had less than three birds as not all birds met the success criterion.

*Data processing*: For each trial, we performed the following calculations:

1. Reference memory errors: the sum of all visits and revisits to unrewarded cups. The reference memory errors indicate the ability of the birds to discriminate between rewarded and unrewarded cups.

2. Working memory errors: the revisits to rewarded cups (calculated as the difference between the number of rewarded visits and total number of visits to rewarded cups). It reflects the ability of the chickens to avoid re-visits to rewarded cups during a trial.

3. General working memory errors: total number of revisits to all the cups (calculated as the difference between the number of unique visits to rewarded and unrewarded cups and total number of visits to all cups). This indicates the ability of the chickens to avoid re-visits to already visited cups during a trial.

For all memory errors, greater values indicate a poorer performance, with zero being the best possible performance.

4. Trial duration: total time taken to consume the food rewards from all three cups. Duration was measured starting from the lift of the start box with a maximum of five minutes in case the birds failed to consume the rewards from all three rewarded cups.

5. Errors during phase change: to investigate the response of the birds to the change in phases (i.e., cued to uncued, uncued to reversal), we compared the number of memory errors of each bird before and after a phase change for all the three memory indices. For the cued to uncued phase change, we used the last three trials of the cued phase (trial 8 – 10) and first three trials of the uncued phase (trial 11 - 13), and the last three trials (trial 28 -30) and first three trials (31 - 33) of the uncued and reversal phases respectively for the uncued to reversal phase change. As we expected the number of errors to have different directions in slopes in accordance with the phase, we grouped the two extremes of ramp users (i.e., the ten birds with the highest (HR) and the ten birds with the lowest (LR) mean ramp use frequency) to simplify the analysis and interpretation.

**2.4. Statistical analysis**

All analyses were performed with R (version 4.1.1, R Core Team, 2021) and R studio (RStudio Team, 2021) as the graphical interface. We used 'lme4' (45) package to fit linear mixed models (LMM) and package 'glmmTMB' (46) to fit generalized linear mixed effects models (GLMM). The model assumptions for homogeneity of variance and normal distribution of errors were checked using the ‘Dharma’ (47) package. When the model assumptions were not met, the data were transformed. Continuous explanatory variables were centred to zero and categorical variables were sum-contrast coded to set the reference level of contrast calculation as the mean of all groups within a variable. This was done to obtain interpretable main effects, even in the presence of interactions. The p-values of each explanatory variable were calculated by reducing each model by the particular explanatory variable and comparing it to the full model using parametric bootstrap tests (package ‘pbkrtest’ (48)). The model estimates and confidence intervals were calculated using 'broom:mixed' package (49) and the package 'emmeans' was used to calculate estimated marginal means from the full models (50). We used the effect sizes, confidence intervals and p-values to interpret our results. To reduce the numbers of tests, post-hoc comparisons were done qualitatively using numeric and visualizations of the estimated marginal means while also considering the raw data. The repeatability of ramp use was calculated using 'rptR' package (51). The 'tidyverse' package (52) was used for data cleaning and the package 'ggplot2' (53) for data visualization.

*Repeatability of ramp use:* We summed the number of transitions and active behaviours on ramps for individuals to obtain total ramp use. We analysed the general ramp use behaviour of individuals descriptively. To investigate whether individuals showed consistent differences in ramp use behaviour, we calculated the repeatability of ramp use among individuals over all observation days (eight observation bouts per day with seven days for each individual). Repeatability was calculated as the ratio of variance explained by the focal birds to the total within-group (residual) variance (51,54). To calculate repeatability, we split the analysis into two because of the high frequency of zeroes in the data. For the first analysis, we assigned a binary condition for each observation bout for all focal birds and fitted the model with a binary distribution (yes or no; depending on if the focal bird used ramps in that observation bout or not). The second analysis only included the non-zero observations of ramp use by the focal birds and was fit with a Poisson distribution. Additionally, we also calculated repeatability by using mean ramp use over each observation day for each focal bird as a response variable and fitted the model with a Gaussian distribution.

The analyses were performed including all focal birds as well as only including the focal birds that underwent the holeboard test. Each model included treatment as categorical (to account for differences from treatment groups as detailed in Johny et al. (2023) (28)) and DoA as continuous (6, 10, 12, 20, 27, 41, 55 DoA) explanatory variables. Bird ID crossed with date was included as a random factor for each model. Confidence intervals for repeatability estimates were calculated by parametric bootstrapping. We deemed repeatability estimates significant if the 95% confidence intervals excluded zero.

*The association between holeboard measures and ramp use:* For the cued and uncued phase, data from all focal birds that underwent the holeboard task (n = 48) and for reversal phase the data from focal birds that met the success criteria (n = 37) were included in the analysis. The reference, working and general working memory errors, and trial duration for each bird and trial were used as response variables and were analysed separately for each phase. To relate ramp use frequency to holeboard parameters, the mean ramp use frequency (MRF) was calculated by taking mean ramp use over all observation days per focal bird and used as a continuous explanatory variable. Each model contained MRF per individual and trial number (cued: 1 - 10, uncued: 11 - 30, reversal: 31 – 40) as continuous variables as well as their interaction as explanatory variables. Configuration was added as a control variable in each model, as preliminary visualizations revealed differences in holeboard measures between different configurations. Trial number nested in bird ID nested in pen was used as random factor in all models. The models were fit with Poisson distribution for cued and reversal phase for reference memory errors, cued phase for working memory errors, and reversal phase for general working memory errors. Uncued phase of reference memory errors, cued and reversal phase of working memory errors, and cued and uncued phase of general working memory errors were fit with a negative binomial distribution. All phases of trial duration were fit with a Poisson distribution.

The effect of phase change on reference, working, and general working memory errors were analysed separately for both phase changes. The trials and birds for the phase change analysis were chosen as per point five under the 'data processing' section. All models included an interaction of ramp use group (HR, LR) and respective phases (for ex., cued and uncued for cued to uncued phase change analysis) as categorical explanatory variables. Reward configuration was added as a control variable and trial number nested in bird ID nested in pen was included as a random factor in all models.

The data and code for all analysis can be found at <https://osf.io/9j2g8>

**3. Results**

**3.1. Ramp use**

*All focal birds*: The mean frequency of individual ramp use over 56 observations (8 bouts per 7 observation days) was 0.39 uses per observation bout (i.e., per 3 minutes). The mean individual ramp use per bout ranged from 0.09 to 1.00 with a standard deviation of 0.16. Of the 7,186 observations (eight observation bouts from seven days for 128 focal birds), 79% of the observations were zeroes (5,698). There was no repeatability for whether focal birds used ramps in a given observation bout or not (binary outcome, repeatability (r = 0.005, CI [0.00, 0.01]). There was no repeatability in ramp use for focal birds for non-zero observations (r = 0.00, CI [0.00, 0.01]), as well for ramp use averaged over DoA per bird (r = 0.04, CI [0.00, 0.09]).

*Focal birds that underwent holeboard test*: The mean frequency of individual ramp use was 0.38 uses (range 0.09 – 0.67) per observation bout (i.e., three minutes) with a standard deviation of 0.14. Eighty percent (2,153 observations) of the total 2,688 observations (eight observation bouts from seven days for 48 birds) were zero. There was no repeatability for whether birds used ramps or not within an observation bout (r = 0.00, CI [0.00, 0.01]). No repeatability was found for all non-zero observations of ramp use (r = 0.00, CI [0.00, 0.04]) and ramp use averaged per DoA per bird (r = 0.00, CI [0.00, 0.01]).

**3.2. Holeboard test**

*Reference memory errors:* Birds with higher MRF demonstrated a decrease in errors compared to birds with lower MRF during the cued phase, although statistical evidence was limited (model estimates (Est) ± CI = 0.94 [0.88, 0.99], p = 0.08, Fig. 2). The bird with the lowest MRF made slightly more errors [Estimated marginal means (EMM) ± CI = 3.36 [2.52, 4.48]) than the ones with median (2.95 [2.61, 3.35]) and highest (2.55 [1.91, 3.41]) MRF. The number of errors also decreased with trial number (Est ± CI = 0.75 [0.76, 0.85], p = 0.001) in the cued phase. The interaction of MRF and trial number did not seem to influence the number of errors made in the cued phase (Est ± CI = 0.97 [0.32, 0.92], p = 0.31). In the uncued phase, the number of errors reduced with trial number (Est ± CI = 0.93 [0.89, 1.07], p = 0.001), but there were no influences of MRF (Est ± CI = 1.01 [0.96, 1.07], p = 0.74) or the interaction between MRF and trial number (Est ± CI = 1.00 [0.97, 1.04], p = 0.98). In the reversal phase, the birds with higher MRF made more errors than the birds with lower MRF (Est ± CI = 1.10 [1.01, 1.20], p = 0.05, Fig. 3). The bird with the lowest MRF made slightly fewer errors (EMM ± CI = 2.72 [1.82, 4.07]) than the ones with median (3.30 [2.80, 3.90] and high (4.01 [2.78, 5.77]) MRF. With limited statistical support, the number of errors slightly decreased with trial number (Est ± CI = 0.95 [0.9, 1.01], p = 0.08) in the reversal phase. The interaction of MRF and trial number did not have any effect on the number of errors made in the reversal phases (Est ± CI = 0.99 [0.94, 1.04], p = 0.82).

**Figure 2: The relationship between reference memory errors (sum of all visits and revisits to unrewarded cups) and mean individual ramp use frequency in the cued phase (model estimates ± CI = 0.94 [0.88, 0.99], p = 0.08). The black points connected by the black line represent the estimated marginal means and the shaded ribbon represents 95 % confidence intervals. The coloured points represent reference memory errors (all visits – visits to rewarded cups) of focal birds’ colour-graded according to mean ramp use frequency.**

**Figure 3: The relationship between reference memory errors (sum of all visits and revisits to unrewarded cups) and mean individual ramp use frequency in the reversal phase (model estimates ± CI = 1.10 [1.01, 1.20], p = 0.05). The black points connected by the black line represent the estimated marginal means and the shaded ribbon represents 95 % confidence intervals. The coloured points represent reference memory errors (all visits – visits to rewarded cups) of focal birds colour-graded according to mean ramp use frequency.**

*Working memory:* In general, all birds made very few working memory errors throughout all phases (Mean ± SD: cued – 0.33 ± 0.69, uncued – 0.23 ± 0.64, reversal – 0.34 ± 0.74). With weak statistical support, the birds with higher MRF made marginally fewer errors than birds with a lower MRF (Est ± CI = 0.77 [0.59, 1.00], p = 0.06, Fig. 4) in the cued phase. The bird with the lowest MRF (EMM ± CI = 0.47 [0.15, 1.45]) made slightly more errors than the ones with median (EMM ± CI = 0.27 [0.15, 0.47]) and the one with the highest MRF (EMM ± CI = 0.15 [0.05, 0.52]). There was a slight reduction in the number of errors as the trial number increased, although the statistical support for this find was weak (Est ± CI = 0.84 [0.69, 1.03], p = 0.08). The interaction between trial number and MRF did not a have an effect number of working memory errors in the cued phase (Est ± CI = 0.94 [0.78, 1.15], p = 0.80). In the uncued phase, the number of errors decreased with trial number (Est ± CI = 0.87 [0.76, 0.99], p = 0.05). No influence of MRF (Est ± CI = 1.07 [0.86, 1.39], p = 0.12) or the interaction between MRF and trial number (Est ± CI = 0.95 [0.84, 1.08], p = 0.45) was found on the number of errors made in the uncued phase. In the reversal phase, the number of errors made by the birds was not influenced by MRF (Est ± CI = 1.03 [0.78, 1.36], p = 0.87), trial number (Est ± CI = 0.83 [0.67, 1.06], p = 0.14) or their interaction (Est ± CI = 0.84 [0.66, 1.05], p = 0.14).

**Figure 4: The relationship between working memory errors (sum of all revisits to rewarded cups) and mean individual ramp use frequency in the cued phase (model estimates ± CI = 0.77 [0.59, 1.00], p = 0.06). The black points connected by the black line represent the estimated marginal means and the shaded ribbon represents 95 % confidence intervals. The coloured points represent working memory errors (all visits to rewarded cups – rewarded visits) of focal birds colour-graded according to mean ramp use frequency.**

*General working memory:* Birds made very few general working memory errors in all three phases (Mean ± SD: cued – 0.73 ± 1.56, uncued – 0.62 ± 1.55, reversal – 0.86 ± 1.61). The number of errors made by birds decreased with trial number with varying statistical support for each phase: cued (Est ± CI = 0.69 [0.56, 0.84], p = 0.003), uncued (Est ± CI = 0.85 [0.72, 1.04], p= 0.08) and reversal (Est ± CI = 0.87 [0.78, 0.97], p = 0.02). No influences of MRF (Est ± CI, cued = 0.80 [0.61, 1.04], p = 0.12, uncued = 1.10 [0.86, 1.39], p = 0.46, reversal = 1.17 [0.90, 1.53], p = 0.30) and the interaction between MRF and trial number (Est ± CI, cued = 0.89 [0.70, 1.10], p = 0.28, uncued = 0.99 [0.83, 1.19], p = 0.93, reversal = 0.94 [0.84, 1.06], p = 0.30) were found for any of the phases.

*Trial duration:* The trials in which the birds failed to consume from all baited cups were removed from the analysis (9, 4 and 1 observations from cued, uncued and reversal phases, respectively). Trial duration was influenced by an interaction between MRF and trial number (Est ± CI = 0.88 [0.84, 0.93], p = 0.001) in the cued phase, with birds with a high MRF showing a faster decline in trial duration with increasing trial number (Estimated slopes ± CI for selected MRF: lowest = - 0.19 [- 0.24, - 0.13], median = - 0.29[- 0.32, -0.27], highest = - 0.40 [- 0.45, −0.34]). Trial duration decreased with increasing trial number in the uncued (Est ± CI = 0.99 [0.98, 1.00], p = 0.001) and reversal (Est ± CI = 0.93 [0.91, 0.95], p = 0.001) phases. No effect of MRF (uncued = 1.05 [0.53, 2.02], p = 0.33, reversal = 0.18 [0.02, 2.03], p = 0.20) nor the interaction of MRF and trial number (uncued = 1.00 [0.99, 1.02], p = 0.78, reversal = 1.07 [1.01, 2.03], p = 0.79) were found for trial duration for the uncued and reversal phases.

*Phase change:* The interaction between phase change and ramp use group (i.e., high (HR) and low (LR) ramp user) had an effect on the reference memory errors (p = 0.007) for the cued to uncued phase change with HR birds making more errors than the LR birds for this phase change (Estimated marginal means (EMM) presented in Table 1). For the uncued to reversal phase change, there was an effect of the interaction between phase change and ramp use group on the reference errors (p = 0.02) with HR birds making more errors than LR birds with phase change (EMM in Table 1). The interaction between phase change and ramp use group had no effect on the working memory scores for both phase changes (cued to uncued, p = 0.21, uncued to reversal, p = 0.32). For the general working memory, the interaction between phase change and ramp use group showed an effect for the cued to uncued (p = 0.006), but not for the uncued to reversal phase change (p = 0.16). The HR birds made more errors than LR birds as the phase changed from cued to uncued for the general working memory (EMM in Table 1).

**Table 1: Estimated marginal means for the interaction between ramp use group (high vs. low ramp users) and phase ­for the phase change analysis of reference and general working memories from the holeboard test.**

| Holeboard measure | Phase change analysis | Phase | Ramp use group | Estimated marginal means (EMM) ± CI |
| --- | --- | --- | --- | --- |
| Reference memory | Cued to uncued | Cued | High ramp use | 1.82 [1.40, 2.36] |
|  |  |  | Low ramp use | 2.64 [2.08, 3.36] |
|  |  | Uncued | High ramp use | 2.93 [2.52, 3.40] |
|  |  |  | Low ramp use | 2.92 [2.45, 3.48] |
|  | Uncued to reversal | Uncued | High ramp use | 2.93 [2.52, 3.40] |
|  |  |  | Low ramp use | 2.85 [2.45, 3.31] |
|  |  | Reversal | High ramp use | 4.01 [3.22, 4.99] |
|  |  |  | Low ramp use | 2.46 [1.85, 3.28] |
| General working memory | Cued to uncued | Cued | High ramp use | 0.15 [0.06, 0.38] |
|  |  |  | Low ramp use | 0.60 [0.29, 1.26] |
|  |  | Uncued | High ramp use | 0.42 [0.23, 0.77] |
|  |  |  | Low ramp use | 0.29 [0.06, 0.38] |

**4. Discussion**

The aim of the study was to describe the relationship between inter-individual variation in early life ramp use and spatial cognition in laying hen pullets. Even though ramp use behaviour was not consistent across the early life period for individual birds, we found that the spatial cognitive abilities of the birds were weakly associated to individual differences in ramp use over the first eight WoA. We found that the birds that used ramps more made fewer reference and working memory errors in the cued phase than birds with lower ramp use. However, birds with higher ramp use made more reference memory errors in the reversal phase. The birds that used ramps more often also showed a faster decline in time taken to solve the holeboard test with increasing trial numbers in the cued phase. For the phase change analysis, we found that birds with higher ramp use made more reference memory errors in both phase changes and, more general working memory errors in the cued to uncued phase change than the birds that used ramps less often.

**Early life ramp use is associated with spatial cognitive measures from the holeboard test**

As exposure to a complex environment as well as voluntary exercise in early life have been shown to improve spatial cognition in a range of taxa, it could be expected that birds with higher ramp use might have better reference, working and general working memory scores than the ones with lower ramps use. Our findings indicate an association between early-life ramp utilization and cognition; however, they do not provide a definitive indication that using ramps in early life enhances spatial cognition in laying hen pullets. Therefore, our results remain open to various interpretations, allowing for multiple explanations.

One possible explanation might be that the individual differences in both ramp use and cognition might be driven by consistent inter-individual differences in behaviour. Consistent differences in behaviours such as boldness, exploration and activity have been shown to correlate to movement in animals (55,56). For example, bolder bank voles (57) and Trinidad killifish (58) move longer distances, fast exploring female great tits disperse to a greater distance (59), and high exploratory laying hens use the outdoor range more than the ones that are less exploratory (60,61). It is possible that individual differences in ramp use might also be driven by individual differences in activity, exploration, and fearfulness, with more active, explorative, and less fearful birds showing more use of ramps.

The speed-accuracy trade-off proposed by Sih and Del Giudice (62) is one of the main frameworks for explaining the relationship between consistent inter-individual differences in behaviour and cognition. It predicts that fast behavioural types (with high exploration and activity levels, neophilia, etc.) tend to prioritize speed over accuracy and learn simple tasks such as associative learning faster. However, they are less cognitively flexible and make more mistakes during challenging tasks, such as reversal learning. In contrast, slow behavioural types (with low exploration and activity levels, neophobia, etc.) tend to prioritize accuracy over speed, and while they are slower at simple learning tasks, they are more sensitive to changes in their environment and make fewer errors during reversal tests compared to fast behavioural types. Although the speed-accuracy trade-off is not studied in domestic hens, our results seem to align with this model because the cognitive styles of individuals with high and low ramp use resembles the one of fast and slow behavioural types, respectively.

More specifically, birds with higher ramp use made slightly fewer reference and working memory errors, and learned the task faster compared to individuals with lower ramp use in the cued phase, which can be viewed as a simple associative learning task because the rewarded cups were associated with colour cues. In contrast, birds with lower ramp use made fewer reference memory errors during the reversal phase, which is a more cognitively challenging task. Birds with lower ramp use also made fewer reference and general working memory errors in the cued to uncued phase change than the birds that used ramps more often. The lower number of reference memory errors, both during the reversal phase as well as in the phase changes, might imply that the birds with lower ramp use frequency show higher behavioural flexibility and are able to update their learning strategy with changes in environment. Lastly, marginally higher working memory errors of the birds with low ramp use in the cued phase might be also indicative of neophobia as cues were not present during the habituation.

Empirical research however indicates that the relationship between consistent inter-individual differences in behaviour and cognition does not always follow the speed-accuracy trade-off framework and that the relationship is dependent on factors such as species, sex, and age (63). While our results seem to align with the speed-accuracy trade-off framework, studies in red jungle fowl have revealed the complexity of this relationship. For example, Zidar et.al (64) investigated the link between cognition and consistent individual differences in behaviour in red jungle fowl by conducting various learning (discriminative, reversal, and spatial learning) and behavioural tests (novel arena, novel object) on both young and adult red jungle fowl. While there was no correlation between learning speed and personality traits in discrimination and spatial learning tasks, results showed a complex and age-dependent relationship between cognition and behavioural type. When tested at a young age, chicks with higher exploration levels showed better performance in the reversal task than those with lower levels. The direction changed when the birds grew older as less exploratory adult females performed better in the reversal test than highly exploratory females. Age-related changes in exploratory behaviour of red jungle fowl have also been shown by Favati et al (2016) (65), who reported that exploration changes across major developmental stages, such as independence from the mother at around eight weeks of age. In our study, ramp use behaviour was recorded from one to eight WoA and the birds were tested for spatial cognitive skills from nine to 13 WoA. As domestic hens and red jungle fowl are closely related, the consistency in behaviours such as exploration and activity in domestic hens might vary similarly depending on age and ontogenetic changes as demonstrated in the red jungle fowl (65). A potential shift in traits like exploration and activity levels at eight WoA could imply that the use of ramps, as well as its connection to cognitive traits, may have undergone changes from the period of measuring ramp use (1-8 WoA) to the assessment of cognitive traits (9-13 WoA). We thus need further studies across several different developmental stages to elucidate the link between consistent inter-individual differences in behaviour and cognition in laying hens.

Another possible explanation for our results could be that early-life ramp use promotes better spatial cognitive abilities. Birds with higher ramp use might have learned the holeboard test better than those with lower ramp use, as indicated by fewer errors in reference and working memories and a faster decline in trial duration during the cued phase. Furthermore, it was observed that birds with higher ramp use made more reference memory errors during the transition from uncued to reversal phase, whereas no difference in errors was evident among birds with lower ramp use during this phase transition. This outcome suggests that birds with lower ramp use might not have acquired knowledge of the rewarded locations to the same degree as their counterparts with higher ramp use during preceding phases. Lower number of reference memory errors in the reversal phase for the birds with low ramp use could similarly be attributed to the aforementioned rationale. However, overall, no learning differences between high and low ramp users were observed in the uncued phase. The lack of difference may be due to both groups of birds checking the unrewarded cups on their way to the rewarded cups, resulting in similar scores since the cost incurred in the process is low (22,44). Therefore, the scores in the uncued phase may not accurately reflect the learning performance of the birds.

**Consistency in ramp use behaviour**

We expected ramp use to be repeatable, as previous work has shown that adult laying hens show long-term consistency in the daily vertical distance travelled in a multi-tier aviary (66). In addition, ramp use might also be driven by differences in exploration and activity, which has been shown to have consistent individual differences in domestic hens (exploration: 68, 65, activity: 69). However, our findings revealed no consistent variations among individuals in terms of ramp use. It is worth noting that individual behaviours in many species tend to be unstable during ontogeny and only become consistent after reaching maturity (69). For instance, the consistency of daily vertical distance travelled in an aviary system was low before reaching peak egg production (r = 0.38, WoA 18 - 19, (70)) and only stabilized after sexual maturation (r = 0.66 for WoA 25 – 60, (66)). Favati et al. (65) found short-term consistency for exploration and foraging behaviour in the early life (4–8 weeks) of red jungle fowl but behavioural traits such as exploration and response to predators were inconsistent until sexual maturation and only stabilized during adulthood. Similarly, Neave et. al (71) showed that individual consistency was poor across the major developmental periods of puberty for cattle (pre weaning to lactation), but showed short-term consistency during the early rearing period (pre- to post-weaning) and late period of development (puberty to lactation). Furthermore, the video analysis conducted in this study utilized relatively short observation periods (specifically, 3 minutes), which might have failed to capture the behavioural variation in ramp use among the birds, considering that 80% of the observations were zeroes. Although our study did not demonstrate the repeatability of ramp use during the early life period of laying hens, it is reasonable to expect short-term consistency in ramp use during this phase. Nonetheless, the results highlight the need for longer observation bouts to effectively capture the variation between individuals.

**Evaluation of holeboard task**

Unlike in rodents and pigs, the holeboard task is not widely used to measure the spatial cognition of hens and hence require a through consideration of the variations and limitations of the methods. Our holeboard test setup was similar to Dumontier et al. (44), except for the lack of two start box positions in the arena and the order of the phases as it was unuced, cued, overtraining and reversal phase for Dumontier et al. (44). Also, instead of including an overtraining phase as used by Dumontier et al. (44), we used a success criteria to make sure the birds had learnt the spatial location of the rewards before continuing with the reversal phase. We calculated memory errors rather than memory ratios like Dumontier et al. (44), because of the low variation in the working memory and general working memory ratios in our study. Our birds showed relatively high working (mean ± SD = 0.94 ± 0.13) and general working memory scores (0.93 ± 13) which were similar to Dumontier et al. (44) (working memory = 0.8 – 0.9, general working memory = 0.7 – 0.9), but slightly better than in more complex holeboard tests with a 3 × 3 matrix of cups (working memory = 0.7 – 0.8 (43) and 0.6 – 0.9 in (22)). The reference memory in our study was 0.55 ± 0.13, which again was similar to other studies (0.4 – 0.7; (23,44,45)). The similarity in scores with other studies, coupled with the decrease in reference memory errors throughout all phases suggest that the birds did learn the task.

As noted by Dumontier et al. (44) and Tahamtani et al. (22), one general criticism of the holeboard test as a method might be the low cost to check the unrewarded cups. There was only a marginal decrease in reference memory errors in the uncued phase from trial 11 to 30. The reference memory errors also showed a huge variation across consecutive trials, which might be due to birds checking the all the cups in certain trials, as it incurs only a negligible energy expenditure. Another limitation specific to our study is the use of one start location throughout the test, which can result in egocentric approaches such as fixed food search patterns (42). In addition, the rectangular arena with a 2×4 matrix distribution of rewards, can lead to fixed simple food search patterns such as visiting the closest cup straight ahead.

**Outlook**

The current study contributes to the existing body of research exploring the relationship between early-life habitat complexity and spatial cognition in laying hens. The existing body of knowledge has covered multiple levels of complexity (cage vs aviary, floor vs three-dimensional rearing) and ages for a set of spatial cognitive indices (spatial long and short-term memories, orientation, depth perception, neuro-motor coordination). Nonetheless, the long and short-term impacts of early life habitat complexity on the spatial cognition of laying hens remains ambiguous. Future studies should account for effect of age, type of test used and include additional measures of brain development such as neural cell counts to better understand the impact early life habitat complexity has on the development of spatial cognition in laying hens. Furthermore, it is important to investigate the specific level of habitat complexity required to induce developmental changes in the spatial cognitive abilities of the birds. This aspect deserves further investigation to determine the optimal conditions for promoting spatial cognition in laying hens.

**Acknowledgements**

We thank Abdulsatar Abdel Rahman for helping with the holeboard task. Many thanks to Thomas Heinzel and Jan Büchler, for building the pens and arena, Masha Marincek and Josie Siegel for their help with the video analysis. We thank Lucille Dumontier for the discussions on the statistical analysis. The work was supported by the European Union’s Horizon 2020 research and innovation programme under the Marie Skłodowska-Curie grant agreement No 812777. The document reflects only the author's view and the European Union’s Horizon 2020 research, and innovation programme is not responsible for any use that may be made of the information it contains.

**References**

1.	Thinus-Blanc C. Animal Spatial Cognition. Anim Spat Cogn. 1996 Nov;

2.	De Pasquale C, Neuberger T, Hirrlinger AM, Braithwaite VA. The influence of complex and threatening environments in early life on brain size and behaviour. Proc R Soc B Biol Sci. 2016 Jan 27;283(1823).

3.	Buchanan KL, Grindstaff JL, Pravosudov V V. Condition dependence, developmental plasticity, and cognition: implications for ecology and evolution. Trends Ecol Evol. 2013 May 1;28(5):290–6.

4.	Salvanes AGV, Moberg O, Ebbesson LOE, Nilsen TO, Jensen KH, Braithwaite VA. Environmental enrichment promotes neural plasticity and cognitive ability in fish. Proc R Soc B Biol Sci. 2013 Sep 22;280(1767).

5.	Whiteside MA, Sage R, Madden JR. Multiple behavioural, morphological and cognitive developmental changes arise from a single alteration to early life spatial environment, resulting in fitness consequences for released pheasants. R Soc Open Sci. 2016 Mar 23;3(3).

6.	Falkenberg T, Mohammed AK, Henriksson B, Persson H, Winblad B, Lindefors N. Increased expression of brain-derived neurotrophic factor mRNA in rat hippocampus is associated with improved spatial memory and enriched environment. Neurosci Lett. 1992 Apr 13;138(1):153–6.

7.	Mes D, Van Os R, Gorissen M, Ebbesson LOE, Finstad B, Mayer I, et al. Effects of environmental enrichment on forebrain neural plasticity and survival success of stocked Atlantic salmon. J Exp Biol. 2019 Dec 1;22(23).

8.	van Praag H, Kempermann G, Gage FH. Neural consequences of enviromental enrichment. Nat Rev Neurosci 2000 13. 2000;1(3):191–8.

9.	Morandi-Raikova A, Mayer U. Spatial cognition and the avian hippocampus: Research in domestic chicks. Front Psychol. 2022 Sep 23;13:5791.

10.	Rosa-Salva O, Mayer U, Versace E, Hébert M, Lemaire BS, Vallortigara G. Sensitive periods for social development: Interactions between predisposed and learned mechanisms. Cognition. 2021 Aug 1;213:104552.

11.	Della Chiesa A, Pecchia T, Tommasi L, Vallortigara G. Multiple landmarks, the encoding of environmental geometry and the spatial logics of a dual brain. Anim Cogn. 2006 Oct 29;9(4):281–93.

12.	Vallortigara G. Learning of colour and position cues in domestic chicks: Males are better at position, females at colour. Behav Processes. 1996 Jun 1;36(3):289–96.

13.	Rashid N, Andrew RJ. Right hemisphere advantage for topographical orientation in the domestic chick. Neuropsychologia. 1989 Jan 1;27(7):937–48.

14.	Morandi-Raikova A, Vallortigara G, Mayer U. The use of spatial and local cues for orientation in domestic chicks (Gallus gallus). Anim Cogn 2020 232. 2020 Jan 1;23(2):367–87.

15.	Gunnarsson S, Yngvesson J, Keeling LJ, Forkman B. Rearing without early access to perches impairs the spatial skills of laying hens. Appl Anim Behav Sci. 2000 Apr 3;67(3):217–28.

16.	Freire R, Cheng HW, Nicol CJ. Development of spatial memory in occlusion-experienced domestic chicks. Anim Behav. 2004 Jan 1;67(1):141–50.

17.	Freire R, Cheng HW. Experience-dependent changes in the hippocampus of domestic chicks: A model for spatial memory. Eur J Neurosci. 2004 Aug;20(4):1065–8.

18.	Ferreira VHB, Guesdon V, Calandreau L. How can the research on chicken cognition improve chicken welfare: a perspective review. https://doi.org/101080/0043933920211924920. 2021;77(3):679–98.

19.	Campbell DLM, Talk AC, Loh ZA, Dyall TR, Lee C. Spatial Cognition and Range Use in Free-Range Laying Hens. Anim 2018, Vol 8, Page 26. 2018 Feb 8;8(2):26.

20.	Leenstra F, Ten Napel J, Visscher J, Van Sambeek F. Layer breeding programmes in changing production environments: a historic perspective. Worlds Poult Sci J. 2016 Mar 1;72(1):21–36.

21.	Schuck-Paim C, Negro-Calduch E, Alonso WJ. Laying hen mortality in different indoor housing systems: a meta-analysis of data from commercial farms in 16 countries. Sci Reports 2021 111. 2021 Feb 4;11(1):1–13.

22.	Tahamtani FM, Nordgreen J, Nordquist RE, Janczak AM. Early life in a barren environment adversely affects spatial cognition in laying hens (Gallus gallus domesticus). Front Vet Sci. 2015 Mar 18;2(MAR).

23.	Muir GD, Gosline JM, Steeves JD. Ontogeny of bipedal locomotion: walking and running in the chick. J Physiol. 1996 Jun 1;493(2):589–601.

24.	Muir GD. Early ontogeny of locomotor behaviour: a comparison between altricial and precocial animals. Brain Res Bull. 2000 Nov 15;53(5):719–26.

25.	Thiele H-H. Management recommendations for rearing pullets for alternative housing systems. Lohmann Inf. 2007;42:14–24.

26.	Stratmann A, Guggisberg D, Benavides-Reyes C, Siegford J, Toscano MJ. Providing ramps in rearing aviaries affects laying pullet distribution, behavior and bone properties. J Appl Poult Res. 2022 Dec 1;31(4):100283.

27.	Norman KI, Weeks CA, Tarlton JF, Nicol CJ. Rearing experience with ramps improves specific learning and behaviour and welfare on a commercial laying farm. Sci Rep. 2021 Apr 23;11(1):1–14.

28.	Johny A, Guggisberg D, Toscano MJ, Stratmann A. This is the way: The effect of artificial cues on early life ramp use behaviour of laying hen chicks. Appl Anim Behav Sci. 2023 Mar 1;260:105873.

29.	Van Praag H, Christie BR, Sejnowski TJ, Gage FH. Running enhances neurogenesis, learning, and long-term potentiation in mice. Proc Natl Acad Sci U S A. 1999 Nov 9;96(23):13427–31.

30.	Van Praag H. Neurogenesis and exercise: Past and future directions. NeuroMolecular Med. 2008 Jun 20;10(2):128–40.

31.	Merkley CM, Jian C, Mosa A, Tan YF, Wojtowicz MM. Homeostatic regulation of adult hippocampal neurogenesis in aging rats: long-term effects of early exercise. Front Neurosci. 2014;8(8 JUL).

32.	Hillman CH, Erickson KI, Kramer AF. Be smart, exercise your heart: exercise effects on brain and cognition. Nat Rev Neurosci 2008 91. 2008 Jan;9(1):58–65.

33.	Sibley BA, Etnier JL. The Relationship between Physical Activity and Cognition in Children: A Meta-Analysis. Pediatr Exerc Sci. 2003 Aug 1;15(3):243–56.

34.	Rossi GS, Wright PA. Does leaving water make fish smarter? Terrestrial exposure and exercise improve spatial learning in an amphibious fish. Proc R Soc B. 2021 Jun 30;288(1953).

35.	Mes D, Palstra AP, Henkel C V., Mayer I, Vindas MA. Swimming exercise enhances brain plasticity in fish. R Soc Open Sci. 2020 Jan 1;7(1).

36.	Fiaz AW, Léon-Kloosterziel KM, Leeuwen JL van, Kranenbarg S. Exploring the effect of exercise on the transcriptome of zebrafish larvae (Danio rerio). J Appl Ichthyol. 2014 Aug 1;30(4):728–39.

37.	Boogert NJ, Reader SM, Laland KN. The relation between social rank, neophobia and individual learning in starlings. Anim Behav. 2006 Dec;72(6):1229–39.

38.	Shaw RC, Schmelz M. Cognitive test batteries in animal cognition research: evaluating the past, present and future of comparative psychometrics. Anim Cogn. 2017 Nov 1;20(6):1003–18.

39.	Rufener C, Berezowski J, Maximiano Sousa F, Abreu Y, Asher L, Toscano MJ. Finding hens in a haystack: Consistency of movement patterns within and across individual laying hens maintained in large groups. Sci Rep. 2018 Dec 1;8(1).

40.	Gómez Y, Berezowski J, Jorge YA, Gebhardt-Henrich SG, Vögeli S, Stratmann A, et al. Similarity in Temporal Movement Patterns in Laying Hens Increases with Time and Social Association. Animals. 2022 Mar 1;12(5):555.

41.	Armstrong EA, Voelkl B, Voegeli S, Gebhardt-Henrich SG, Guy JH, Sandilands V, et al. Cell Proliferation in the Adult Chicken Hippocampus Correlates With Individual Differences in Time Spent in Outdoor Areas and Tonic Immobility. Front Vet Sci. 2020 Aug 26;7:587.

42.	van der Staay FJ, Gieling ET, Pinzón NE, Nordquist RE, Ohl F. The appetitively motivated “cognitive” holeboard: A family of complex spatial discrimination tasks for assessing learning and memory. Neurosci Biobehav Rev. 2012 Jan 1;36(1):379–403.

43.	Nordquist RE, Heerkens JLT, Rodenburg TB, Boks S, Ellen ED, van der Staay FJ. Laying hens selected for low mortality: Behaviour in tests of fearfulness, anxiety and cognition. Appl Anim Behav Sci. 2011 May 1;131(3–4):110–22.

44.	Dumontier L, Janczak AM, Smulders T V., Nordgreen J. Effects of the rearing environment complexity on laying hens’ spatial cognition: A holeboard test approach. Appl Anim Behav Sci. 2023 Mar 1;260:105878.

45.	Bates D, Mächler M, Bolker BM, Walker SC. Fitting linear mixed-effects models using lme4. J Stat Softw. 2015;67(1).

46.	Brooks ME, Kristensen K, van Benthem KJ, Magnusson A, Berg CW, Nielsen A, et al. glmmTMB balances speed and flexibility among packages for zero-inflated generalized linear mixed modeling. R J. 2017 Dec 1;9(2):378–400.

47.	Hartig F. DHARMa: Residual Diagnostics for Hierarchical (Multi-Level / Mixed) Regression Models. R package version 0.2.0. https://CRANR-project.org/package=DHARMa. 2020;

48.	Halekoh U, Højsgaard S. A Kenward-Roger Approximation and Parametric Bootstrap Methods for Tests in Linear Mixed Models – The R Package pbkrtest. J Stat Softw. 2014 Sep 12;59(9):1–32.

49.	Bolker B, Robinson D. Broom. mixed: Tidying methods for mixed models. R Packag version 02. 2020;6.

50.	Lenth R, Singmann H, Love J, Buerkner P, Herve M. Package ‘emmeans’. 2019.

51.	Stoffel MA, Nakagawa S, Schielzeth H. rptR: repeatability estimation and variance decomposition by generalized linear mixed‐effects models. Goslee S, editor. Methods Ecol Evol. 2017 Nov 30;8(11):1639–44.

52.	Wickham H, Averick M, Bryan J, Chang W, McGowan L, François R, et al. Welcome to the Tidyverse. J Open Source Softw. 2019 Nov 21;4(43):1686.

53.	Wickham H, Chang W. Package ggplot2: An Implementation of the Grammar of Graphics. Creat elegant data Vis using Gramm Graph. 2016;v. 2(1):1–189.

54.	Hertel AG, Hertel AG, Niemelä PT, Dingemanse NJ, Mueller T, Mueller T. A guide for studying among-individual behavioral variation from movement data in the wild. Mov Ecol. 2020 Jun 29;8(1):1–18.

55.	Spiegel O, Leu ST, Bull CM, Sih A. What’s your move? Movement as a link between personality and spatial dynamics in animal populations. Ecol Lett. 2017 Jan 1;20(1):3–18.

56.	Shaw AK. Causes and consequences of individual variation in animal movement. Mov Ecol 2020 81. 2020 Feb 17;8(1):1–12.

57.	Schirmer A, Herde A, Eccard JA, Dammhahn M. Individuals in space: personality-dependent space use, movement and microhabitat use facilitate individual spatial niche specialization. Oecologia. 2019;189:647–60.

58.	Fraser DF, Gilliam JF, Daley MJ, Le AN, Skalski GT. Explaining Leptokurtic Movement Distributions: Intrapopulation Variation in Boldness and Exploration. Am Nat. 2001;158(2):124–35.

59.	Dingemanse NJ, Both C, Van Noordwijk AJ, Rutten AL, Drent PJ. Natal dispersal and personalities in great tits (Parus major). Proc R Soc London Ser B Biol Sci. 2003;270(1516):741–7.

60.	Kolakshyapati M, Taylor PS, Hamlin A, Sibanda TZ, Vilela J de S, Ruhnke I. Frequent Visits to an Outdoor Range and Lower Areas of an Aviary System Is Related to Curiosity in Commercial Free-Range Laying Hens. Anim 2020, Vol 10, Page 1706. 2020 Sep 21;10(9):1706.

61.	Campbell DLM, Hinch GN, Downing JA, Lee C. Fear and coping styles of outdoor-preferring, moderate-outdoor and indoor-preferring free-range laying hens. Appl Anim Behav Sci. 2016 Dec 1;185:73–7.

62.	Sih A, Del Giudice M. Linking behavioural syndromes and cognition: a behavioural ecology perspective. Philos Trans R Soc B Biol Sci. 2012;367(1603):2762–72.

63.	Dougherty LR, Guillette LM. Linking personality and cognition: a meta-analysis. Philos Trans R Soc B Biol Sci. 2018 Sep 26;373(1756).

64.	Zidar J, Balogh A, Favati A, Jensen P, Leimar O, Sorato E, et al. The relationship between learning speed and personality is age- and task-dependent in red junglefowl. Behav Ecol Sociobiol. 2018 Oct 1;72(10):1–13.

65.	Favati A, Zidar J, Thorpe H, Jensen P, Løvlie H. The ontogeny of personality traits in the red junglefowl, Gallus gallus. Behav Ecol. 2016 Jan 1;27(2):484–93.

66.	Montalcini CM, Petelle MB, Toscano MJ. Commercial laying hens exhibit long-term consistent individual differences and behavioural syndromes in spatial traits. R Soc Open Sci. 2023 May 24;10(5).

67.	de Haas EN, Lee C, Hernandez CE, Naguib M, Rodenburg TB. Individual differences in personality in laying hens are related to learning a colour cue association. Behav Processes. 2017 Jan 1;134:37–42.

68.	Rufener C, Berezowski J, Maximiano Sousa F, Abreu Y, Asher L, Toscano MJ. Finding hens in a haystack: Consistency of movement patterns within and across individual laying hens maintained in large groups.

69.	Cabrera D, Nilsson JR, Griffen BD. The development of animal personality across ontogeny: a cross-species review. Anim Behav. 2021 Mar 1;173:137–44.

70.	Montalcini C, Toscano MJ, Petelle M. Scope of consistent inter-individual differences in mean movement behavior within a commercial aviary early on. Meas Behav 2022.

71.	Neave HW, Costa JHC, Weary DM, von Keyserlingk MAG. Long-term consistency of personality traits of cattle. R Soc Open Sci. 2020 Feb 1;7(2).
